# HIV among People Who Inject Drugs in the Middle East and North Africa: Systematic Review and Data Synthesis

**DOI:** 10.1371/journal.pmed.1001663

**Published:** 2014-06-17

**Authors:** Ghina R. Mumtaz, Helen A. Weiss, Sara L. Thomas, Suzanne Riome, Hamidreza Setayesh, Gabriele Riedner, Iris Semini, Oussama Tawil, Francisca Ayodeji Akala, David Wilson, Laith J. Abu-Raddad

**Affiliations:** 1 Infectious Disease Epidemiology Group, Weill Cornell Medical College - Qatar, Cornell University, Qatar Foundation - Education City, Doha, Qatar; 2 Department of Infectious Disease Epidemiology, Faculty of Epidemiology and Population Health, London School of Hygiene & Tropical Medicine, London, United Kingdom; 3 Joint United Nations Programme on HIV/AIDS Regional Support Team, Middle East and North Africa, Cairo, Egypt; 4 Regional Office of the Eastern Mediterranean, World Health Organization, Cairo, Egypt; 5 Human Development Sector, Middle East and North Africa Region, World Bank, Washington (D.C.), United States of America; 6 Global HIV/AIDS Program, World Bank, Washington (D.C.), United States of America; 7 Department of Healthcare Policy and Research, Weill Cornell Medical College, Cornell University, New York, New York, United States of America; 8 Vaccine and Infectious Disease Division, Fred Hutchinson Cancer Research Center, Seattle, Washington, United States of America; Johns Hopkins University, United States of America

## Abstract

**Background:**

It is perceived that little is known about the epidemiology of HIV infection among people who inject drugs (PWID) in the Middle East and North Africa (MENA). The primary objective of this study was to assess the status of the HIV epidemic among PWID in MENA by describing HIV prevalence and incidence. Secondary objectives were to describe the risk behavior environment and the HIV epidemic potential among PWID, and to estimate the prevalence of injecting drug use in MENA.

**Methods and Findings:**

This was a systematic review following the PRISMA guidelines and covering 23 MENA countries. PubMed, Embase, regional and international databases, as well as country-level reports were searched up to December 16, 2013. Primary studies reporting (1) the prevalence/incidence of HIV, other sexually transmitted infections, or hepatitis C virus (HCV) among PWIDs; or (2) the prevalence of injecting or sexual risk behaviors, or HIV knowledge among PWID; or (3) the number/proportion of PWID in MENA countries, were eligible for inclusion. The quality, quantity, and geographic coverage of the data were assessed at country level. Risk of bias in predefined quality domains was described to assess the quality of available HIV prevalence measures. After multiple level screening, 192 eligible reports were included in the review. There were 197 HIV prevalence measures on a total of 58,241 PWID extracted from reports, and an additional 226 HIV prevalence measures extracted from the databases.

We estimated that there are 626,000 PWID in MENA (range: 335,000–1,635,000, prevalence of 0.24 per 100 adults). We found evidence of HIV epidemics among PWID in at least one-third of MENA countries, most of which are emerging concentrated epidemics and with HIV prevalence overall in the range of 10%–15%. Some of the epidemics have however already reached considerable levels including some of the highest HIV prevalence among PWID globally (87.1% in Tripoli, Libya). The relatively high prevalence of sharing needles/syringes (18%–28% in the last injection), the low levels of condom use (20%–54% ever condom use), the high levels of having sex with sex workers and of men having sex with men (15%–30% and 2%–10% in the last year, respectively), and of selling sex (5%–29% in the last year), indicate a high injecting and sexual risk environment. The prevalence of HCV (31%–64%) and of sexually transmitted infections suggest high levels of risk behavior indicative of the potential for more and larger HIV epidemics.

**Conclusions:**

Our study identified a large volume of HIV-related biological and behavioral data among PWID in the MENA region. The coverage and quality of the data varied between countries. There is robust evidence for HIV epidemics among PWID in multiple countries, most of which have emerged within the last decade and continue to grow. The lack of sufficient evidence in some MENA countries does not preclude the possibility of hidden epidemics among PWID in these settings. With the HIV epidemic among PWID in overall a relatively early phase, there is a window of opportunity for prevention that should not be missed through the provision of comprehensive programs, including scale-up of harm reduction services and expansion of surveillance systems.

*Please see later in the article for the Editors' Summary*

## Abstract 2

Laith Abu-Raddad and colleagues assess the current state of knowledge of the HIV epidemic among people who inject drugs in the Middle East and North Africa.

*Please see later in the article for the Editors' Summary*

## Editors' Summary

**Background:**

About 35 million people worldwide are currently infected with HIV, the virus that causes AIDS, and around 2.3 million people become newly infected every year. HIV is mainly transmitted through unprotected sex with an infected partner. However, people who inject drugs (PWID) have a particularly high risk of HIV infection because blood transfer through needle and syringe sharing can transmit the virus. Worldwide, 5%–10% of all HIV-positive people are PWID but in some regions of the world the fraction of all HIV-positive people that are PWID is even higher. To meet the global health challenge of the high HIV prevalence (the proportion of a population that has a specific disease) among PWID, the Joint United Nations Programme on HIV/AIDS (UNAIDS) and other international bodies endorse harm reduction strategies to prevent risky injection behaviors among PWID. These strategies include education and the provision of clean needles, syringes, and opioid substitution therapy.

**Why Was This Study Done?:**

To maximize the effect of these harm-reduction strategies in specific regions, it is important to understand the status of the HIV epidemic among PWID. Although surveillance systems provide the information on HIV infection needed to track the progress of HIV epidemics among PWID in many regions, little is known about the HIV epidemic among PWID in the Middle East and North Africa (MENA, a geographical region that encompasses countries that share historical, socio-cultural, linguistic, and religious characteristics). Several factors contribute to the likelihood of individuals injecting drugs in MENA. For example, Afghanistan (a MENA country) produces most of the world's supply of heroin, which is largely trafficked through Iran and Pakistan (also MENA countries). In this systematic review and data synthesis, the researchers use predefined criteria to identify all the published and unpublished data on HIV prevalence and incidence (the number of new cases of a disease in a population in a given time) among PWID in MENA and combine (synthesize) these data to assess the status of the HIV epidemic in this key population for HIV transmission in MENA.

**What Did the Researchers Do and Find?:**

The researchers identified 192 reports that reported the prevalence/incidence of HIV, other sexually transmitted infections and infection with hepatitis C virus (HCV, another virus transmitted through drug injection) among PWID, the prevalence of injecting or sexual risk behaviors among PWID, or the number/proportion of PWID in MENA. From these data, the researchers estimated that there are about 600,000 PWID in MENA (a prevalence of 0.24 per 100 adults, which is comparable with figures from other regions). The data provided evidence for HIV epidemics among PWID in at least a third of MENA countries, mainly emerging concentrated epidemics (epidemics that are still growing but in which HIV infection and transmission are already considerable). HIV prevalence among PWID in MENA varied considerably, reaching an extremely high prevalence of 87.1% in Tripoli, Libya. The data also revealed a high injecting and sexual risk environment among PWID in MENA (for example, on average, about a quarter of PWID shared a needle or syringe in their most recent injection and only a third reported ever using condoms) that, together with a high prevalence of HCV and sexually transmitted infections among PWID, indicates the potential for more and larger HIV epidemics.

**What Do These Findings Mean?:**

These findings indicate that substantial amounts of HIV-related data have been collected from PWID in MENA but that the coverage and quality of these data vary widely between countries. They provide robust evidence for growing HIV epidemics, most of which have emerged within the past decade, among PWID in several MENA countries, but do not preclude the possibility of hidden epidemics among PWID in additional MENA countries. Overall, these findings suggest that the HIV epidemic among PWID in MENA is at a relatively early stage. This window of opportunity to control the emerging epidemics should not be missed, warn the researchers. HIV surveillance among PWID in MENA must be expanded to detect and monitor emerging and growing HIV epidemics, they suggest, and to inform effective HIV policy and programming. Improvements in HIV prevention and treatment among PWID in MENA are essential, they conclude, to confront the growing HIV problem in this population and, to prevent the onward transmission of HIV from PWID to other population groups.

**Additional Information:**

Please access these websites via the online version of this summary at http://dx.doi.org/10.1371/journal.pmed.1001663.

- A 2010 report produced by the World Bank, UNAIDS, and WHO provides information on the status of the HIV epidemic in the Middle East and North Africa; the UNAIDS Middle East and North Africa Regional Report on AIDS 2011 provides further information
- The 2013 UNAIDS World AIDS Day Report provides up-to-date information about the AIDS epidemic and efforts to halt it
- The Middle East and North Africa Harm Reduction Association (MENAHRA) provides information about harm reduction efforts, services, and programs in the Middle East and North Africa; Harm Reduction International provides information about harm reduction concepts, strategies, programs, and publications globally
- Information is available from the US National Institute of Allergy and Infectious Diseases on HIV infection and AIDS
- NAM/aidsmap provides basic information about HIV/AIDS, and summaries of recent research findings on HIV care and treatment
- Information is available from Avert, an international AIDS charity, on many aspects of HIV/AIDS, including information on people who inject drugs and HIV/AIDS and on harm reduction and HIV prevention (in English and Spanish)
- The US National Institute on Drug Abuse also provides information about drug abuse and HIV/AIDS (in English and Spanish)
- Personal stories about living with HIV/AIDS are available through Avert, Nam/aidsmap, and Healthtalkonline

## Introduction

The Middle East and North Africa (MENA) region has been singled out as the region with little data and where the status of the HIV/AIDS epidemic remained unknown [1]–[8]. In 2005, the region was characterized as “a real hole in terms of HIV/AIDS epidemiological data” [9]. The MENA region has, however, witnessed a remarkable growth in HIV research over the last decade, with several countries developing surveillance systems to monitor the spread of HIV infection, including among most-at-risk populations [10].

A large fraction of studies conducted in the region has remained unpublished in the scientific literature, and only available in the form of difficult to access country reports. This has meant that data have not been analyzed or synthesized at either country or regional level, and no critical assessment of the quality of available evidence has been conducted. The rationale for this study came from signs of a growing HIV disease burden in the MENA region, which highlighted the urgent need for a critical and comprehensive evaluation of the status of the HIV epidemic and of the quality of evidence among the different population groups to inform HIV policy and programming in the region; this was the mandate of the MENA HIV/AIDS Synthesis Project, the largest HIV study in MENA to date [11].

The present article follows on from a series of studies conducted as part of the Synthesis Project. These studies include a high-level overview of HIV epidemiology in MENA [12], a systematic review of HIV molecular evidence [13], and the first documentation of the emerging HIV epidemic among men who have sex with men (MSM) in MENA [14]. The present study is, to our knowledge, the first systematic review and data synthesis to characterize the status of the HIV epidemic among people who inject drugs (PWID) in MENA. The presented regional analysis takes on an additional importance with the need to capture the volume of bio-behavioral surveillance data that became available within the last few years in MENA, and is yet to be analyzed and synthesized within a country-specific or a regional context [15].

PWID are one of the key populations at high risk of HIV in MENA, a region with several vulnerability factors for injecting drug use. For example, 83% of the global supply of heroin is produced in Afghanistan [16], and over 75% of this is trafficked through Iran and Pakistan. In 2009, Iran bore the highest fraction of the global opium and heroin seizures (89% and 33%, respectively) [16]. Increased availability and purity of heroin at lower prices in MENA appears to have led to a subsequent rise in injecting drug use [17]. In 2010, one gram of heroin in Afghanistan could be purchased for about US$4 compared with up to US$100 in West and Central Europe, US$200 in the United States and Northern Europe, and US$370 in Australia [16]. Most PWID in the region are young adults and marginalized by family members and society; they are stigmatized and lack access to comprehensive and confidential HIV prevention and treatment services [11].

The primary objective of this study was to assess the status of the HIV epidemic among PWID in MENA by describing HIV prevalence and incidence. The secondary objective was to describe the risk behavior environment and the HIV epidemic potential among PWID by describing (1) their injecting and sexual risk behavior and knowledge, and (2) prevalence of proxy biological markers of these behaviors, namely hepatitis C virus (HCV) and sexually transmitted infections (STIs), respectively. The study also estimated the proportion and number of PWID in MENA.

## Methods

We followed the Preferred Reporting Items for Systematic Reviews and Meta-Analyses (PRISMA) (Text S1) [18],[19] and Cochrane Collaboration guidelines [20].

### Data Sources and Search Strategy

**Figure 1:**
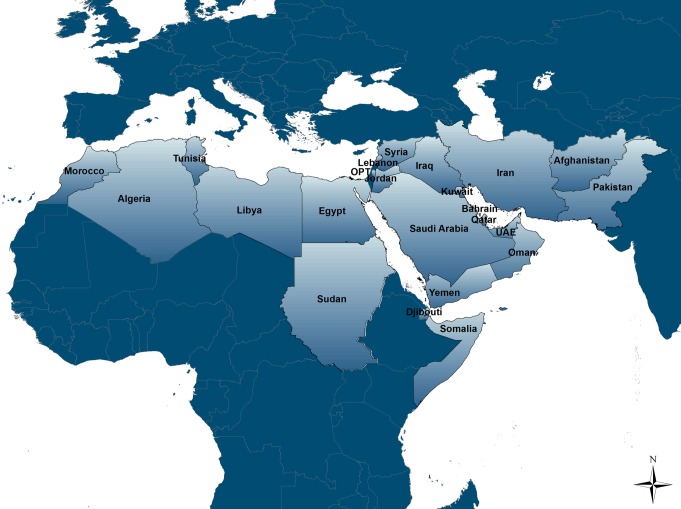
Map of the Middle East and North Africa region. The defintion adopted in the review includes the following 23 countires: Afghanistan, Algeria, Bahrain, Djibouti, Egypt, Iran, Iraq, Jordan, Kuwait, Lebanon, Libya, Morocco, Oman, OPT, Pakistan, Qatar, Saudi Arabia, Somalia, Sudan (including the newly established Republic of South Sudan), Syria, Tunisia, United Arab Emirates (UAE), and Yemen.

Our review covered the 23 countries included in the MENA definitions of the three international organizations leading the regional HIV response efforts in the region: the Joint United Nations Programme on HIV/AIDS (UNAIDS), the Eastern Mediterranean Regional Office of the World Health Organization (WHO/EMRO), and the World Bank (Figure 1). These countries share specific similarities, whether historical, socio-cultural, or linguistic; and are conventionally included together as part of HIV/AIDS programming for the region.

The following sources of data were searched up to December 16, 2013: (1) Scientific databases (PubMed, Embase, and regional databases [WHO African Index Medicus [21] and WHO Index Medicus for the Eastern Mediterranean Region [22]]), with no publication date or language restrictions. A generic search of “drug use” in MENA was performed in PubMed and Embase using MeSH/Emtree and text terms. The term “HIV” was not included to avoid detection bias. (2) The MENA HIV/AIDS Synthesis Project database of grey and mainly unpublished literature [11],[12]. (3) Abstracts of the International AIDS Conference 2002–2012 [23], the International AIDS Society Conference on HIV Pathogenesis and Treatment 2001–2013 [24], and the International Society for Sexually Transmitted Diseases Research Conferences 2003–2013 [25]. (4) International and regional databases of HIV prevalence measures including the US Census Bureau database of HIV/AIDS [26], the WHO/EMRO HIV testing database [27], and the UNAIDS epidemiological fact sheets database [28].

Details of the search criteria are provided in Text S2. Reference lists of all relevant papers and review articles were also searched.

### Study Selection

Titles and abstracts of all records identified were screened independently by two authors (GRM and SR), and consensus on potential eligibility reached. Full texts of potentially relevant records were retrieved and assessed for eligibility. Studies satisfying any of the below criteria were eligible: (1) The proportion of PWID in the sample was specified, at least half were PWID, and data on any of the following outcomes were included: Prevalence or incidence of HIV; prevalence of injecting or sexual risk behaviors, or knowledge; prevalence or incidence of HCV; and prevalence or incidence of other STIs. HCV is transmitted primarily through percutaneous exposures and can be used as a proxy of the risk of parenteral exposure to HIV. Among PWID, a threshold HCV prevalence of about 30% implies sufficient risk behavior to sustain HIV transmission [29],[30]. Similarly, the prevalence of STIs is a useful marker of sexual risk behavior and potential for HIV sexual acquisition. (2) Data on population-based prevalence of injecting drug use or PWID population size estimates were reported.

Only studies with primary data were included. The only exception was in relation to national estimates of the number and proportion of PWID in a number of MENA countries where the only available source of data was from two global reviews [4],[31] that published data compiled through the Reference Group to the UN on HIV and Injecting Drug Use [32].

We used the term report to refer to the documents (papers, conference abstracts, or public health reports) presenting findings of a study [20]. Reports could contribute to more than one outcome. Findings duplicated in more than one report were included only once (using the more detailed report). Outcomes in more than one population/setting within a report were included separately.

### Data Extraction

Data were extracted by one of the authors (GRM) using a pre-piloted data extraction form and entered into a computerized database. Double extraction on about 45% of records was confirmed by another author (LA-R). The few discrepancies were settled by consensus or by contacting authors. Data from articles in English, French, and Arabic were extracted from the full -texts. Data from records in Farsi (*n* = 6) were extracted from the English abstract. There were no records in other languages.

As supporting information, we also analyzed data extracted from countries' reporting on the HIV epidemic to WHO/EMRO in the format of aggregate HIV case notifications.

### Scope and Quality of the Evidence

We appraised the status of the evidence on our main outcome, HIV prevalence, at country level by examining the following criteria that take into consideration the quantity, quality, and geographical coverage of available data: (1) the number of HIV prevalence measures and the total sample size they cover, (2) the number of geographic settings with HIV prevalence measures, (3) the number of multi-city studies and the maximum number of cities per study, (4) the number of rounds of integrated bio-behavioral surveillance surveys (IBBSS), and (5) the quality and precision of individual HIV prevalence measures.

The quality of individual HIV prevalence measures was assessed by describing the risk of bias (ROB). Since the number of prevalence measures among female PWID was very small and often based on small sub-samples, the quality appraisal was restricted to HIV prevalence among predominantly male PWID. Based on the Cochrane approach for assessing ROB [20], we classified each HIV prevalence measure as having a low, high, or unclear ROB for three quality domains: the sampling methodology, the type of HIV ascertainment, and the response rate. Low ROB was considered if (1) sampling was probability-based or preceded by ethnographic mapping, (2) HIV was ascertained with a biological assay, and (3) the response rate was over 80%; or over 80% of the target sample size was reached. HIV prevalence measures extracted from international and regional databases were considered of unknown quality since original reports were not available for assessing their ROB.

A minimum sample size of 100 was considered to produce estimates with good precision. For a median HIV prevalence among PWID in MENA of 8% (see Results), this implies a 95% CI of 4%–15%.

The quality of the evidence in each country was assessed by combining the above factors as described in Text S3. For example, quality was considered better if at least one round of IBBSS was conducted, since these surveys use standard methodology including state of the art sampling techniques of hard-to-reach populations (such as respondent-driven sampling). Countries were categorized as having: (1) No evidence: virtually no data. (2) Poor evidence: The majority of HIV biological measures were of poor quality. (3) Limited evidence: The number of HIV biological measures was small, but of reasonable quality. (4) Good evidence: The number of HIV biological measures was small, but with good quality and informative data. However, the overall volume of data was not sufficient to be conclusive of the status and scale of the epidemic at the national level. (5) Conclusive evidence: There was a sufficient volume of robust evidence to support the conclusion.

### Analysis

The low-bound, middle, and high-bound national estimates of the number and prevalence of injecting drug use in MENA countries were extracted from reports. The pooled number and prevalence of PWID for the MENA region were estimated separately using the extracted country-level estimates. The lower (and upper) bound of our pooled regional estimate of the number of PWID in MENA was calculated by adding the lowest (and highest) reported number of PWID in all MENA countries. The middle figure for the number of PWID in MENA is the sum of the middle estimates in each of the MENA countries. When more than one such estimate was available per country, we used the median of the estimates. The pooled numbers of PWID were rounded up to the next thousand.

Middle estimates of the extracted prevalence of PWID were weighted by adult population size to derive the pooled prevalence of injecting drug use in MENA. When more than one such estimate was available per country, we used the median of the estimates. Adult population size was extracted from the United Nations World Population Database [33]. Sub-national estimates of the number and prevalence of injecting drug use were extracted from reports and described separately.

We calculated 95% CI for HIV and HCV prevalence for all reports with available information. The HIV biological data (HIV prevalence from reports and from databases, HIV incidence, and notified HIV cases) were synthesized at country level to assess the status of the HIV epidemic among PWID. Recent WHO/UNAIDS guidelines for classifying HIV epidemics [34],[35], which do not recommend use of rigid thresholds [34],[36], were adapted to classify the HIV epidemic level in PWID as: (1) Low-level HIV epidemic: HIV has not reached significant levels among PWID. (2) Concentrated HIV epidemic: HIV has reached significant levels and taken root among PWID through transmission chains between members of this population. Concentrated epidemics can be either emerging (HIV has started its initial growth and continues in a trend of increasing HIV prevalence); or established (the epidemic has reached its peak and HIV prevalence is stabilizing towards, or already is at, its endemic level). (3) “At least outbreak-type”: Insufficient evidence to support a concentrated epidemic among PWID, but some evidence, usually of lower quality, suggesting that significant HIV transmission has occurred, or is occurring, among at least some PWID groups.

The terms “national” or “at least localized” were assigned to concentrated epidemics to reflect the geographical spread of the epidemic within a given country.

## Results

### Results of Search Strategy

**Figure 2:**
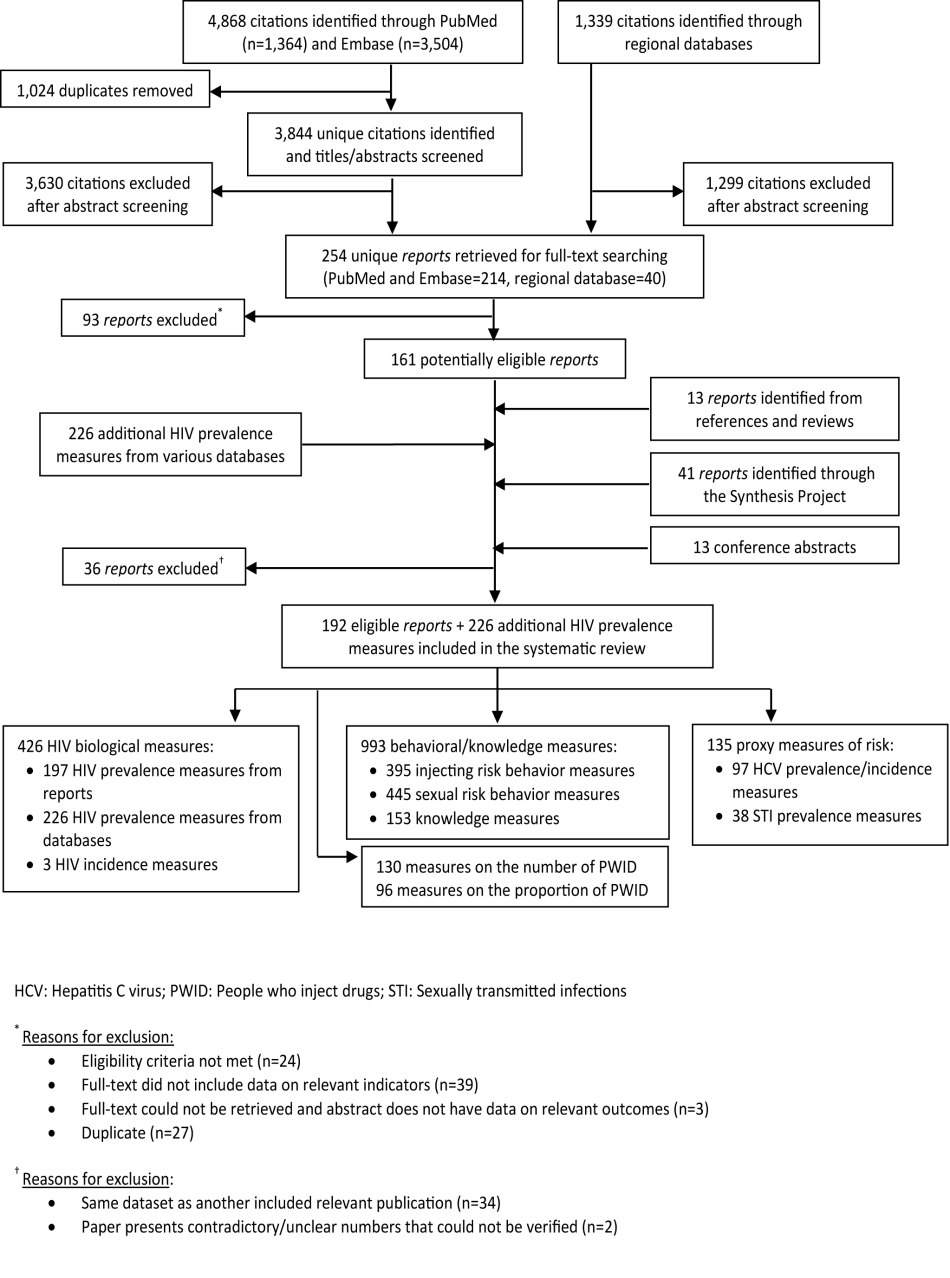
PRISMA flow chart of study selection in the systematic search.

The study selection process is shown in Figure 2. A total of 6,207 citations were retrieved from PubMed, Embase, and the regional databases. After full-text screening and including reports from the other sources, 192 reports were eligible: 121 from PubMed and Embase, 41 from the MENA HIV/AIDS Synthesis Project, 13 from bibliographies of relevant reports and review articles, 13 from the search of scientific conferences, and four from the regional databases. In addition, 226 HIV point-prevalence measures were extracted from the databases of biological markers (Figure 2).

There were 423 HIV prevalence measures, 197 of which were extracted from the eligible reports and 226 from the databases of HIV prevalence; three HIV incidence measures; 93 HCV prevalence measures; four HCV incidence measures; 38 STI prevalence measures; and 993 behavioral and knowledge measures. There were also 130 and 96 measures on the number and proportion of PWID, respectively (Figure 2).

### Scope and Quality of the Evidence

The number and quality of HIV prevalence measures varied by country. The largest volume of data was from Pakistan (101 HIV prevalence measures on a total of 24,445 PWID), Iran (99 HIV prevalence measures on a total of 22,181 PWID), and Egypt (39 HIV prevalence measures on a total of 4,480 PWID) (Table 1). A smaller number of HIV prevalence measures but covering a relatively large number of PWID were conducted in Afghanistan (3,277 PWID), Tunisia (1,522 PWID), and Morocco (880 PWID). Multi-city studies have been conducted in several countries including Pakistan, where up to 16 cities were included in one study [37]. IBBSS have been conducted in Afghanistan [38],[39], Egypt [40]–[42], Iran [43],[44], Jordan [45], Lebanon [46], Libya [47], Morocco [48], Occupied Palestinian Territories (OPT) [49], Pakistan [37],[50]–[52], and Tunisia (Table 1) [53],[54]. Pakistan has the most repeated rounds of IBBSS with four rounds conducted between 2005 and 2011 [37],[50]–[52].

**Table 1:** Summary of the HIV biological evidence per country.

Biological Evidence	Afg	Alg	Bah	Dji	Egy	Irn	Irq	Jor	Kuw	Leb	Lib	Mor	Oma	OPT	Pak	Qat	SA	Som	Sud	Syr	Tun	UAE	Yem
**Number HIV biological studies** a	5	—	1	—	7	47	—	1	—	2	1	3	1	1	27	—	—	—	—	1	2	—	—
**Number HIV prevalence measures**	19	—	23	6	39	99	7	13	17	8	4	21	17	4	101	—	7	—	1	22	10	—	5
**From reports (total sample size)**	13 (3,277)	—	1 (242)	—	9 (4,480)	78 (22,181)	—	3 (227)	—	2 (121)	1 (328)	5 (880)	3 (135)	1 (199)	*77* (24,445)	—	—	—	—	1 (204)	3 (1,522)	—	—
**From databases**	6	—	22	6	30	21	7	10	17	6	3	16	14	3	*24*	—	7	—	1	21	7	—	5
**Number HIV incidence measures** a	1	—		—	—	1	—	—	—	—	—	—	—		1	—	—	—	—	—	—	—	—
**Number cities/provinces with HIV prevalence measures** a	6	—	1	—	2	27	—	3	—	1	1	4	1	1	26	—	—	—	—	1	3	—	—
**Number multi-city studies (max number cities/study)** a	3 (5)	—	—	—	1 (2)	4 (10)	—	1 (4)	—	—	—	2 (2)	—	—	12 (16)	—	—	—	—	—	2 (3)	—	—
**Number repeated IBBSS** a	2	—	—	—	2	2	—	1	—	1	1	1	—	1	4	—	—	—	—	—	2	—	—

a With reports available.

Afg, Afghanistan; Alg, Algeria; Bah, Bahrain; Dji, Djibouti; Egy, Egypt; Irn, Iran; Irq, Iraq; Jor, Jordan; Kuw, Kuwait; Leb, Lebanon; Lib, Libya; Mor, Morocco; Oma, Oman; Pak, Pakistan; QA, Qatar; SA, Saudi Arabia; Som, Somalia; Sud, Sudan; Syr, Syria; Tun, Tunisia; UAE, United Arab Emirates; Yem, Yemen.

Of 190 HIV prevalence measures extracted from eligible reports and among predominantly male PWID, 98%, 53%, and 34% had low ROB in terms of HIV ascertainment, sampling methodology, and response rate, respectively. Over 60% of the 190 HIV prevalence measures had low ROB in at least two quality domains and 84% had good precision (Tables S1 and S2).

On the basis of the quality of the evidence assessment, the evidence was determined to be “conclusive” in Iran and Pakistan; “good” in Afghanistan, Egypt, Jordan, Lebanon, Libya, Morocco, OPT, and Tunisia; “limited” in Bahrain and Syria; and “poor” in Djibouti, Iraq, Kuwait, Oman, Saudi Arabia, Sudan, and Yemen. There was “no evidence” in Algeria, Qatar, Somalia, and the United Arab Emirates. A narrative justification for the classification of the scope and quality of evidence is in Text S3.

Although a formal quality assessment was not made for the secondary outcomes in terms of injecting and sexual risk behavior and knowledge, the majority of these data were extracted from the IBBSS studies using standard survey methodology and large samples. Details of these studies (with information on sample size, population characteristics, and/or sampling technique) can be found in the tables summarizing the prevalence of HIV and HCV among PWID (Tables 3 and 6).

### Prevalence of Injecting Drug Use

Table 2 describes national estimates of the number and prevalence of PWID. These national estimates were extracted from included reports where they were derived using different methodologies including indirect methods (such as capture-recapture and multiplier methods), population-based surveys, registered number of PWID, and rapid assessments. In two of the sources of data in Table 2 [4],[31], some of the country estimates are the collation of several such country-specific estimates using methods described in the original reports [4],[31].

**Table 2:** National estimates of the number and prevalence of people who inject drugs in the Middle East and North Africa as extracted from included reports.

Country	Population 15–64 Years [33]	Year of Estimate	PWID Estimate (Number)			PWID Prevalence (%)			Source
			Low	Middle	High	Low	Middle	High	
**Afghanistan**	16,119,000	a	22,720	34,080	45,440	0.16	0.24	0.32	[4]
		2005	6,870	6,900	6,930	0.05	0.05	0.05	[31]
		2009	18,000	20,000	23,000				[124]
		2009					0.11		[125]
**Algeria**	24,246,000	a	26,333	40,961	55,590	0.14	0.22	0.29	[4]
**Bahrain**	983,000	a	337	674	1,011	0.08	0.16	0.24	[4]
**Djibouti**		—	—	—	—	—	—	—	—
**Egypt**	51,460,000	a	56,970	88,618	120,265	0.13	0.21	0.28	[4]
**Iran**	53,132,000	a	70,000	185,000	300,000	0.17	0.46	0.74	[4]
		2004		180,000			0.40		[31]
		2007		250,000					[126]
**Iraq**	16,967,000	a	23,115	34,673	46,230	0.19	0.28	0.37	[4]
**Jordan**	3,624,000	a	3,200	4,850	6,500	0.11	0.16	0.22	[4]
**Kuwait**	1,937,000	a	2,700	4,100	5,500	0.20	0.30	0.41	[4]
**Lebanon**	2,871,000	a	2,200	3,300	4,400	0.09	0.14	0.19	[4]
**Libya**	4,148,000	a	4,633	7,206	9,779	0.15	0.23	0.32	[4]
		2001		1,685			0.05		[31]
**Morocco**	21,247,000	a		18,500			0.10		[4]
**Oman**	1,956,000	a	2,800	4,250	5,700	0.20	0.30	0.40	[4]
**OPT**	2,212,000	a	1,200	1,850	2,500	0.22	0.35	0.47	[4]
**Pakistan**	104,724,000	a	54,000	462,000	870,000	0.07	0.50	1.12	[4]
		2006	125,000	130,460	150,000	0.13	0.14	0.16	[31]
		2006		102,042			0.25		[57]
		2010		99,000					[126]
**Qatar**	1,503,000	a	780	1,190	1,600	0.15	0.22	0.30	[4]
**Saudi Arabia**	18,306,000	a	15,172	23,600	32,028	0.13	0.20	0.27	[4]
		2008		10,000					[126]
**Somalia**	4,885,000	a		1,000			0.03		[4]
**Sudan**	24,540,000	a	24,319	37,828	51,337	0.13	0.20	0.28	[4]
**Syria**	12,073,000	a	4,000	6,000	8,000	0.04	0.07	0.09	[4]
**Tunisia**	7,294,000	a	8,462	13,163	17,864	0.14	0.21	0.29	[4]
		2009		9,000					[126]
**UAE**	6,200,000	a	3,200	4,800	6,400	0.20	0.30	0.40	[4]
**Yemen**	12,800,000	a	12,710	19,770	26,830	0.15	0.23	0.31	[4]

a The specific year of the estimate was not mentioned in the original report, but the report covered data from 1998–2005.

UAE, United Arab Emirates.

Based on available data, the number of PWID in MENA ranges between a low bound of 335,000 and a high bound of 1,635,000, with a middle estimate of 626,000 PWID. Iran, Pakistan, and Egypt have the largest number, with a median of about 185,000, 117,000, and 89,000 PWID, respectively. The weighted mean prevalence of injecting drug use in MENA is 0.24 per 100 adults. It is lowest in Somalia (0.03%) and highest in Iran (0.43%) (Table 2).

Studies of sub-national populations showed geographical heterogeneity (Table S3). For example, in Iran, the prevalence of injecting drug use varied between 0.0% in rural Babol province [55] to 1.0% in Tehran [56]; and in Pakistan it ranged from 0.02% in Rawalpindi to 0.87% and 1.07% in Sargodha and Faisalabad, respectively [57].

Data on the prevalence of female PWID in MENA were scarce. Overall, the mean proportion of females among PWID in included studies was 2.3% (range: 0%–35%). In two studies in Oman and Syria, 25%–58% [58] and 48% [59] of PWID, respectively, reported knowing at least one female PWID.

### HIV Prevalence, Incidence, and Mode of Transmission

HIV prevalence measures from reports and databases are summarized in Tables 3 and S4, respectively. Considerable variation in HIV prevalence was seen, with an overall median of 8% (interquartile range [IQR]: 1%–21%) (Table 3). HIV prevalence among PWID in MENA ranged between 0% in some prevalence measures in almost every country up to 7% in Cairo, Egypt in 2010 (*n* = 274) [42]; 18% in Afghanistan in one city near the Iranian borders, Herat, in 2009 (*n* = 159) [38]; 21% in Manama, Bahrain, in the early nineties (*n* = 242) [60]; 27% in Oman among incarcerated PWID (*n* = 33) [58]; 38% in Nador, northern Morocco, in 2008 (*n* = 233) [61]; 52% in the third largest metropolis in Pakistan, Faisalabad, in 2011 (*n* = 364) [37]; 72% in rural Iran in 2004–5 (*n* = 61) [62]; and 87% in Tripoli, Libya in 2010 (*n* = 328) [47] (Table 3). HIV prevalence was consistently low among PWID in Jordan, Lebanon, OPT, Syria, and Tunisia (0%–3.1%). Substantial intra-country variability in HIV prevalence was observed in Afghanistan, Iran, Morocco, and Pakistan (Table 3). In most countries with high HIV prevalence, recent studies report increasing HIV prevalence starting around 2003 (Tables 3 and S4).

**Table 3:** HIV prevalence among people who inject drugs in the Middle East and North Africa as extracted from reports included in the systematic review.

Country	Citation	Year	City	Study Site	Sampling	Population	Sample Size	HIV Prevalence	
								Percent	95% CI
**Afghanistan**	MOH, 2012 [39] (Round II)	2012	Herat		RDS	All male	185	13.3^a^	8.9–19.3
			Kabul		RDS	All male	369	2.4^a^	1.1–4.6
			Mazar-i-Sharif		RDS	All male	254	0.3^a^	0.0–2.2
			Jalalabad		RDS	All male	236	1.0^a^	0.1–3.0
			Charikar		RDS	All male	117	0.9^a^	0.0–4.7
	MOH, 2010 [38] (Round I)	2009	Herat		RDS	All male	159	18.2	12.6–25.1
			Kabul		RDS	All male	286	3.2	1.4–5.9
			Mazar-i-Sharif		RDS	All male	102	1.0	0.0–5.3
	Todd, 2011 [69]	2007–2009	Kabul	Harm reduction center & community	CS	All male	483	2.1	1.0–3.8
	Nasir, 2011 [127]	2006–2008	Herat	VCT	CS	99% male	340	3.2	1.6–5.7
			Jalalabad	VCT	CS	99% male	96	0.0	—
			Mazar-i-Sharif	VCT	CS	99% male	187	0.0	—
	Todd, 2007 [128]	2005–2006	Kabul	VCT	CS	All male	463	3.0	1.7–5.0
**Bahrain**	Al-Haddad, 1994 [60]	1991	Manama	Voluntary drug treatment center	CS	All male	242	21.1	16.1–26.8
**Egypt**	MOH/FHI, 2010 [42] (Round II)	2010	Alexandria		RDS	All male	284	6.5^a^	3.3–10.3^a^
			Cairo		RDS	All male	274	6.8^a^	3.9–10.8^a^
	Elghamrawy, 2012 [129]	2008–2011	Cairo	Harm reduction center	CS	All male	3,222	1.4	1.0–1.9
	Soliman, 2010 [41] (Round I)	2006	Cairo		RDS	All male	413	0.6^a^	0.1–1.8^a^
	MOH/FHI, 2006 [40] (Round I)	2006	Cairo		RDS	All female	16	0.0	—
	Saleh, 1998 [130]	1994	Alexandria	Voluntary drug treatment center	CS		100	0.0	—
	Attia, 1996 [131]	—	Alexandria	Voluntary drug treatment center	CS		54	0.0	—
	Hasan, 1994 [132]	—			CS		79	7.6	2.8–15.8
	El-Ghazzawi, 1987 [133]	—	Alexandria		CS		38	0.0	—
**Iran**	Honarvar, 2013 [134]	2012–2013	Shiraz	Voluntary drug treatment center	CS	98% male	233	7.7	4.6–11.9
	Mehrejredi, 2013 [135]	2011	Tehran	VCT and harm reduction center	CS	91% male	209	2.9	1.1–6.1
	MOH, 2010 [44] (Round II)	2010	Fars	VCT, Harm reduction center, voluntary drug treatment center, & community	CS	98% male	250	31.9	26.3–38.2
			Lorestan	Idem	CS	All male	222	26.4	20.9–32.9
			Tehran	Idem	CS	95% male	567	23.9	20.5–27.7
			Sistan & Baluchestan	Idem	CS	99% male	138	18.3	12.1–25.6
			Kermanshah	Idem	CS	99% male	249	16.8	12.4–22.1
			Khouzestan	Idem	CS	99% male	198	9.4	5.9–14.6
			Mazandaran	Idem	CS	97% male	276	7.0	4.2–10.5
			Kerman	Idem	CS	94% male	213	6.2	3.3–10.2
			Azerbaijan Sharghi	Idem	CS	100% male	118	3.6	0.9–8.5
			Khorasan Razavi	Idem	CS	99% male	248	2.2	0.7–4.6
	Alipour, 2012 [79]	2010	Tehran, Shiraz, & mashhad	Harm reduction center	CS	All male, heterosexually active	226	9.4	5.8–13.9
			Tehran, Shiraz, & mashhad	Harm reduction center	CS	All female, sexual partners of PWID	42	7.7	1.5–19.5
	Ilami, 2010 [136]	2009–2010	Kohgiloyeh & Boyerahmad		CS		158	9.9	5.9–15.9
	Hashemepour, 2013 [137]	2009	North Isfahan	Community	CS		82	1.2	0.0–6.6
			South Isfahan	Community	CS		589	1.0	0.4–2.2
			West Isfahan	Community	CS		479	1.7	0.7–3.3
			East Isfahan	Community	CS		113	3.5	1.0–8.8
			Isfahan city	Community	CS		336	1.5	0.5–3.4
	Dibaj, 2013 [138]	2008–2009	Isfahan	Prison	CS	All male	970	6.4	4.9–8.1
	Javadi, 2013 [139]	2008–2009	Isfahan	Harm reduction center	CS	95% male	539	1.1	0.4–2.4
	Eskandarieh, 2013 [140]	2008	Tehran	Mandatory drug treatment center	CS	97% male	258	18.8	14.4–24.3
	Zamani, 2010 [141]	2008	Isfahan		RDS	98% male	117	0.7^a^	0·6–2.3^a^
	Ghasemian, 2011 [142]	2007–2009	Sari	Clinical setting	CS		88	18.2	10.8–27.8
	Zadeh, 2014 [143]	2007–2008	Tehran	Prison	CS		3,044	3.7	3.1–4.4
	SeyedAlinaghi, 2013 [144]	2007–2008	Tehran	Community	CS	Beggars	658	2.4	1.4–3.9
	Kazerooni, 2010 [67]	2007	Shiraz	Prison	SRS	All male	363	6.6	4.3–9.7
	Aminzadeh, 2007 [145]	2007	Tehran	Clinical setting	CS		70	30.0	19.6–42.1
	Rahimi_Movaghar, 2010 [146]	2006–2007	Tehran	Voluntary drug treatment center & community	CS	All female	38	10.5	2.9–24.8
			Tehran	Voluntary drug treatment center & community	CS	All male	861	10.7	8.7–12.9
	Kheirandish, 2010 [147]	2006	Tehran	Mandatory drug treatment center	CS	All male	459	24.4	20.5–28.6
	MOH, 2008 [43] (Round I)	2006–2007	Azerbaijan Sharghi	Harm reduction center, voluntary drug treatment center, & community	TLS	96% male	294	8.2	5.3–11.9
			Fars	Idem	TLS	92% male	353	24.7	20.2–29.5
			Kerman	Idem	TLS	96% male	162	20.8	15.0–28.1
			Kermanshah	Idem	TLS	99% male	259	30.5	25.0–36.5
			Khorasan Razavi	Idem	TLS	98% male	399	6.5	4.3–9.4
			Khuzestan	Idem	TLS	99% male	168	4.2	1.7–8.4
			Lorestan	Idem	TLS	97% male	196	35.7	29.0–42.9
			Mazandaran	Idem	TLS	All male	216	11.6	7.6–16.6
			SIstan	Idem	TLS	93% male	142	2.1	0.4–6.0
			Tehran	Idem	TLS	98% male	664	14.4	11.9–17.4
	Malekinejad, 2008 [148]	2006–2007	Tehran		RDS	98% male	548^a^	25.0	18.0–28.3
	Alavi, 2012 [149]	2005–2006	Ahfaz	Voluntary drug treatment center & prison	CS	All male	109	47.7	38.1–57.5
	Ghanbarzadeh, 2006 [150]	2005	Birjand	Prison	CS	All female	10	0.0	—
	Tofigi, 2011 [151]	2004	Tehran	Clinical setting	CS	Cadavers	400	6.3	4.1–9.1
	Imani, 2008 [152]	2004	Shahr-e-Kord	Voluntary drug treatment center	CS	All male	133	0.8	0·0–4·1
	Mojtahedzadeh, 2008 [62]	2004–2005	Rural Northwestern Iran	Voluntary drug treatment center	CS	98% male, rural population	61	72.1	59.2–82.9
	Zamani, 2006 [102]	2004	Tehran	Harm reduction center & community	CS	All female	6	33.3	4.3–77.7
			Tehran	Harm reduction center & community	CS	All male	207	23.2	17.6–29.5
	Shamaei, 2009 [153]	2003–2006	Tehran	Clinical setting	CS	98% male, TB infected PWID	35	45.7	28.8–63.4
	Pourahmad, 2007 [154]	2003	Isfahan, Chaharmahal Bakhtiary, & Lorestan	Prison	CS	All male	401	14.0	10.7–17.7
	Zamani, 2005 [155]	2003–2004	Tehran	Voluntary drug treatment center	CS	All female	5	20.0	0.5–71.6
			Tehran	Voluntary drug treatment center	CS	All male	165	15.2	10.1–21.5
	Farhoudi, 2003 [156]	2003	Karaj	Resident prisoners	CS	All male, resident inmates	371	24.0	19.7–28.7
			Karaj	Newly admitted prisoners	CS	All male, newly 7–admitted inmates	369	22.0	17.8–26.5
	Khodadadizadeh, 2003 [157]	2003	Rafsanjan	Clinical setting	CS	96% male	31	9.7	2.0–25.8
	Alavi, 2010 [158]	2002–2006	Ahfaz	Clinical setting	CS	97% male, hospitalized for ID	333	18.0	14.6–23.2
	Davoodian, 2009 [159]	2002	Hormozgan	Prison	SRS		249	15.1	11.0–20.3
	Behnaz, 2007 [160]	2002–2003	Gorgan	Prison	SRS		22	18.2	5.2–40.3
	Asadi, 2006 [68]	2002–2004	Tehran	Clinical setting	CS	98% male	126	35.7	27.4–44.7
	Alizadeh, 2005 [161]	2002	Hamadan	Prison	SRS	93% male	149	0.7	0.0–3.7
	Mir Nasseri, 2011 [162]	2001–2002	Tehran	Voluntary drug treatment center	CS	97% male	90	7.8	3·7–13·5
			Tehran	Prison	SRS	87% male	371	17.0	13.5–21.2
	Sharif, 2009 [163]	2001–2006	Kashan	Clinical setting	CS	All female, hospitalized for ID	23	0.0	—
			Kashan	Clinical setting	CS	All male, hospitalized for ID	177	1.6	0.4–4.9
	Alavi, 2009 [164]	2001–2006	Ahfaz	Clinical setting	CS	92% male	142	12.7	7.7–19.3
	Alavi, 2007 [165]	2001–2003	Ahfaz	Clinical setting	CS	All male	154	67.5	59.5–74.8
	Rahbar, 2004 [166]	2001–2002	Mashhad	Voluntary drug treatment center	CS		222	0.0	—
			Mashhad	Prison	CS		101	6.9	2.8–13.8
	Sharifi-Mood, 2006 [167]	2000–2005	Zahedan	Clinical setting	CS	97% male, hospitalized for ID	31	25.8	11.9–44.6
	Mirahmadizadeh, 2004 [168]	1998	Shiraz	Voluntary drug treatment center	CS		464	1.2	0.5–2.8
	Nowroozi, 1998 [169]	1996	Tehran	Prison	SRS	All male	400	0.0	—
	Alavian, 2013 [170]	—	Shiraz	Voluntary drug treatment center	CS	98% male	144	41.7	33.5–50.2
	Azarkar, 2010 [171]	—	Birjand	Prison	SRS		17	0.0	—
	Mirahmadizadeh, 2009 [172]	—	National	National	RCS	96% male	936	20.5	18.0–23.2
	Amini, 2005 [173]	—	Tehran	Voluntary drug treatment center	CS		34	8.8	1.9–23.7
	Alaei, 2002 [174]	—	Kermanshah		CS		429	19.2	15.5–23.2
**Jordan**	NAP, 2010 [45] (Round I)	2009	Amman		RDS		133	0.0	—
			Aqaba		RDS		78	0.0	—
			Irbid		RDS		16	0.0	—
**Lebanon**	Mahfoud, 2010 [46] (Round I)	2007–2008	Beirut		RDS	All male	81	0.0	—
	Ramia, 2003 [175]	2000–2002	Beirut	Clinical setting	CS	75% male	40	0.0	—
**Libya**	Mirzoyan, 2013 [47] (Round I)	2010	Tripoli		RDS		328	87.1^a^	81.5–91.9^a^
**Morocco**	MOH, 2012 [117] (Round I)	2011–2002	Nador		RDS	99% male	277	25.1^a^	16.1–35.0
	MOH, 2012 [117] (Round I)	2010–2001	Tanger		RDS	98% male	261	0.4	0.0–2.1
	MOH, 2010 [61]	2008	Al Hoceima		RDS			0.0	—
			Nador		RDS		233	37.8	31.5–44.3
	Elmir, 2002 [176]	1991–1999	National		CS		109	33	24–43
**Oman**	MOH, 2006 [58]	—	Muscat	Voluntary drug treatment center	CS	All male	17	12^b^	2–36
		—	Muscat	Prison	CS	All male	33	27^b^	13–46
		—	Muscat	Community	SBS	All male	85	18^b^	10–27
**OPT**	MOH, 2010 [177] (Round I)	2010	Al Azaria - East Jerusalem		RDS	98.5% male	199	0.0	—
**Pakistan**	NAP, 2011 [37] (Round IV)	2011	D G Khan	Community	MSCS	98.4% male	365	49.6	44.3–54.8
			Faisalabad	Community	MSCS	98.4% male	364	52.5	47.2–57.7
			Gurjat	Community	MSCS	98.4% male	208	46.2	39.2–53.2
			Lahore	Community	MSCS	98.4% male	367	30.8	26.1–35.8
			Multan	Community	MSCS	98.4% male	365	24.9	20.6–29.7
			Pakpattan	Community	MSCS	98.4% male	365	3.3	1.7–5.7
			Rahim Yar Khan	Community	MSCS	98.4% male	214	14.9	10.5–20.4
			Sarghoda	Community	MSCS	98.4% male	365	40.6	35.5–45.8
			Dadu	Community	MSCS	98.4% male	194	16.0	11.1–21.9
			Karachi	Community	MSCS	98.4% male	365	42.2	37.1–47.4
			Larkana	Community	MSCS	98.4% male	365	18.6	14.8–23.0
			Sukkur	Community	MSCS	98.4% male	365	19.2	15.3–23.6
			Haripur	Community	MSCS	98.4% male	65	7.9	2.5–17.0
			Peshawar	Community	MSCS	98.4% male	260	20.0	15.3–25.4
			Quetta	Community	MSCS	98.4% male	365	7.1	4.7–10.3
			Turbat	Community	MSCS	98.4% male	365	21.4	17.3–25.9
	Nai Zindagi, 2009 [66]	2009	Gurjanwala	Community	CS		300	8	5–12
			Mandi Bahauddin	Community	CS		300	52	46–58
			Rawalpindi	Community	CS		300	23	18–28
			Sheikhukupura	Community	CS		300	21	17–26
	Nai Zindagi, 2008 [89]	2008	Faisalabad		CS	All male, married	104	13	8–22
			Lahore		CS	All male, married	103	10	5–17
			Sarghoda		CS	All male, married	252	41	35–47
	NAP, 2008 [50] (Round III)	2008	D G Khan	Community	MSCS	99.8% male	345	18.6	14.6–23.1
			Faisalabad	Community	MSCS	99.8% male	400	12.3	9.2–15.9
			Hyderabad	Community	MSCS	99.8% male	397	30.5	26.0–35.3
			Karachi	Community	MSCS	99.8% male	403	23.1	19.1–27.5
			Lahore	Community	MSCS	99.8% male	401	14.5	11.2–18.3
			Larkana	Community	MSCS	99.8% male	389	28.5	24.1–33.3
			Peshawar	Community	MSCS	99.8% male	231	12.8	8.9–18.0
			Sarghoda	Community	MSCS	99.8% male	403	22.8	18.8–27.2
	Platt, 2009 [178]	2007	Rawalpindi		RDS	98% male	302	2.6	1.2–5.2
			Abotabad		RDS	98% male	102	0.0	—
	NAP, 2006–2007 [51] (Round II)	2006–2007	Bannu	Community	MSCS		72	1.4	0.0–7.5
			Faisalabad	Community	MSCS		400	13.3	10.1–17.0
			Gurjanwala	Community	MSCS		400	1.0	0.3–2.5
			Hyderabad	Community	MSCS		400	29.8	25.3–34.5
			Karachi	Community	MSCS		399	30.1	25.6–34.8
			Lahore	Community	MSCS		400	6.5	4.3–9.4
			Larkana	Community	MSCS		399	16.5	13.0–20.6
			Multan	Community	MSCS		400	0.0	—
			Peshawar	Community	MSCS		180	2.2	0.6–5.6
			Quetta	Community	MSCS		190	9.5	5.7–14.6
			Sarghoda	Community	MSCS		400	51.3	46.2–56.2
			Sukkur	Community	MSCS		399	5.3	3.3–7.9
	Rahman, 2006 [179]	2005	Lahore		CS	All male		0.0	—
	Nai zindagi, 2005 [180]	2005	Faisalabad		SRS	All male	200	9.5	5.8–14.4
			Lahore		SRS	All male	200	2.5	0.8–5.7
			Sarghoda		SRS	All male	100	12.0	6.4–20.0
			Sialkot		SRS	All male	100	1.0	0.0–5.4
	NAP, 2005 [52] (Round I)	2005	Faisalabad	Community	TLS		400	13.3	10.1–17.0
			Hyderabad	Community	TLS		398	25.3	21.2–30.0
			Lahore	Community	TLS		400	3.8	2.1–6.1
			Multan	Community	TLS		400	0.3	0.0–1.4
			Peshawar	Community	TLS		284	0.4	0.0–1.9
			Quetta	Community	TLS		147	9.5	5.3–15.5
			Sukkur	Community	TLS		402	19.2	15.4–23.3
	Bokhari, 2007 [83]	2004	Karachi	Community	TLS	All male	402	23.1	19.1–27.6
			Lahore	Community	TLS	All male	397	0.5	0.1–1.8
	Achakzai, 2007 [181]	2004	Quetta	Community	CS		50	24.0	13.1–38.2
	Bokhari, 2006 [182] (Pilot)	2004–2005	Karachi		TLS		400	26.0	21.8–30.6
			Rawalpindi		CS		199	0.5	0.0–2.8
	Abbasi, 2005 [183]	2004	Larkana	VCT	CS	All male, homeless	3154	8.3	7.4–9.3
	Abbasi, 2009 [184]	2003	Quetta	Voluntary drug treatment center	CS	All male	300	0.3	0.0–1.8
	Altaf, 2007 [74]	2003	Karachi	Harm reduction center	CS	All male, 80% homeless	161	0.6	0.0–3.4
	Kuo, 2006 [70]	2003	Lahore	Harm reduction center	CS	All male	255	0.0	—
			Quetta	Harm reduction center	CS	98% male	96	0.0	—
	Shah, 2004 [185]	2003	Larkana		CS		175	9.7	5.8–15.1
	Altaf, 2003[75]	2002	Karachi	Harm reduction center	CS	All male, 86% homeless	153	0.0	—
	Hadi, 2005 [64]	2002	Rawalpindi, Swat, & Mardan	Mixed	CS	65% male	500	3.4	2.0–5.4
	Akhtar, 2004 [186]	2002	Faisalabad	Voluntary drug treatment center	CS	All male	74	0.0	—
	Nai Zindagi, 1999 [72]	1999	Lahore	Community	CS	All male	200	0.0	—
	Parviz, 2006 [82]	1996	Karachi	Voluntary drug treatment center & community	CS	All male	231	0.4	0.0–2.4
	Baqi, 1998 [81]	1994	Karachi	Voluntary and mandatory drug treatment center	CS	All male	120	0.0	—
	Iqbal, 1996 [187]	1987–2004	Lahore	Clinical setting	CS		77	0.0	—
	Khanani, 2010 [188]	—	Karachi	Clinical setting	CS	Afghani refugees	42	19.0	8.6–34.1
	UrRehman, 2002 [189]	—	National				400	0.0	—
**Syria**	Mental Health Directorate, 2008 [59]	2006	Damascus		SBS	96% male	204	0.5	0.1–2.7
**Tunisia**	MOH, 2013 [53] (Round II)	2011	Tunis		RDS		506	2.9^a^	1.3–4.4^a^
			Bizerte		RDS		301	0.0	—
	MOH, 2010 [54] (Round I)	2009	Tunis, Bizerte, & Sousse		RDS	91% male	715	3.1	1.9–4.6

a Population-adjusted estimate.

b Self-report.

CS, convenience sampling; ID, infectious disease; MSCS, multi-stage cluster sampling; RCS, random cluster sampling; RDS, respondent driven sampling; SBS, snow ball sampling; SRS, simple random sampling; TLS, time location sampling; VCT, voluntary counseling and testing.

Three HIV incidence studies were identified. In Kabul, Afghanistan, HIV incidence among a sample of 479 PWID in 2008 was 2.2/100 person-years (pyr), despite 72% of study participants reporting use of harm reduction services [63]. Among 500 PWID in three cities in Pakistan, HIV incidence was 1.7/100 pyr in 2006 [64]. A very high incidence rate (17.2/100 pyr) was reported in Tehran, Iran, in 2002 among 214 incarcerated PWID [65].

Analysis of notified HIV cases indicated that in 2011, injecting drug use contributed 20% (80/409), 23% (50/216), 38% (6/16), 49% (52/107), and 60% (948/1,588) of all newly notified cases in this year in Egypt, Pakistan, Bahrain, Afghanistan, and Iran, respectively. A smaller contribution was reported in the remaining countries (Table 4).

**Table 4:** Contribution of injecting drug use as a mode of HIV transmission to the total HIV/AIDS cases by country as per various studies/reports and countries' case notification reports [126],[190].

Country	2011 Case Notification Report^a^			Cumulative Data since the Start of the Epidemic
	*n*	*N*	Percent	Percent due to PWID (end year)
Afghanistan	52	107	48.6	44.3% (2011)
Bahrain	6	16	37.5	62.8% (2008)
Egypt	80	409	19.6	1.6% (2008)
Iran	948	1,588	59.7	69.4% (2011)
Iraq	—	—	—	0.0% (2009)
Jordan	0	17	0.0	2.4% (2011)
Kuwait	0	25	0.0	2.2% (2008)
Lebanon	1	51	2.0	6.1% (2009)
Morocco	9	750	1.2	2.7% (2011)
Oman	5	140	3.6	4.3% (2011)
OPT	0	6	0.0	2.8% (2011)
Qatar	0	1	0.0	—
Pakistan	50	216	23.1	33.2% (2008)
Saudi Arabia	46	394	11.7	6.4% (2009)
Syria	0	69	0.0	2.4% (2009)
Tunisia	3	73	4.1	24.4% (2009)
UAE	1	57	1.8	3.6% (2011)
Yemen	1	236	0.4	1.4% (2009)

Only the most recent available report was used.

a Except for Bahrain, Egypt, and Iraq (2010 report) and Pakistan (2008 report).

*n*, number of positive cases that are PWID; *N*, total number of positive cases; Percent, percent of positive cases that are PWID out of the total number of positive cases; UAE, United Arab Emirates.

### HIV Epidemic States

The evidence was sufficient to characterize the HIV epidemic state in 13 countries, summarized in Table 5. Details on how the conclusions were reached are in Text S3.

**Table 5:** Characterization of the state of the HIV epidemic among people who inject drugs in the Middle East and North Africa based on the HIV biological data and quality and scope of the evidence.

Country	Level of HIV Prevalence	Trend in HIV Prevalence	Geographical Distribution	Quality and Scope of Evidence
**Iran**	Concentrated	Established	National	Conclusive
**Pakistan**	Concentrated	Emerging	National	Conclusive
**Afghanistan**	Concentrated	Emerging	At least localized	Good
**Egypt**	Concentrated	Emerging	At least localized	Good
**Morocco**	Concentrated	Emerging	At least localized	Good
**Libya**	Concentrated	Unknown	At least localized	Good
**Bahrain**	At least outbreak-type	—	—	Limited
**Oman**	At least outbreak-type	—	—	Poor
**Jordan**	Low-level	—	—	Good
**Lebanon**	Low-level	—	—	Good
**OPT**	Low-level	—	—	Good
**Tunisia**	Low-level	—	—	Good
**Syria**	Low-level	—	—	Limited
**Djibouti**	Unknown	—	—	Poor
**Iraq**	Unknown	—	—	Poor
**Kuwait**	Unknown	—	—	Poor
**Saudi Arabia**	Unknown	—	—	Poor
**Sudan**	Unknown	—	—	Poor
**Yemen**	Unknown	—	—	Poor
**Algeria**	Unknown	—	—	No evidence
**Qatar**	Unknown	—	—	No evidence
**Somalia**	Unknown	—	—	No evidence
**UAE**	Unknown	—	—	No evidence

Countries are sorted by level of HIV prevalence, trend in HIV prevalence, geographical distribution, quality and scope of evidence, then alphabetical order.

UAE, United Arab Emirates.

#### Concentrated HIV epidemics

**Figure 3:**
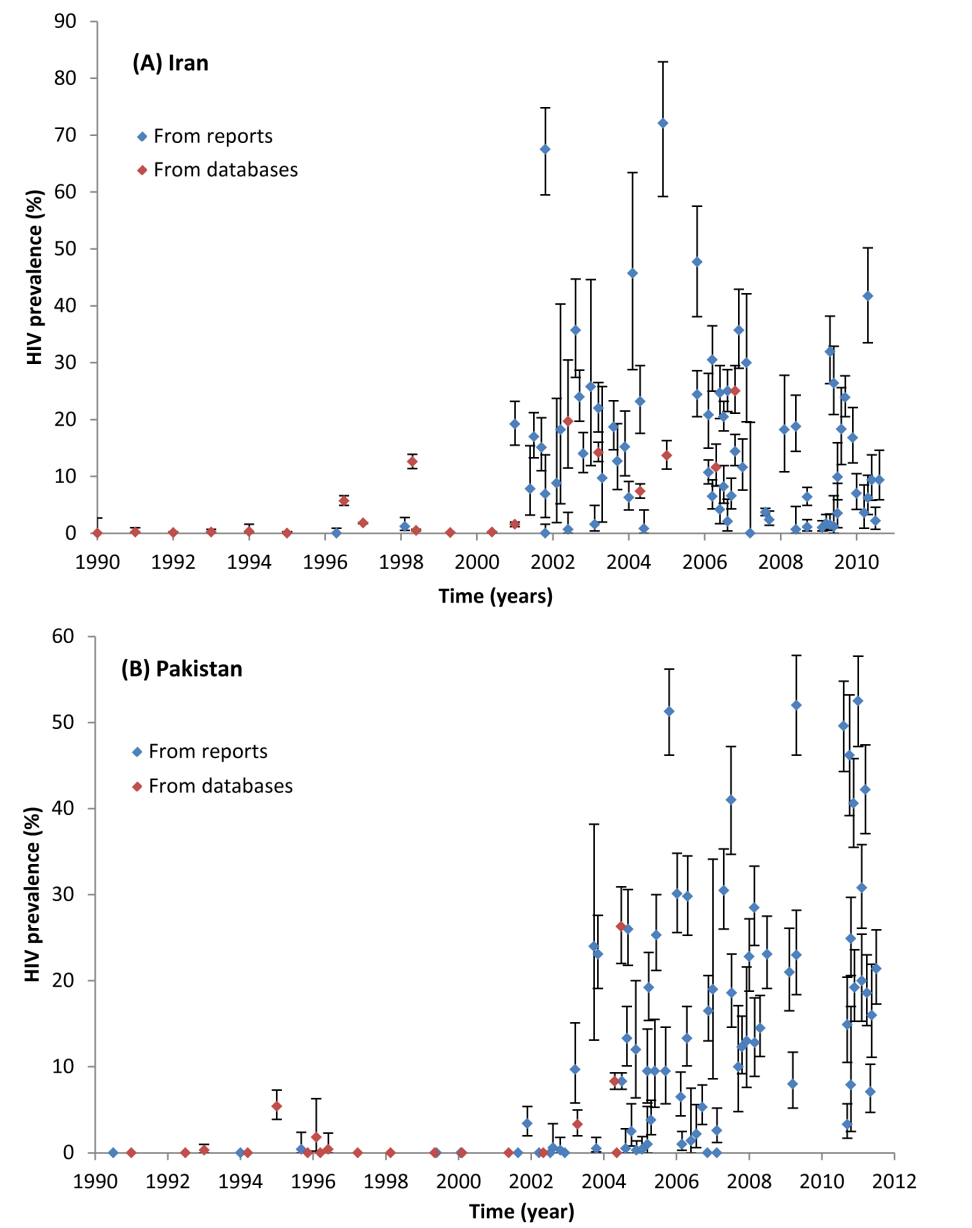
Trend of HIV prevalence among male people who inject drugs in (A) Iran and (B) Pakistan. This graph displays all available HIV prevalence measures for these two countries as extracted from eligible reports (Table 3) and various databases (Table S4). Each dot represents one HIV prevalence measure for the specific year, and the bars around it define the 95% confidence interval. A pattern of established HIV epidemic is observed in Iran (A), while a trend of emerging HIV epidemic is observed in Pakistan (B).

**Figure 4:**
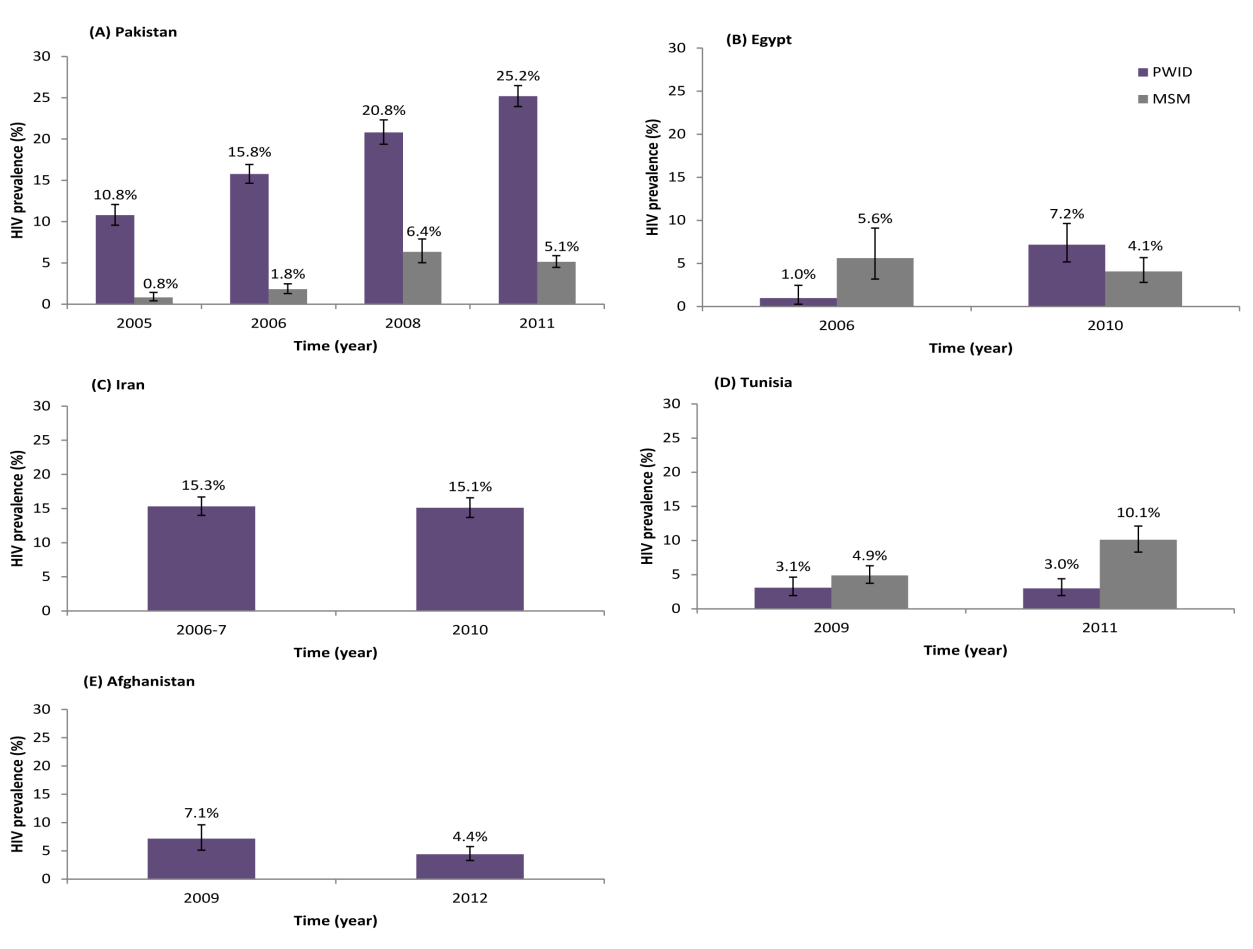
Trend of HIV prevalence among people who inject drugs, and when available men who have sex with men, in repeated rounds of bio-behavioral surveillance surveys. These graphs display the trend of HIV prevalence in repeated rounds of bio-behavioral surveillance surveys using state of the art sampling techniques for hard-to-reach populations including respondent driven sampling and time-location sampling. Country level and aggregate data of multiple cities/provinces are displayed. For consistency between countries and between different rounds within a given country, unadjusted sample estimates are displayed. Three main patterns of HIV epidemics among PWID are depicted. A pattern of emerging concentrated epidemics is observed in Pakistan (A) and Egypt (B); a pattern of established concentrated epidemic is observed in Iran (B); and a pattern of low-level HIV epidemic is observed in Tunisia (D). In Afghanistan (E), there is an emerging epidemic among PWID in apparently only part of the country; the effect of which was diluted in the second round with the inclusion of new cities with still very limited prevalence. The potential overlap of the HIV epidemics among PWID and MSM is depicted in Pakistan and Egypt. In Pakistan, an emerging HIV epidemic among transgender sex workers is observed, but lags the epidemic among PWID (A). In Egypt, the concentrated epidemic among MSM seems to have preceded the epidemic among PWID (B). In Tunisia, the potential link between the MSM and PWID epidemics is not clear because the studies were conducted after the epidemics had already risen.

Concentrated HIV epidemics among PWID were observed in Iran, Pakistan, Afghanistan, Egypt, Morocco, and Libya (Table 5). Iran is the only country with conclusive evidence for an established concentrated epidemic at the national level. The first HIV outbreaks among PWID in Iran were reported around 1996. HIV prevalence then increased considerably in the early 2000s, reaching a peak by the mid-2000s (Figure 3A). HIV prevalence in the 2006 and 2010 multi-city IBBSS was stable at 15% (*n* = 2,853 and *n* = 2,479, respectively) (Figure 4C) [43],[44]. The evidence suggests that the HIV epidemic among PWID in Iran is now established at concentrated levels of about 15%.

Emerging concentrated epidemics were seen in Pakistan, Afghanistan, Egypt, and Morocco (Table 5). For example, in Pakistan, after almost two decades of very low HIV prevalence among PWID, a trend of increasing prevalence was observed after 2003 (Figure 3B). This trend is national and ongoing, reaching over 40% in recent studies and with no evidence yet of stabilization (Figure 3B). This trend also manifests itself in the multi-province IBBSS where HIV prevalence among PWID has steadily increased from 10.8% in 2005 (*n* = 2,431) [52], to 15.8% in 2006 (*n* = 4,039) [51], 20.8% in 2008 (*n* = 2,969) [50], and 25.2% in 2011 (*n* = 4,593) [37] (Figure 4A). In view of the high HIV prevalence, the emerging HIV epidemic in Pakistan is considered advanced. Another example is Egypt where HIV prevalence was also very low for about two decades (Tables 3 and S4), including the first round of IBBSS in 2006 [40],[41], but increased to 6%–7% in both cities surveyed in the most recent IBBSS in 2010 (*n* = 284 and *n* = 274) (Figure 4B) [42]. Consistently, 19.6% of the 409 newly notified HIV cases in 2010 in Egypt were due to injecting drug use, compared with only 1.6% of the total notified cases up to 2008 (Table 4). In Afghanistan (Figure 4E) and Morocco, the HIV epidemic among PWID appears to be emerging in at least parts of the country, with notably high HIV prevalence in some settings, but still low HIV prevalence in others (Table 3).

The HIV epidemic in Libya is also concentrated, but the trend is unclear. Libya has the highest reported prevalence of HIV among PWID in MENA (87.2%, 95% CI 83.1%–90.6% in the IBBSS in Tripoli [47]). Earlier data, although of unclear quality, also indicate prevalence of up to 59% in 2001 among PWID in Tripoli (Table S4). This indicates a concentrated HIV epidemic among PWID in at least part of Libya. Although the epidemic in Tripoli is most likely to be established, the level of evidence overall is insufficient to characterize whether the national epidemic is emerging, with few outbreaks in the past, or is established with endemic HIV transmission among PWID.

#### “At least outbreak-type” HIV epidemics

In Bahrain and Oman, data show that there are, or have been, at least some pockets of HIV infection among PWID, with reported prevalence up to 21.1% (Bahrain, *n* = 242) [60] and 27% (Oman, *n* = 33) [58]. However, most available data are from studies with unknown methodology or high ROB; therefore, the quality of evidence is insufficient to indicate whether there is a concentrated epidemic in these two countries, even if localized.

#### Low-level HIV epidemics

The HIV epidemic among PWID is low-level in Jordan, Lebanon, Tunisia, OPT, and Syria (Table 5). In these countries (except Syria), at least one round of IBBSS has been conducted, in addition to other data; all indicate limited HIV spread among PWID (Figure 4D; Tables 3 and S4). The contribution of injecting drug use to the total notified cases also remains minimal in these countries, further confirming a low-level epidemic (Table 4).

### Injecting Risk Behavior

Table S5 summarizes injecting risk behavior measures among PWID in MENA. The key risk behavior that exposes PWID to HIV infection is the use of non-sterile injecting equipment. Available data indicate that the lifetime prevalence of sharing needles/syringes among PWID in MENA was as high as 71% [45], 79% [66], 85% [47], 95% [67], and 97% [58] in Jordan, Pakistan, Libya, Iran, and Oman, respectively. The median prevalence of sharing in the last injection was 23% (IQR: 18%–28%). In Pakistan, most injecting occurs in groups and in public places, and reported use of “street doctors” or professional injectors was common, which is associated with high reuse of injecting equipment (Table S5) [50].

In MENA, PWID inject drugs at median of 2.2 injections per day, with reported rates of 3.3 [68] and 5.7 [69] injections per day among some PWID in Iran and Afghanistan, respectively. The median age at first injection was 26 years (IQR: 24–28 years), and the median duration of injecting drugs was 4.6 years (IQR: 3.8–6.1 years) (Table S5).

### Sexual Risk Behavior

The majority of PWID in MENA are sexually active (Table S6). On average, 52% have been ever married (IQR: 35%–56%), 43%–89% report having sex with a regular female partner, 29%–60% reported multiple sexual partnerships, and 18%–42% report sex with non-regular female partners in the last year (Table S6). Reported levels of condom use varied but generally were on the low to intermediate range. Overall, 36% of PWID reported ever using condoms (IQR: 20%–54%) with the lowest prevalence in Afghanistan and Pakistan (10%–38% [70]–[76]), and the highest in Lebanon (88% [77]). Condom use during last sex was reported by 4%–38% of PWID, reaching 66% only in Libya [47]. Only 12%–25% of PWID reported consistent condom use in the last year (Table S6).

### Mixing with Other High-Risk Populations

Risk behaviors of PWID in MENA overlap considerably with other high-risk populations, namely MSM and female sex workers. A median of 18% of male PWID in MENA reported ever having sex with men (IQR: 11%–27%), and a median of 7% did so in the last year (IQR: 2%–10%) (Table S6). The highest rates of same-sex sex have been reported in Pakistan. Reported condom use during anal sex was overall very low (Table S6).

PWID in MENA engage in sex work, either through buying or selling sex. A median of 45% reported ever having sex with a sex worker (IQR: 31%–64%), and a median of 23% did so in the last year (IQR: 15%–30%), with generally low levels of condom use (Table S6). Selling sex in the past year was reported by 5%–29% of PWID in Egypt, Iran, Morocco, OPT, and Pakistan (Table S6).

### Proxy Biological Markers of Risk Behavior

There was substantial between-and within-country variation in HCV prevalence among PWID, with a median of 44% (IQR: 31%–64%) (Table 6). Very high HCV prevalence was reported such as in Afghanistan (70%, *n* = 185, Herat), Egypt (63%, *n* = 100, Alexandria), Iran (over 80%, *n* = 386 prisoners, Tehran), Libya (94%, *n* = 328, Tripoli), Pakistan (94%, *n* = 161, Karachi), and Saudi Arabia (75%, *n* = 1,909, Jeddah) (Table 6). These figures are consistent with the reported high levels of sharing of injection equipment, such as in Iran, Pakistan, and Libya (Table S5).

**Table 6:** Prevalence of hepatitis C virus among people who inject drugs in the Middle East and North Africa.

Country	HCV Prevalence		Year	Sample Size	Population Characteristics	City	Source
	%	95% CI					
**Afghanistan**	70.0	63.1–76.8	2012	185	Predominantly male	Herat	[39]
	57.9	49.8–65.6	2009	159	Predominantly male	Herat	[38]
	49.1	43.7–54.6	2006–2008	340	Predominantly male	Herat	[127]
	37.1	31.5–42.9	2009	286	Predominantly male	Kabul	[38]
	36.6	32.1–41.1	2006–2008	463	Predominantly male	Kabul	[127]
	36.1	31.7–40.5	2007–2009	483	Predominantly male	Kabul	[69]
	27.6	23.1–32.5	2012	369	Predominantly male	Kabul	[39]
	25.5	17.4–35.1	2009	102	Predominantly male	Mazar-i-Sharif	[38]
	25.0	17.3–33.6	2012	117	Predominantly male	Charikar	[39]
	24.1	18.1–30.8	2006–2008	187	Predominantly male	Mazar-i-Sharif	[127]
	18.8	14.3–24.3	2012	254	Predominantly male	Mazar-i-Sharif	[39]
	12.5	6.6–20.8	2006–2008	96	Predominantly male	Jalalabad	[127]
	9.5	5.9–13.8	2012	236	Predominantly male	Jalalabad	[39]
**Egypt**	63.0	52.8–72.4	1994	100		Alexandria	[130]
**Iran**	95.4	77.2–99.9	2002–2003	22	Prisoners	Gorgan	[160]
	88.9	80.0–94.8	2003	81	Predominantly male prisoners	Guilian	[191]
	80.5	76.3–84.4	2001–2002	386	Predominantly male prisoners	Tehran	[162]
	80.1	76.2–83.7	1998	464		Shiraz	[168]
	80.0	76.0–83.5	2006	454	Predominantly male	Tehran	[192]
	76.8	72.4–80.9	2003	401	Predominantly male prisoners	Isfahan, Chaharmahal Bakhtiary, & Lorestan	[154]
	67.4	49.5–82.6	—	34		Tehran	[173]
	65.9	59.8–71.7	2008	258	Predominantly male	Tehran	[140]
	64.8	58.4–70.6	2002	249	Prisoners	Hormozgan	[159]
	63.3	56.2–70.0	2005–2007	199		Hamadan	[193]
	59.4	47.4–68.7	2008	117	Predominantly male	Foulad-Shahr	[141]
	59.4	49.2–69.1	2001–2002	101	Prisoners	Mashhad	[166]
	52.9	35.1–70.2	2007	34	Predominantly male, homeless	Tehran	[194]
	52.1	43.6–60.6	2001–2006	142	Predominantly male	Ahfaz	[164]
	52.0	44.9–59.0	2004	202	Predominantly male	Tehran	[195]
	50.0	11.8–88.2	2008–2009	6	Incarcerated juveniles	Isfahan	[196]
	50.0	21.1–78.9	2006	12		Kermanshah	[197]
	50.0	41.4–58.6	—	138	Predominantly male	Shiraz	[170]
	47.1	42.8–51.4	2008–2009	531	Predominantly male	Isfahan	[198]
	45.3	40.3–50.3	1995	402	Predominantly male prisoners	Tehran	[199]
	44.4	27.9–61.9	2006–2007	36	Females	Tehran	[146]
	43.4	40.8–45.9	—	1,485	Predominantly male	Foulad-Shahr	[200]
	43.4	40.2–46.6	—	936	Predominantly male	National	[172]
	42.4	—	2009–2010	—		Kohgiloyeh & Boyerahmad	[201]
	42.0	38.8–45.2	—	951	Prisoners	Foulad-Shahr	[202]
	41.6	38.4–44.8	2008–2009	943	Predominantly male prisoners	Isfahan	[203]
	40.3	34.0–46.9	2012–2013	233	Predominantly male	Shiraz	[134]
	38.6	32.1–45.2	2010	226	Predominantly male	Tehran, Shiraz, & mashhad	[79]
	37.5	20.4–54.9	2007–2009	33		Sari	[142]
	36.6	21.6–52.0	2010	42	Female sexual partners of PWIDs	Tehran, Shiraz, & mashhad	[79]
	36.5	28.2–45.2	2001–2002	132	Predominantly male	Tehran	[162]
	36.0	24.6–48.1	2007	70		Tehran	[145]
	34.1	30.9–37.4	2006–2007	859	Predominantly male	Tehran	[146]
	34.0	31.8–36.3	2008–2009	1,747	Predominantly male	Isfahan	[204]
	31.5	24.2–39.7	2002	149	Predominantly male prisoners	Hamadan	[161]
	30.9	26.0–36.2	2002–2006	333	Predominantly males, hospitalized for infectious disease	Ahfaz	[158]
	22.8	9.6–41.1	2000–2005	31	Hospitalized for infectious disease	Zahedan	[167]
	16.1	5.5–33.7	2003	31		Rafsanjan	[157]
	13.0	2.8–33.6	2001–2006	23	Females, hospitalized for infectious disease	Kashan	[163]
	12.9	2.8–33.6	2006	23		Tehran & Hormozgan	[56]
	11.9	7.5–17.6	2001–2006	177	Predominantly males, hospitalized for infectious disease	Kashan	[163]
	11.3	6.5–17.9	2004	133	Predominantly male	Shahr-e-Kord	[152]
	26.8^a^	20.9–33.3	2011	209	Predominantly male	Tehran	[135]
**Lebanon**	51.0	33–74	2007–2008	43		Beirut	[46]
	5.0	0.6–16.9	2000–2002	40	25% female	Beirut	[175]
**Libya**	94.2	90.8–96.7	2010	328	Predominantly male	Tripoli	[47]
**Morocco**	79.2	72.1–85.7	2011–2012	274	Predominantly male	Nador	[117]
	45.6	35.5–56.6	2010–2011	261	Predominantly male	Tanger	[117]
**Oman**	36.0^a^	12.8–64.9	—	14	Predominantly male	Muscat	[58]
	11.0^a^	0.3–48.2	—	9	Predominantly male prisoners	Muscat	[58]
	53.0^a^	40.0–66.3	—	60	Predominantly male	Muscat	[58]
**OPT**	40.3	29.2–52.2	2010	192	Predominantly male	East Jerusalem	[177]
**Pakistan**	94.3	89.7–97.4	2003	161		Karachi	[74]
	92.9	89.1–95.8	2003	255	Predominantly male	Lahore	[70]
	91.8	88.6–94.4	2004	380	Predominantly male	Lahore	[205]
	89.0	83.8–93.0	1999	200	Predominantly male	Lahore	[72]
	89.0	85.5–91.9	—	400		National	[189]
	87.0	83.3–90.1	2004	399	Predominantly male	Karachi	[205]
	86.9	80.5–91.8	2002	153	Homeless	Karachi	[75]
	76.0	—	2005	—	Predominantly male	Lahore	[179]
	75.0	65.1–83.3	2003	96	Predominantly male	Quetta	[70]
	62.5	24.5–91.5	2007–2009	8	Remote rural population	Kech	[206]
	60.0	45.2–73.6	2004	50		Quetta	[181]
	46.4	34.5–57.9	—	76	Predominantly male prisoners	Kabul	[207]
	45.2	29.8–61.3	—	42	Afghani refugees	Karachi	[188]
	44.7	39.0–50.5	2003	300	Predominantly male	Quetta	[184]
	42.0	37.6–46.5	2002	500	35% female	mix of cities	[64]
	31.5	25.1–38.4	—	200	Predominantly male	Khyber pakhtunkhwa	[208]
	17.3	13.1–22.0	2007	302	Predominantly male	Rawalpindi	[178]
	14.3	5.4–28.5	—	42		Khyber pakhtunkhwa	[209]
	8.0	3.4–14.9	2007	102	Predominantly male	Abotabad	[178]
**Saudi Arabia**	74.6	72.6–76.5	1995–1996	1909	Predominantly male	Jeddah	[210]
	69.0	64.7–72.9	1995–1996	505			[211]
	38.1	32.9–43.4	—	344			[212]
**Syria**	60.5	43.4–76.0	2006	38	Predominantly male	Damascus	[213]
	21.0^a^	11.4–33.9	2006	57	Predominantly male	Damascus	[59]
**Tunisia**	35.8	29.1–42.5	2011	506	Predominantly male	Tunis	[53]
	29.1	25.8–32.6	2009	701	Predominantly male	Tunis, Bizerte, & Sousse	[54]
	2.4	0.6–4.1	2011	301	Predominantly male	Bizerte	[53]

The table is sorted by country then by descending order of HCV prevalence.

a Self report.

Available data on the prevalence of syphilis among PWID in Egypt, Iran, Afghanistan, and Pakistan indicate relatively high prevalence up to 3%, 8%, 17%, and 18%, respectively (Table S6). Considerable herpes simplex virus type 2 prevalence has been reported among PWID in Afghanistan (4%–21%) and Pakistan (6%–19%) (Table S6). Data on the prevalence of gonorrhoea (0%–1.8%) and chlamydia (0%–0.7%) were available only in Pakistan (Table S6).

### Knowledge of HIV/AIDS

Levels of basic HIV/AIDS knowledge among PWID in MENA were high overall, with a median of 93% having ever heard of HIV/AIDS (IQR: 72%–99%). Still, there was much variation in the proportion of PWID who correctly identified reuse of non-sterile needles/syringes and unprotected sex as modes of HIV transmission (Table S7). Only a median of 45% (IQR: 30%–63%) of PWID perceived themselves at risk of HIV/AIDS (Table S7). With a few exceptions of high levels of HIV testing such as in Lebanon and Oman, the median prevalence of lifetime testing among PWID ranged between 8% (Egypt) and 45% (Iran) (Table S7).

## Discussion

### Injecting Drug Use in MENA

We estimate that there are 626,000 PWID in the MENA region. Overall, the mean prevalence of injecting drug use (0.24%) is comparable with global figures which range from 0.06% in South Asia to 1.50% in Eastern Europe [31]. Prevalence of injecting drug use in MENA varied between countries and was higher in the eastern part of the region. Injecting drug use appears to be heavily concentrated among men; but female PWID are one of the hardest-to-reach populations in MENA, thereby limiting our knowledge of this vulnerable group. From limited available data, it appears that injecting drug use among females has a strong association with sex work and having a PWID sexual partner [78],[79].

### Emerging HIV Epidemics and HIV Epidemic Potential among PWID

After synthesizing a large body of data, we documented HIV epidemics among PWID in one-third of MENA countries. The HIV epidemic is in a concentrated state in about half the countries with available data. Iran is the only country with an established concentrated epidemic, while the most common pattern is that of emerging concentrated epidemics. Most observed epidemics in the region are recent, occurring only in the last decade; around the same time that HIV epidemics among MSM appear to have emerged (2003) [14]. Of note, our classification of epidemic states did not depend only on the size of the epidemic, but also on the trend of HIV prevalence and other biological data. For example in Pakistan, despite high HIV prevalence, the epidemic was classified as emerging since HIV prevalence continues in an increasing trend. HIV prevalence among PWID in MENA countries with concentrated epidemics is overall in the range of 10%–15%, which is in the intermediate range compared to global figures [31]. However, there are settings with very high prevalence, most notably in Tripoli, Libya, which appears to have one of the highest HIV prevalence reported globally (87.1%) [31],[47].

In about 20% of MENA countries, the HIV epidemic among PWID was low level, with HIV prevalence consistently low for many years including the most recent IBBSS. In some countries, such as Jordan, Lebanon, and OPT, no HIV infections were found in the IBBSS. The available evidence in countries at low level is restricted to a few cities, and there could be hidden sub-epidemics in other sites. Nevertheless, the low prevalence could be reflective of the intrinsic HIV transmission dynamics, levels of risk behavior, and/or injecting network structure. HIV may not have been introduced to the PWID community, may have been recently introduced, or may have been spreading slowly and inefficiently for some time. The latter may reflect injecting networks with infrequent and few repeated transmission contacts among PWID to sustain HIV dynamics. In Lebanon and Syria for example, it appears that PWID form closed small networks with injecting occurring in private homes and among friends, and not in large groups or at shooting galleries [59],[80]. The low prevalence could also be a consequence of stochastic effects where the small number of individuals who introduced HIV to the PWID population happened by chance not to have links that could sustain transmission chains.

Whilst it is conceivable that HIV prevalence may not grow in countries currently at low level, there are settings where HIV prevalence increased considerably in a short period of time. For example in Karachi, Pakistan, after several years of near zero prevalence [74],[75],[81],[82], HIV prevalence in 2004 increased to 23% in less than 6 months [83], and reached 42% in 2011 [37]. This pattern is not surprising given the reported risky practices and high HCV prevalence. When HIV prevalence was still very low in Karachi, HCV prevalence was over 85% [74],[75], indicating substantial use of non-sterile injecting equipment and suggesting connectivity of injecting networks. In Iran, the substantial HCV prevalence (up to 80%) was predictive of the explosive HIV epidemic that occurred subsequently. In both Iran and Pakistan, injecting networks often seem to be well connected and we found reports of injecting and sharing occurring among persons who are not necessarily socially related, e.g. in shooting galleries [84],[85]. Data on HCV prevalence among PWID in MENA countries with low-level HIV epidemics are limited. However, HCV prevalence of 40%–61% among some PWID groups such as in Lebanon, OPT, and Syria suggest moderate HIV epidemic potential once the virus is introduced to the PWID community.

### Bridging of the HIV Epidemic to Other Population Groups

We found considerable overlap of risk behavior between PWID and other high-risk groups in MENA; this could play a role in emerging HIV epidemics, as it creates opportunities for an infection circulating in one population to be bridged to another one. In Pakistan, the rapidly growing HIV epidemic among PWID was followed closely by an emerging epidemic among transgender sex workers (Figure 4A). Phylogenetic analyses found clustering of subtypes between the two populations, suggesting that the infection might have bridged from PWID to the transgender population [86]. A similar pattern, but in the opposite direction, may have occurred in Egypt where an emerging epidemic among MSM [14] preceded the nascent epidemic among PWID (Figure 4B). While supported by behavioral data [40],[42], this needs to be confirmed by phylogenetic analyses.

Our analysis, focused on the HIV disease burden among PWID, still masks the role of these epidemics in driving the onward transmission of HIV to the sexual partners of PWID and further in the population. The majority of PWID are sexually active and about half are married. They often engage in risky sexual behavior as confirmed by the prevalence of STIs. This puts sexual partners of PWID at risk of HIV. A substantial number of infections in MENA have been documented in women who acquired HIV from their PWID husbands; and in some countries, the majority of HIV infections among women were acquired from a PWID sexual partner [79],[87]–[89]. This highlights the vulnerability of sexual partners of PWID, who are often female spouses. An illustration of the role of the HIV epidemics among PWID in driving the onward transmission of HIV emerges from recent mode of transmission (MoT) modeling studies in the region [90]–[92]. For example, in Iran, PWID directly contributed 56% of the total HIV Incidence; and indirectly, only through infections to their current sexual partners, an additional 12% of the total incidence [92]. More onward HIV transmissions would arise if the sexual partners of PWID transmitted the infection to their other sexual partners.

### Study Limitations

One limitation of our study is that the quantity and quality of data varied by countries. There were virtually no HIV data in four countries, and the data quality in six others was insufficient to assess the status of the epidemic. Longitudinal repeated IBBSS data were available in only five countries. Six countries have recently conducted their first round of IBBSS; and in most of these, subsequent rounds are either planned or being implemented. The quality of data was “good” or “conclusive” in ten out of the 23 countries.

While most of the data were from cross sectional surveys, there was a substantial improvement in the quality of data over time. Many studies were conducted with state of the art research methodologies in HIV research. These consist of IBBSS studies using innovative sampling methodologies for hard-to-reach populations such as respondent-driven sampling and time-location sampling. Most of these studies benefited from large sample sizes and some from broad geographical coverage at the national level.

Of note that in several countries there were no recent national estimates of the number and proportion of PWID. The only national data available for these countries were extracted from earlier global reviews of injecting drug use [4],[31]. The reviews were based mainly on estimates by the Reference Group to the UN on HIV and Injecting Drug Use, which systematically collects and analyses global data on injecting drug use and HIV [32]. The Reference Group is considered the main reference for PWID estimates globally, providing the estimates to the United Nations Office on Drugs and Crime (UNODC), WHO, and UNAIDS secretariats. We complemented the Reference Group data with PWID national risk-group size estimation studies that were conducted in the last few years in five countries namely Afghanistan, Iran, Pakistan, Saudi Arabia, and Tunisia. Since we partly relied on secondary sources of data and since the data that we used came from studies using different methodologies, our pooled estimates of the number and prevalence of PWID in MENA should be considered as approximate figures.

In assessing the status of the epidemic at the country-level, we did not limit our analysis to one line of evidence, but synthesized and corroborated findings from different data sources and types such as HIV prevalence and incidence, notified HIV cases, injecting and sexual risk behavior, and other related and contextual data. Thus we could make a comprehensive assessment of the epidemic status and address potential limitations in any one line of evidence [93]. We did a rigorous appraisal of the scope and quality of the evidence within each country by assessing the amount and geographical coverage of available data, as well as the ROB and precision of individual point estimates. A qualifier for the scope and quality of the evidence at the country level was integrated with each HIV epidemic state assigned. Our search criteria were expansive, covering different literature sources. Before the present submitted work, the status of the epidemic across MENA country was poorly understood. On the basis of our integrated data synthesis and using rigorous methodology and data quality assessment, we were able to concretely qualify the epidemic status in 13 countries (over half of MENA countries), and to document the overall trend of emerging epidemics. The lack of evidence in several MENA countries does not preclude the possibility of hidden epidemics among PWID in these settings.

### HIV Response among PWID in MENA

Not only does the region overall lag behind in responding to the emerging HIV epidemics among PWID; in occasions misguided policy has contributed to these epidemics. Most notably in Libya, the large HIV epidemic among PWID appears to have been exacerbated by restrictions imposed on the sale of needles and syringes at pharmacies in the late 1990s [11],[94]. Overall, harm reduction programs still remain limited in MENA, and there is a need to integrate such programs within the socio-cultural framework of the region [95]. Several countries though have made significant strides in initiating such programs in recent years [11],[96]. Needle/syringe exchange programs are currently implemented in nine countries, and opioid substitution therapy in five [96]. Iran remains the leader in the provision of harm reduction services to PWID with the highest coverage of needle/syringe exchange programs in the region [12],[96]. It appears also to be the only country in MENA to provide such services in prisons [96],[97] and to provide female-operated harm reduction services targeted at female drug users [96].

Iran has also initiated triangular clinics that integrate services for treatment and prevention of injecting drug use, HIV/AIDS, and other STIs; and these clinics have received international recognition as best practice [98]–[100]. Among other interventions implemented in Iran are drop-in centers, integration of substance use treatment and HIV prevention into the rural primary health care system, and community education centers [62],[101]–[105]. These efforts appear to have been successful in reducing sharing of injecting equipment [106]–[108], though the coverage of harm reduction continues to be lower than adequate [104].

Other countries in the region have also made progress in revising their policies, adopting harm reduction programs, and integrating such programs in their national strategic plans such as Afghanistan, Egypt, Lebanon, Morocco, Pakistan, and Tunisia [11],[109]. Access to antiretroviral therapy (ART) has also expanded in MENA in recent years, and treatment outcomes reported by country ART programs are comparable to globally reported outcomes [110],[111]. Good adherence to ART has been also observed, such as in Morocco [112], though some non-adherence and treatment interruptions, among other obstacles, have been also reported in several countries [112]–[114].

Non-governmental organizations (NGOs) have been instrumental to the success in harm reduction in MENA. It can be noted that in countries where NGOs are strong, HIV response has been also strong [11],[109]. The Iranian NGO Persepolis, for example, played an important role in the transformation to effective policies in Iran [115]. Building on the growing role of NGOs, a regional civil society network was established in 2007 covering 20 countries in MENA; the Middle East and North Africa Harm Reduction Association (MENAHRA) [116]. MENAHRA has the objective of building the capacity of civil society organizations in harm reduction efforts through training, sharing of information, networking and providing direct support to NGOs to initiate or scale-up harm reduction services. The network is a collaborative initiative by regional and international organizations with funding from international donors, and has been influential in promoting harm reduction.

Despite the recent progress in harm reduction, HIV prevention efforts among PWID in MENA remain impeded by generic and routine planning, competing priorities, limited human capital, and lack of monitoring and evaluation [7]. National policies remain inadequate and not sufficiently reflecting evidence-informed approaches [7]. The scope and coverage of prevention services remain patchy across and within countries [11],[96],[109]. An indicator of the low effective coverage is that only a minority of PWID report ever being tested, and a smaller proportion report being tested within the last year [11]. In Morocco and Pakistan, two countries with a strong HIV response, only 32.5% [117], 47.8% [117], 6.1% [51], and 20.7% [50] of PWID in different surveys reported ever being tested. Even where services are available, PWID may not be aware of them, and when aware of them, they may not utilize them. In Pakistan for example, 37% of PWID in one study were aware of HIV prevention programs in their city, but only 19% ever used them [52]. There is an urgent need to expand the provision, scope, and coverage of HIV interventions among PWID in MENA to be ahead of the growing HIV epidemics.

### Conclusion

Our study identified a large volume of HIV-related biological and behavioral data among PWID in the MENA region, including quality data that appear in the scientific literature for the first time. The in-depth analyses, the quality assessment of evidence, and the comprehensive synthesis of data facilitated, for the first time to our knowledge, a rigorous characterization of the state of the epidemic among PWID across different countries in this region.

We found robust evidence for HIV epidemics among PWID in multiple countries, most of which have emerged only recently and continue to grow. The high risk and vulnerability context suggest potential for further HIV spread. HIV surveillance among PWID must be expanded to detect and monitor these budding and growing HIV epidemics, and to inform effective HIV policy and programming. This mainly includes conducting IBBSS studies among PWID in countries where such surveys have not been conducted yet, and implementing subsequent rounds, for the provision of longitudinal data, in countries that are already developing their surveillance base. Population size estimations and mapping and ethnographic studies are also needed for a better understanding of the profile and injecting and sexual networks of PWID in MENA.

The window of opportunity to control the emerging epidemics should not be missed. HIV prevention among PWID must be made a priority for HIV/AIDS strategies in MENA; and obstacles must be addressed for the provision of comprehensive services and enabling environments for PWID [118]. There is need to review current HIV programs among PWID in light of the emerging epidemics, and to develop service delivery models with embedded links between community-based prevention (needle/syringe exchange programs and condom provision), HIV testing, and treatment (opioid substitution and ART). Such comprehensive approach has already proven its utility in preventing HIV transmission among PWID [119]–[121], but would require better resource allocation and sufficient services in priority areas for PWID.

Prevention efforts need to prioritize those most likely to be reluctant to approach facility-based services, and those with multiple and overlapping risks. Outreach and peer education can provide a means to reach those most at risk with information and services. Access to ART should be expanded in such a region with one of the lowest ART coverage globally [122]. Such expansion must address the low diagnosis rate among people living with HIV [110]. Reaching the at-risk populations even in discreet unpublicized ways would contribute positively to HIV prevention [14],[123]. Improving HIV programming among PWID in MENA is essential not only to confront the growing HIV problem in this population group, but also to prevent the onward transmission of HIV, and the bridging of the infection to other groups as has already occurred in parts of the region.

## Supporting Information

Table S1 **Precision and risk of bias of individual HIV prevalence measures among people who inject drugs in the Middle East and North Africa as extracted from eligible reports.**
(DOCX)

Table S2 **Summary of precision and risk of bias of HIV prevalence measures as extracted from eligible reports.**
(DOCX)

Table S3 **Subnational estimates of the number and prevalence of people who inject drugs in the Middle East and North Africa.**
(DOCX)

Table S4 **HIV point-prevalence measures among people who inject drugs as extracted from various databases including the US Census Bureau database, the WHO/EMRO testing database, the UNAIDS epidemiological fact sheets databases, and other sources of data with unidentified reports.**
(DOCX)

Table S5 **Measures of injecting risk behavior among people who inject drugs in the Middle East and North Africa.**
(DOCX)

Table S6 **Measures of sexual risk behavior and sexually transmitted infections prevalence among people who inject drugs in the Middle East and North Africa.**
(DOCX)

Table S7 **HIV/AIDS knowledge, perception of risk, and HIV testing among people who inject drugs in the Middle East and North Africa.**
(DOCX)

Text S1 **PRISMA checklist.**
(DOCX)

Text S2 **Search criteria.**
(DOCX)

Text S3 **Narrative justification for quality of the evidence and status of the epidemic at the country level.**
(DOCX)

## Abbreviations

ART: antiretroviral therapy
HCV: hepatitis C virus
IBBSS: integrated bio-behavioral surveillance surveys
IQR: interquartile range
MENA: Middle East and North Africa
MSM: men who have sex with men
NGO: non-governmental organization
OPT: Occupied Palestinian Territories
PWID: people who inject drugs
pyr: person-years
ROB: risk of bias
STI: sexually transmitted infection
UNAIDS: Joint United Nations Programme on HIV/AIDS
WHO/EMRO: Eastern Mediterranean Regional Office of the World Health Organization
