# Supplementary material for: HIV among People Who Inject Drugs in the Middle East and North Africa: Systematic Review and Data Synthesis
Source: PLoS Med. 2014 Jun 17;11(6):e1001663. doi: 10.1371/journal.pmed.1001663 (PMC4061009; doi:10.1371/journal.pmed.1001663)
Supplement: Table S1 — Precision and risk of bias of individual HIV prevalence measures among people who inject drugs in the Middle East and North Africa as extracted from eligible reports. (DOCX) [file pmed.1001663.s001.docx]

**Table S1. Precision and risk of bias of individual HIV prevalence measures among predominantly male people who inject drugs in the Middle East and North Africa as extracted from eligible reports**

| **Country** | **Year** | **HIV prevalence** | **Precision** | **Risk of bias** | | |
| --- | --- | --- | --- | --- | --- | --- |
|  |  | **(%)** |  | **HIV ascertainment** | **Sampling** | **Response rate** |
| **Afghanistan** | 2012 | 13.3 [[1](#_ENREF_1)] | Good precision | Low ROB | Low ROB | Low ROB |
|  | 2012 | 2.4 [[1](#_ENREF_1)] | Good precision | Low ROB | Low ROB | Low ROB |
|  | 2012 | 0.3 [[1](#_ENREF_1)] | Good precision | Low ROB | Low ROB | Low ROB |
|  | 2012 | 1.0 [[1](#_ENREF_1)] | Good precision | Low ROB | Low ROB | Low ROB |
|  | 2012 | 0.9 [[1](#_ENREF_1)] | Good precision | Low ROB | Low ROB | High ROB |
|  | 2009 | 18.2 [[2](#_ENREF_2)] | Good precision | Low ROB | Low ROB | High ROB |
|  | 2009 | 3.2 [[2](#_ENREF_2)] | Good precision | Low ROB | Low ROB | Low ROB |
|  | 2009 | 1.0 [[2](#_ENREF_2)] | Good precision | Low ROB | Low ROB | High ROB |
|  | 2007-9 | 2.1 [[3](#_ENREF_3)] | Good precision | Low ROB | Low ROB | Unclear |
|  | 2006-8 | 3.2 [[4](#_ENREF_4)] | Good precision | Low ROB | Low ROB | Unclear |
|  | 2006-8 | 0.0 [[4](#_ENREF_4)] | Low precision | Low ROB | Low ROB | Unclear |
|  | 2006-8 | 0.0 [[4](#_ENREF_4)] | Good precision | Low ROB | Low ROB | Unclear |
|  | 2005-6 | 3.0 [[5](#_ENREF_5)] | Good precision | Low ROB | Low ROB | Unclear |
| **Bahrain** | 1991 | 21.1 [[6](#_ENREF_6)] | Good precision | Low ROB | High ROB | Low ROB |
| **Egypt** | 2010 | 6.5 [[7](#_ENREF_7)] | Good precision | Low ROB | Low ROB | Low ROB |
|  | 2010 | 6.8 [[7](#_ENREF_7)] | Good precision | Low ROB | Low ROB | Low ROB |
|  | 2008-11 | 1.4 [[8](#_ENREF_8)] | Good precision | Low ROB | High ROB | Unclear |
|  | 2006 | 0.6 [[9](#_ENREF_9)] | Good precision | Low ROB | Low ROB | Low ROB |
|  | 1994 | 0.0 [[10](#_ENREF_10)] | Good precision | Low ROB | High ROB | Unclear |
|  | -- | 0.0 [[11](#_ENREF_11)] | Low precision | Low ROB | High ROB | Unclear |
|  | -- | 7·6 [[12](#_ENREF_12)] | Low precision | Low ROB | High ROB | Unclear |
|  | -- | 0·0 [[13](#_ENREF_13)] | Low precision | Low ROB | High ROB | Unclear |
| **Iran** | 2012-3 | 7.7 [[14](#_ENREF_14)] | Good precision | Low ROB | High ROB | Unclear |
|  | 2011 | 2.9 [[15](#_ENREF_15)] | Good precision | High ROB | High ROB | Unclear |
|  | 2010 | 31.9 [[16](#_ENREF_16)] | Good precision | Low ROB | High ROB | Unclear |
|  | 2010 | 26.4 [[16](#_ENREF_16)] | Good precision | Low ROB | High ROB | Unclear |
|  | 2010 | 23.9 [[16](#_ENREF_16)] | Good precision | Low ROB | High ROB | Unclear |
|  | 2010 | 18.3 [[16](#_ENREF_16)] | Good precision | Low ROB | High ROB | Unclear |
|  | 2010 | 16.8 [[16](#_ENREF_16)] | Good precision | Low ROB | High ROB | Unclear |
|  | 2010 | 9.4 [[16](#_ENREF_16)] | Good precision | Low ROB | High ROB | Unclear |
|  | 2010 | 7.0 [[16](#_ENREF_16)] | Good precision | Low ROB | High ROB | Unclear |
|  | 2010 | 6.2 [[16](#_ENREF_16)] | Good precision | Low ROB | High ROB | Unclear |
|  | 2010 | 3.6 [[16](#_ENREF_16)] | Good precision | Low ROB | High ROB | Unclear |
|  | 2010 | 2.2 [[16](#_ENREF_16)] | Good precision | Low ROB | High ROB | Unclear |
|  | 2010 | 9.4 [[17](#_ENREF_17)] | Good precision | Low ROB | High ROB | Low ROB |
|  | 2009-10 | 9.9 [[18](#_ENREF_18)] | Good precision | Low ROB | High ROB | Unclear |
|  | 2009 | 1.2 [[19](#_ENREF_19)] | Low precision | Low ROB | High ROB | Unclear |
|  | 2009 | 1.0 [[19](#_ENREF_19)] | Good precision | Low ROB | High ROB | Unclear |
|  | 2009 | 1.7 [[19](#_ENREF_19)] | Good precision | Low ROB | High ROB | Unclear |
|  | 2009 | 3.5 [[19](#_ENREF_19)] | Good precision | Low ROB | High ROB | Unclear |
|  | 2009 | 1.5 [[19](#_ENREF_19)] | Good precision | Low ROB | High ROB | Unclear |
|  | 2008-9 | 1.1 [[20](#_ENREF_20)] | Good precision | Low ROB | High ROB | Unclear |
|  | 2008-9 | 6.4 [[21](#_ENREF_21)] | Good precision | Low ROB | High ROB | Unclear |
|  | 2008 | 18.8 [[22](#_ENREF_22)] | Good precision | Low ROB | High ROB | Unclear |
|  | 2008 | 0.7 [[23](#_ENREF_23)] | Good precision | Low ROB | Low ROB | Low ROB |
|  | 2007-8 | 3.7 [[24](#_ENREF_24)] | Good precision | Low ROB | High ROB | Unclear |
|  | 2007-8 | 2.4 [[25](#_ENREF_25)] | Good precision | Low ROB | High ROB | Unclear |
|  | 2007-9 | 18.2 [[26](#_ENREF_26)] | Low precision | Low ROB | High ROB | Unclear |
|  | 2007 | 6.6 [[27](#_ENREF_27)] | Good precision | Low ROB | Low ROB | Unclear |
|  | 2007 | 30.0 [[28](#_ENREF_28)] | Low precision | Low ROB | High ROB | Unclear |
|  | 2006-7 | 10.7 [[29](#_ENREF_29)] | Good precision | Low ROB | High ROB | Unclear |
|  | 2006 | 24.4 [[30](#_ENREF_30)] | Good precision | Low ROB | High ROB | Low ROB |
|  | 2006-7 | 8.2 [[31](#_ENREF_31)] | Good precision | Low ROB | Low ROB | Unclear |
|  | 2006-7 | 24.7 [[31](#_ENREF_31)] | Good precision | Low ROB | Low ROB | Unclear |
|  | 2006-7 | 20.8 [[31](#_ENREF_31)] | Good precision | Low ROB | Low ROB | Unclear |
|  | 2006-7 | 30.5 [[31](#_ENREF_31)] | Good precision | Low ROB | Low ROB | Unclear |
|  | 2006-7 | 6.5 [[31](#_ENREF_31)] | Good precision | Low ROB | Low ROB | Unclear |
|  | 2006-7 | 4.2 [[31](#_ENREF_31)] | Good precision | Low ROB | Low ROB | Unclear |
|  | 2006-7 | 35.7 [[31](#_ENREF_31)] | Good precision | Low ROB | Low ROB | Unclear |
|  | 2006-7 | 11.6 [[31](#_ENREF_31)] | Good precision | Low ROB | Low ROB | Unclear |
|  | 2006-7 | 2.1 [[31](#_ENREF_31)] | Good precision | Low ROB | Low ROB | Unclear |
|  | 2006-7 | 14.4 [[31](#_ENREF_31)] | Good precision | Low ROB | Low ROB | Unclear |
|  | 2006-7 | 25.0 [[32](#_ENREF_32)] | Good precision | Low ROB | Low ROB | Unclear |
|  | 2005-6 | 47.7 [[33](#_ENREF_33)] | Good precision | Low ROB | High ROB | Unclear |
|  | 2004 | 6.3 [[34](#_ENREF_34)] | Good precision | Low ROB | High ROB | Unclear |
|  | 2004 | 0.8 [[35](#_ENREF_35)] | Good precision | Low ROB | High ROB | Low ROB |
|  | 2004-5 | 72.1 [[36](#_ENREF_36)] | Low precision | Low ROB | High ROB | Low ROB |
|  | 2004 | 23.2 [[37](#_ENREF_37)] | Good precision | Low ROB | High ROB | Low ROB |
|  | 2003-6 | 45.7 [[38](#_ENREF_38)] | Low precision | Low ROB | High ROB | Unclear |
|  | 2003 | 14.0 [[39](#_ENREF_39)] | Good precision | Low ROB | High ROB | Unclear |
|  | 2003-4 | 15.2 [[40](#_ENREF_40)] | Good precision | Low ROB | High ROB | Unclear |
|  | 2003 | 24.0 [[41](#_ENREF_41)] | Good precision | Low ROB | High ROB | Low ROB |
|  | 2003 | 22.0 [[41](#_ENREF_41)] | Good precision | Low ROB | High ROB | Low ROB |
|  | 2003 | 9.7 [[42](#_ENREF_42)] | Low precision | Low ROB | High ROB | Unclear |
|  | 2002-6 | 18.0 [[43](#_ENREF_43)] | Good precision | Low ROB | High ROB | Unclear |
|  | 2002 | 15.1 [[44](#_ENREF_44)] | Good precision | Low ROB | Low ROB | Unclear |
|  | 2002-3 | 18.2 [[45](#_ENREF_45)] | Low precision | Low ROB | Low ROB | Unclear |
|  | 2002-4 | 35. 7[[46](#_ENREF_46)] | Good precision | Low ROB | High ROB | Unclear |
|  | 2002 | 0.7 [[47](#_ENREF_47)] | Good precision | Low ROB | Low ROB | Unclear |
|  | 2001-2 | 7.8 [[48](#_ENREF_48)] | Low precision | Low ROB | High ROB | Unclear |
|  | 2001-2 | 17.0 [[48](#_ENREF_48)] | Good precision | Low ROB | Low ROB | Unclear |
|  | 2001-6 | 1.6 [[49](#_ENREF_49)] | Good precision | Low ROB | High ROB | Unclear |
|  | 2001-6 | 12.7 [[50](#_ENREF_50)] | Good precision | Low ROB | High ROB | Unclear |
|  | 2001-3 | 67.5 [[51](#_ENREF_51)] | Good precision | Low ROB | High ROB | Unclear |
|  | 2001-2 | 0.0 [[52](#_ENREF_52)] | Good precision | Low ROB | High ROB | Unclear |
|  | 2001-2 | 6.9 [[52](#_ENREF_52)] | Good precision | Low ROB | High ROB | Low ROB |
|  | 2001 | 19.2 [[53](#_ENREF_53)] | Good precision | Low ROB | High ROB | Unclear |
|  | 2000-5 | 25.8 [[54](#_ENREF_54)] | Low precision | Low ROB | High ROB | Unclear |
|  | 1998 | 1.2 [[55](#_ENREF_55)] | Good precision | Low ROB | High ROB | Unclear |
|  | 1996 | 0.0 [[56](#_ENREF_56)] | Good precision | Low ROB | Low ROB | Unclear |
|  |  | 41.7 [[57](#_ENREF_57)] | Good precision | Low ROB | High ROB | Unclear |
|  | -- | 0.0 [[58](#_ENREF_58)] | Low precision | Low ROB | Low ROB | Unclear |
|  | -- | 20.5 [[59](#_ENREF_59)] | Good precision | Low ROB | Low ROB | Unclear |
|  | -- | 8.8 [[60](#_ENREF_60)] | Low precision | Low ROB | High ROB | Unclear |
| **Jordan** | 2009 | 0. 0 [[61](#_ENREF_61)] | Good precision | Low ROB | Low ROB | Unclear |
|  | 2009 | 0.0 [[61](#_ENREF_61)] | Low precision | Low ROB | Low ROB | Unclear |
|  | 2009 | 0.0 [[61](#_ENREF_61)] | Low precision | Low ROB | Low ROB | Unclear |
| **Lebanon** | 2007-8 | 0.0 [[62](#_ENREF_62)] | Low precision | Low ROB | Low ROB | High ROB |
|  | 2000-2 | 0.0 [[63](#_ENREF_63)] | Low precision | Low ROB | High ROB | Unclear |
| **Libya** | 2010 | 87.1 [[64](#_ENREF_64)] | Good precision | Low ROB | Low ROB | Low ROB |
| **Morocco** | 2011-12 | 25.1 [[65](#_ENREF_65)] | Good precision | Low ROB | Low ROB | Low ROB |
|  | 2010-11 | 0·4 [[65](#_ENREF_65)] | Good precision | Low ROB | Low ROB | Low ROB |
|  | 2008 | 0.0 [[66](#_ENREF_66)] | Unclear | Low ROB | Unclear | Unclear |
|  | 2008 | 37.8 [[66](#_ENREF_66)] | Good precision | Low ROB | Low ROB | Unclear |
|  | 1991-9 | 33.0 [[67](#_ENREF_67)] | Good precision | Low ROB | High ROB | Unclear |
| **Oman** | -- | 12.0 [[68](#_ENREF_68)] | Low precision | High ROB | High ROB | High ROB |
|  | -- | 27.0 [[68](#_ENREF_68)] | Low precision | High ROB | High ROB | High ROB |
|  | -- | 18.0 [[68](#_ENREF_68)] | Low precision | High ROB | High ROB | High ROB |
| **OPT** | 2010 | 0.0 [[69](#_ENREF_69)] | Good precision | Low ROB | Low ROB | Unclear |
| **Pakistan** | 2011 | 49.6 [[70](#_ENREF_70)] | Good precision | Low ROB | Low ROB | Low ROB |
|  | 2011 | 52.5 [[70](#_ENREF_70)] | Good precision | Low ROB | Low ROB | Low ROB |
|  | 2011 | 46.2 [[70](#_ENREF_70)] | Good precision | Low ROB | Low ROB | High ROB |
|  | 2011 | 30.8 [[70](#_ENREF_70)] | Good precision | Low ROB | Low ROB | Low ROB |
|  | 2011 | 24.9 [[70](#_ENREF_70)] | Good precision | Low ROB | Low ROB | Low ROB |
|  | 2011 | 3.3 [[70](#_ENREF_70)] | Good precision | Low ROB | Low ROB | Low ROB |
|  | 2011 | 14.9 [[70](#_ENREF_70)] | Good precision | Low ROB | Low ROB | High ROB |
|  | 2011 | 40.6 [[70](#_ENREF_70)] | Good precision | Low ROB | Low ROB | Low ROB |
|  | 2011 | 16.0 [[70](#_ENREF_70)] | Good precision | Low ROB | Low ROB | High ROB |
|  | 2011 | 42.2 [[70](#_ENREF_70)] | Good precision | Low ROB | Low ROB | Low ROB |
|  | 2011 | 18.6 [[70](#_ENREF_70)] | Good precision | Low ROB | Low ROB | Low ROB |
|  | 2011 | 19.2 [[70](#_ENREF_70)] | Good precision | Low ROB | Low ROB | Low ROB |
|  | 2011 | 7.9 [[70](#_ENREF_70)] | Low precision | Low ROB | Low ROB | High ROB |
|  | 2011 | 20.0 [[70](#_ENREF_70)] | Good precision | Low ROB | Low ROB | High ROB |
|  | 2011 | 7.1 [[70](#_ENREF_70)] | Good precision | Low ROB | Low ROB | Low ROB |
|  | 2011 | 21.4 [[70](#_ENREF_70)] | Good precision | Low ROB | Low ROB | Low ROB |
|  | 2009 | 8 [[71](#_ENREF_71)] | Good precision | Low ROB | High ROB | Low ROB |
|  | 2009 | 52 [[71](#_ENREF_71)] | Good precision | Low ROB | High ROB | Low ROB |
|  | 2009 | 23 [[71](#_ENREF_71)] | Good precision | Low ROB | High ROB | Low ROB |
|  | 2009 | 21 [[71](#_ENREF_71)] | Good precision | Low ROB | High ROB | Low ROB |
|  | 2008 | 13 [[72](#_ENREF_72)] | Good precision | Low ROB | High ROB | Low ROB |
|  | 2008 | 10 [[72](#_ENREF_72)] | Good precision | Low ROB | High ROB | Low ROB |
|  | 2008 | 41 [[72](#_ENREF_72)] | Good precision | Low ROB | High ROB | Low ROB |
|  | 2008 | 18.6 [[73](#_ENREF_73)] | Good precision | Low ROB | Low ROB | Low ROB |
|  | 2008 | 12.3 [[73](#_ENREF_73)] | Good precision | Low ROB | Low ROB | Low ROB |
|  | 2008 | 30.5 [[73](#_ENREF_73)] | Good precision | Low ROB | Low ROB | Low ROB |
|  | 2008 | 23.1 [[73](#_ENREF_73)] | Good precision | Low ROB | Low ROB | Low ROB |
|  | 2008 | 14.5 [[73](#_ENREF_73)] | Good precision | Low ROB | Low ROB | Low ROB |
|  | 2008 | 28.5 [[73](#_ENREF_73)] | Good precision | Low ROB | Low ROB | Low ROB |
|  | 2008 | 12.8 [[73](#_ENREF_73)] | Good precision | Low ROB | Low ROB | High ROB |
|  | 2008 | 22.8 [[73](#_ENREF_73)] | Good precision | Low ROB | Low ROB | Low ROB |
|  | 2007 | 2.6 [[74](#_ENREF_74)] | Good precision | Low ROB | Low ROB | Unclear |
|  | 2007 | 0.0 [[74](#_ENREF_74)] | Good precision | Low ROB | Low ROB | Unclear |
|  | 2006-7 | 1.4 [[75](#_ENREF_75)] | Low precision | Low ROB | Low ROB | High ROB |
|  | 2006-7 | 13.3 [[75](#_ENREF_75)] | Good precision | Low ROB | Low ROB | Low ROB |
|  | 2006-7 | 1.0 [[75](#_ENREF_75)] | Good precision | Low ROB | Low ROB | Low ROB |
|  | 2006-7 | 29.8 [[75](#_ENREF_75)] | Good precision | Low ROB | Low ROB | Low ROB |
|  | 2006-7 | 30.1 [[75](#_ENREF_75)] | Good precision | Low ROB | Low ROB | Low ROB |
|  | 2006-7 | 6.5 [[75](#_ENREF_75)] | Good precision | Low ROB | Low ROB | Low ROB |
|  | 2006-7 | 16.5 [[75](#_ENREF_75)] | Good precision | Low ROB | Low ROB | Low ROB |
|  | 2006-7 | 0.0 [[75](#_ENREF_75)] | Good precision | Low ROB | Low ROB | Low ROB |
|  | 2006-7 | 2.2 [[75](#_ENREF_75)] | Good precision | Low ROB | Low ROB | High ROB |
|  | 2006-7 | 9.5 [[75](#_ENREF_75)] | Good precision | Low ROB | Low ROB | High ROB |
|  | 2006-7 | 51.3 [[75](#_ENREF_75)] | Good precision | Low ROB | Low ROB | Low ROB |
|  | 2006-7 | 5.3 [[75](#_ENREF_75)] | Good precision | Low ROB | Low ROB | Low ROB |
|  | 2005 | 0.0 [[76](#_ENREF_76)] | Unclear | Low ROB | High ROB | Unclear |
|  | 2005 | 9.5 [[77](#_ENREF_77)] | Good precision | Low ROB | Low ROB | Unclear |
|  | 2005 | 2.5 [[77](#_ENREF_77)] | Good precision | Low ROB | Low ROB | Unclear |
|  | 2005 | 12.0 [[77](#_ENREF_77)] | Good precision | Low ROB | Low ROB | Unclear |
|  | 2005 | 1.0 [[77](#_ENREF_77)] | Good precision | Low ROB | Low ROB | Unclear |
|  | 2005 | 13.3 [[78](#_ENREF_78)] | Good precision | Low ROB | Low ROB | Low ROB |
|  | 2005 | 25.3 [[78](#_ENREF_78)] | Good precision | Low ROB | Low ROB | Low ROB |
|  | 2005 | 3.8 [[78](#_ENREF_78)] | Good precision | Low ROB | Low ROB | Low ROB |
|  | 2005 | 0.3 [[78](#_ENREF_78)] | Good precision | Low ROB | Low ROB | Low ROB |
|  | 2005 | 0.4 [[78](#_ENREF_78)] | Good precision | Low ROB | Low ROB | High ROB |
|  | 2005 | 9.5 [[78](#_ENREF_78)] | Good precision | Low ROB | Low ROB | High ROB |
|  | 2005 | 19.2 [[78](#_ENREF_78)] | Good precision | Low ROB | Low ROB | Low ROB |
|  | 2004 | 23.1 [[79](#_ENREF_79)] | Good precision | Low ROB | Low ROB | Low ROB |
|  | 2004 | 0.5 [[79](#_ENREF_79)] | Good precision | Low ROB | Low ROB | Low ROB |
|  | 2004 | 24.0 [[80](#_ENREF_80)] | Low precision | Low ROB | High ROB | Unclear |
|  | 2004-5 | 26.0 [[81](#_ENREF_81)] | Good precision | Low ROB | Low ROB | Unclear |
|  | 2004-5 | 0.5 [[81](#_ENREF_81)] | Good precision | Low ROB | High ROB | Unclear |
|  | 2004 | 8.3 [[82](#_ENREF_82)] | Good precision | Low ROB | High ROB | Unclear |
|  | 2003 | 0.3 [[83](#_ENREF_83)] | Good precision | Low ROB | High ROB | Unclear |
|  | 2003 | 0.6 [[84](#_ENREF_84)] | Good precision | Low ROB | High ROB | Low ROB |
|  | 2003 | 0.0 [[85](#_ENREF_85)] | Good precision | Low ROB | High ROB | Unclear |
|  | 2003 | 0.0 [[85](#_ENREF_85)] | Low precision | Low ROB | High ROB | Unclear |
|  | 2003 | 9.7 [[86](#_ENREF_86)] | Good precision | Low ROB | High ROB | Unclear |
|  | 2002 | 0.0 [[87](#_ENREF_87)] | Low precision | Low ROB | High ROB | Unclear |
|  | 2002 | 3.4 [[88](#_ENREF_88)] | Good precision | Low ROB | High ROB | Unclear |
|  | 2002 | 0.0 [[89](#_ENREF_89)] | Good precision | Low ROB | High ROB | Unclear |
|  | 1999 | 0.0 [[90](#_ENREF_90)] | Good precision | Low ROB | High ROB | Unclear |
|  | 1996 | 0.4 [[91](#_ENREF_91)] | Good precision | Low ROB | High ROB | High ROB |
|  | 1994 | 0.0 [[92](#_ENREF_92)] | Good precision | Low ROB | High ROB | Unclear |
|  | 1987-4 | 0.0 [[93](#_ENREF_93)] | Low precision | Low ROB | High ROB | Unclear |
|  | -- | 37.2 [[94](#_ENREF_94)] | Low precision | Low ROB | High ROB | Unclear |
|  | -- | 0.0 [[95](#_ENREF_95)] | Good precision | Low ROB | Unclear | Unclear |
| **Syria** | 2006 | 0. 5 [[96](#_ENREF_96)] | Good precision | Low ROB | High ROB | Unclear |
| **Tunisia** | 2011 | 2.9 [[97](#_ENREF_97)] | Good precision | Low ROB | Low ROB | Low ROB |
|  | 2011 | 0.0 [[97](#_ENREF_97)] | Good precision | Low ROB | Low ROB | High ROB |
|  | 2009 | 3.1 [[98](#_ENREF_98)] | Good precision | Low ROB | Low ROB | Unclear |

OPT: Occupied Palestinian Territories, ROB: Risk of Bias

**References**

1. Afghanistan National AIDS Control Program (2012) Integrated Behavioral & Biological Surveillance (IBBS) in selected cities of Afghanistan: Findings of 2012 IBBS survey and comparison to 2009 IBBS survey. Johns Hopkins University School of Public Health, National AIDS Control Program, Ministry of Public Health. Kabul, Afghanistan.

2. Afghanistan National AIDS Control Program (2010) Integrated Behavioral & Biological Surveillance (IBBS) in Afghanistan: Year 1 Report. HIV Surveillance Project - Johns Hopkins University School of Public Health, National AIDS Control Program, Ministry of Public Health. Kabul, Afghanistan.

3. Todd CS, Nasir A, Stanekzai MR, Fiekert K, Rasuli MZ, et al. (2011) Prevalence and correlates of HIV, syphilis, and hepatitis B and C infection and harm reduction program use among male injecting drug users in Kabul, Afghanistan: A cross-sectional assessment. Harm Reduct J 8: 22.

4. Nasir A, Todd CS, Stanekzai MR, Bautista CT, Botros BA, et al. (2011) Prevalence of HIV, hepatitis B and hepatitis C and associated risk behaviours amongst injecting drug users in three Afghan cities. Int J Drug Policy 22: 145-152.

5. Todd CS, Abed AM, Strathdee SA, Scott PT, Botros BA, et al. (2007) HIV, hepatitis C, and hepatitis B infections and associated risk behavior in injection drug users, Kabul, Afghanistan. Emerg Infect Dis 13: 1327-1331.

6. Al-Haddad MK, Khashaba AS, Baig BZ, Khalfan S (1994) HIV antibodies among intravenous drug users in Bahrain. J Commun Dis 26: 127-132.

7. Family Health International and Ministry of Health Egypt (2010) HIV/AIDS Biological & Behavioral Surveillance Survey: Round Two Summary Report, Cairo, Egypt 2010. FHI in collaboration with the Ministry of Health and support from the Global Fund. Found at <http://www.fhi360.org/sites/default/files/media/documents/BBSS%202010_0.pdf>, Last accessed February 2014.

8. E. Elghamrawy, O. Abaza, S. Abou Elmagd, H. Ramy, S. Atallah, et al. Risk behaviours among male injecting drug users in Egypt. Abstract no. MOPE227 2012; Washington DC, USA.

9. Soliman C, Rahman IA, Shawky S, Bahaa T, Elkamhawi S, et al. (2010) HIV prevalence and risk behaviors of male injection drug users in Cairo, Egypt. AIDS 24 Suppl 2: S33-38.

10. Saleh E, El-Ghazzawi E, El-Sherbini I, Drew W, McFarland W, et al. (1998) Sentinel surveillance for HIV and high risk behaviors among injection drug users in Alexandria, Egypt. Abstract no. 13124. AIDS 1998 - XII International AIDS Conference. Geneva, Switzerland.

11. El-Ghazzawi E, Hunsmann G, Schneider J (1987) Low prevalence of antibodies to HIV-1 and HTLV-I in Alexandria, Egypt. AIDS Forsch 2: 639.

12. Hasan M, Farag A, Ismail M (1994) AIDS and intravenous drug users in Egypt. Abstract no. PC0143. AIDS 1994 - X International AIDS Conference Yokohama, Japan.

13. Attia, Medhat S (1996) HIV Seropositivity and KAP towards AIDS among drug addicts in Alexandria. Bull High Inst Public Health 26: 1-8.

14. Honarvar B, Odoomi N, Moghadami M, Afsar Kazerooni P, Hassanabadi A, et al. (2013) Blood-borne hepatitis in opiate users in iran: a poor outlook and urgent need to change nationwide screening policy. PLoS One 8: e82230.

15. Mehrjerdi ZA, Abarashi Z, Noroozi A, Arshad L, Zarghami M (2013) Correlates of shared methamphetamine injection among methamphetamine-injecting treatment seekers: the first report from Iran. Int J STD AIDS.

16. Iran Ministry of Public Health (2010) HIV bio-behavioral surveillance survey among injecting drug users in the Islamic Repubic of Iran. Final report [Persian], Tehran, Iran.

17. Alipour A, Haghdoost AA, Sajadi L, Zolala F (2013) HIV prevalence and related risk behaviours among female partners of male injecting drugs users in Iran: results of a bio-behavioural survey, 2010. Sex Transm Infect 89 Suppl 3: iii41-44.

18. Ilami O, Sarkari B, Khosravani A, Akbartabar Tori M, Hosseini Z (2012) HIV Seroprevalence among High-Risk Groups in Kohgiloyeh and Boyerahmad Province, Southwest of Iran, a Behavioral Surveillance Survey. AIDS Behav 16: 86-90.

19. Hashemipour M, Nokhodian Z, Yaran M, Ataei B, Tayeri K, et al. (2013) Prevalence of HIV infection in individuals with history of intravenous drug use via community announcement in Isfahan-Iran. Pakistan Journal of Medical Sciences 29: 395-398.

20. Javadi A, Ataei B, Yaran M, Nokhodian Z, Kassaian N, et al. (2013) Prevalence of HIV infection and related risk factors in Isfahan Drop in Centers. Pakistan Journal of Medical Sciences 29: 346-350.

21. Dibaj R, Ataei B, Yaran M, Nokhodian Z, Tayeri K, et al. (2013) Prevalence of HIV infection in inmates with history of injection drug use and evaluation of risk factors, in Isfahan, Iran. Pakistan Journal of Medical Sciences 29: 399-402.

22. Eskandarieh S, Nikfarjam A, Tarjoman T, Nasehi A, Jafari F, et al. (2013) Descriptive Aspects of Injection Drug Users in Iran's National Harm Reduction Program by Methadone Maintenance Treatment. Iran J Public Health 42: 588-593.

23. Zamani S, Radfar R, Nematollahi P, Fadaie R, Meshkati M, et al. (2010) Prevalence of HIV/HCV/HBV infections and drug-related risk behaviours amongst IDUs recruited through peer-driven sampling in Iran. Int J Drug Policy 21: 493-500.

24. Zadeh AOT, SeyedAlinaghi S, Hassanzad FF, Hajizadeh M, Mohamadi S, et al. (2014) Prevalence of HIV infection and the correlates among homeless in Tehran, Iran. Asian Pacific Journal of Tropical Biomedicine 4: 65-68.

25. Alinaghi SAS, Zadeh AOT, Zaresefat H, Hajizadeh M, Mohamadi SN, et al. (2013) Prevalence of HIV infection and the correlates among beggars in Tehran, Iran. Asian Pacific Journal of Tropical Disease 3: 76-78.

26. Ghasemian R, Najafi N, Amirkhanloo K (2011) The study of infections due to injection drug abuse in the injecting drug users hospitalized at Imam Khomeini Hospital in Sari and Razi Hospital in Ghaemshahr in 2007-2009. Journal of Mazandaran University of Medical Sciences 21: 8-15.

27. Kazerooni PA, Lari MA, Joolaei H, Parsa N (2010) Knowledge and attitude of male intravenous drug users on HIV/AIDS associated high risk behaviors in Shiraz Pir-Banon jail, Fars Province, Southern Iran. Iranian Red Crescent Medical Journal 12: 334-336.

28. Aminzadeh Z (2007) Seroepidemiology of HIV, syphilis, hepatitis B and C in intravenous drug users at Loghman Hakim hospital]. Iran J Med Meicrobiol 1: 53-56.

29. Rahimi-Movaghar A, Razaghi EM, Sahimi-Izadian E, Amin-Esmaeili M (2010) HIV, hepatitis C virus, and hepatitis B virus co-infections among injecting drug users in Tehran, Iran. Int J Infect Dis 14: e28-33.

30. Kheirandish P, Seyedalinaghi SA, Hosseini M, Jahani MR, Shirzad H, et al. (2010) Prevalence and correlates of HIV infection among male injection drug users in detention in Tehran, Iran. J Acquir Immune Defic Syndr 53: 273-275.

31. Iran Ministry of Health and Medical Education , Kyoto University School of Public Hleath (Japan) (2008) Integrated bio-behavioral surveillance for HIV infection among injecting drug users in Iran. Draft of the 1st analysis on the collected data, Tehran, Iran.

32. Malekinejad M, Mohraz M, Razani N, Khairandish P, McFarland W, et al. HIV and related risk behaviors of injecting drug users (IDU) in Iran: findings from the first respondent-driven sampling (RDS) survey of IDU in Tehran in 2006-2007. Abstract no. THAC0202 2008; Mexico.

33. Alavi SM, Nadimi M, Shokri S, Zamani G (2012) Seroepidemiology of human immunodeficiency virus in Illicit substance users in Ahvaz, Iran: 2005-2006. Jundishapur Journal of Microbiology 5: 474-478.

34. Tofigi H, Ghorbani M, Akhlaghi M, Yaghmaei A, Mostafazadeh B, et al. (2011) Incidence of hepatitis B and HIV virus at cadaver of IV drug abusers in Tehran. Acta Med Iran 49: 59-63.

35. Imani R, Karimi A, Rouzbahani R, Rouzbahani A (2008) Seroprevalence of HBV, HCV and HIV infection among intravenous drug users in Shahr-e-Kord, Islamic Republic of Iran. East Mediterr Health J 14: 1136-1141.

36. Mojtahedzadeh V, Razani N, Malekinejad M, Vazirian M, Shoaee S, et al. (2008) Injection drug use in Rural Iran: integrating HIV prevention into iran's rural primary health care system. AIDS Behav 12: S7-12.

37. Zamani S, Kihara M, Gouya MM, Vazirian M, Nassirimanesh B, et al. (2006) High prevalence of HIV infection associated with incarceration among community-based injecting drug users in Tehran, Iran. J Acquir Immune Defic Syndr 42: 342-346.

38. Shamaei M, Marjani M, Baghaei P, Chitsaz E, Rezaei Tabar E, et al. (2009) Drug abuse profile - patient delay, diagnosis delay and drug resistance pattern - among addict patients with tuberculosis. Int J STD AIDS 20: 320-323.

39. Pourahmad M, Javady A, Karimi I, Ataei B, Kassaeian N (2007) Seroprevalence of and risk factors associated with hepatitis B, hepatitis C, and human immunodeficiency virus among prisoners in Iran. Infectious Diseases in Clinical Practice 15: 368-372.

40. Zamani S, Kihara M, Gouya MM, Vazirian M, Ono-Kihara M, et al. (2005) Prevalence of and factors associated with HIV-1 infection among drug users visiting treatment centers in Tehran, Iran. AIDS 19: 709-716.

41. Farhoudi B, Montevalian A, Motamedi M, Khameneh MM, Mohraz M, et al. (2003) Human immunodeficiency virus and HIV - associated tuberculosis infection and their risk factors in injecting drug users in prison in Iran. Iran Ministry of Health, Tehran, Iran.

42. Khodadadizadeh A, Esmaeili Nadimi A, Hossieni SH, Shabani Sharbabaki Z (2006) The prevalence of HIV, HBV and HCV in narcotic addicted persons referred to the out patient clinic of rafsanjan university of medical sciences in 2003 [Persian]. J Rafsanjan Univ Med Sci 5: 21-30.

43. Alavi SM, Behdad F (2010) Seroprevalence study of hepatitis C and hepatitis B virus among hospitalized intravenous drug users in Ahvaz, Iran (2002-2006). Hepatitis Monthly 10: 101-104.

44. Davoodian P, Dadvand H, Mahoori K, Amoozandeh A, Salavati A (2009) Prevalence of selected sexually and blood-borne infections in Injecting drug abuser inmates of bandar abbas and roodan correction facilities, Iran, 2002. Braz J Infect Dis 13: 356-358.

45. Behnaz K, Abdollah A, Fateme F, Mohammadreza R (2007) Prevalence and risk factors of HIV, hepatitis B virus and hepatitis C virus infections in drug addicts among Gorgan prisoners. Journal of Medical Sciences 7: 252-254.

46. Asadi S, Marjani M (2006) Prevalence of intravenous drug use-associated infections. Iranian Journal of Clinical Infectious Diseases 1: 59-62.

47. Alizadeh AH, Alavian SM, Jafari K, Yazdi N (2005) Prevalence of hepatitis C virus infection and its related risk factors in drug abuser prisoners in Hamedan--Iran. World J Gastroenterol 11: 4085-4089.

48. Mir-Nasseri MM, Mohammadkhani A, Tavakkoli H, Ansari E, Poustchi H (2011) Incarceration is a major risk factor for blood-borne infection among intravenous drug users. Hepatitis Monthly 11: 19-22.

49. Sharif M, Sherif A, Sayyah M (2009) Frequency of HBV, HCV and HIV infections among hospitalized injecting drug users in Kashan. Indian J Sex Transm Dis 30: 28-30.

50. Alavi SM, Alavi L (2009) Seroprevalence study of HCV among hospitalized intravenous drug users in Ahvaz, Iran (2001-2006). J Infect Public Health 2: 47-51.

51. Alavi SM, Etemadi A (2007) HIV/HBV, HIV/HCV and HIV/HTLV-1 co infection among injecting drug user patients hospitalized at the infectious disease ward of a training hospital in Iran. Pakistan Journal of Medical Sciences 23: 510-513.

52. Rahbar AR, Rooholamini S, Khoshnood K (2004) Prevalence of HIV infection and other blood-borne infections in incarcerated and non-incarcerated injection drug users (IDUs) in Mashhad, Iran. International Journal of Drug Policy 15: 151-155.

53. Alaei K, Alaei A, Mansoori D, Tabar, Heravi, et al. (2002) The epidemiological status of IDU and HIV infection in addict cases submitted to HIV/STI/IDU Counseling and Care Center in Kermanshah province. Abstract no. LbPp2211. AIDS 2002 - XIV International AIDS Conference. Barcelona, Spain.

54. Sharifi-Mood B, Metanat M (2006) Infection among hospitalized injection drug users. Journal of Medical Sciences 6: 686-689.

55. Mirahmadizadeh A, Kadivar M, Hemmati A, Javadi A (2004) Infection with HIV and hepatitis C and B viruses among injecting drug users in Shiraz, Southern Iran. Abstract no. WePeC5981. AIDS 2004 - XV International AIDS Conference. Bangkok, Thailand.

56. Nowroozi A, Zali M, Gooya M, Kowsarian P, Raoufi M (1998) The simultanity of HIV, and HBV, HCV and syphilis among the addicted prisoners. Abstract no. 60835. AIDS 1998 - XII International AIDS Conference. Geneva, Switzerland.

57. Alavian SM, Mirahmadizadeh A, Javanbakht M, Keshtkaran A, Heidari A, et al. (2013) Effectiveness of methadone maintenance treatment in prevention of hepatitis C virus transmission among injecting drug users. Hepatitis Monthly 13: 9.

58. Azarkar Z, Sharifzadeh G (2010) Evaluation of the prevalence of Hepatitis B, Hepatitis C, and HIV in inmates with drug-related convictions in Birjand, Iran in 2008. Hepatitis Monthly 10: 26-30.

59. Mirahmadizadeh AR, Majdzadeh R, Mohammad K, MH F (2009) Prevalence of HIV and Hepatitis C Virus Infections and Related Behavioral Determinants among Injecting Drug Users of Drop-in Centers in Iran. Iranian Red Crescent Medical Journal 11: 325-329.

60. Amini S, Mahmoodabadi SA, Lamian S, Joulaie M, Farahani MM (2005) Prevalence of hepatitis G virus (HGV) in high-risk groups and blood donors in Tehran, Iran. Iranian Journal of Public Health 34: 41-46.

61. Jordan National AIDS Program (2010) Preliminary analysis of Jordan IBBSS among injecting drug users. Ministry of Health, Amman, Jordan.

62. Mahfoud Z, Afifi R, Ramia S, El Khoury D, Kassak K, et al. (2010) HIV/AIDS among female sex workers, injecting drug users and men who have sex with men in Lebanon: results of the first biobehavioral surveys. AIDS 24 Suppl 2: S45-54.

63. Ramia S, Klayme S, Naman R (2003) Infection with hepatitis B and C viruses and human retroviruses (HTLV-I and HIV) among high-risk Lebanese patients. Ann Trop Med Parasitol 97: 187-192.

64. Mirzoyan L, Berendes S, Jeffery C, Thomson J, Ben Othman H, et al. (2013) New evidence on the HIV epidemic in Libya: why countries must implement prevention programs among people who inject drugs. J Acquir Immune Defic Syndr 62: 577-583.

65. Morocco Ministry of Health, National Aids Control Program, National Institute of Hygiene, UNAIDS, Global Fund to Fight AIDS Tuberculosis and Malaria (2012) HIV Integrated Behavioral and Biological Surveillance Surveys-Morocco 2011-2012: Injecting Drug Users in Tanger and Nador, Morocco. Rabat, Morocco.

66. Morocco Ministry of Health (February 2010) Situation épidémiologique du VIH/Sida et des IST au Maroc [French]. Epidemiological assessment of HIV/AIDS and STIs in Morocco. Rabat, Morocco.

67. Elharti E (2002) HIV epidemiology in Morocco: a nine-year survey (1991–1999). ElHarti E, personal communication, updated data up to 2008. International journal of STD and AIDS 13: 839-842.

68. Oman Ministry of Health (2006) HIV Risk among Heroin and Injecting Drug Users in Muscat, Oman. Quantitative Survey. Preliminary Data. Muscat, Oman.

69. Stulhofer A, Chetty A, Rabie RA, Jwehan I, Ramlawi A (2012) The Prevalence of HIV, HBV, HCV, and HIV-Related Risk-Taking Behaviors among Palestinian Injecting Drug Users in the East Jerusalem Governorate. J Urban Health 89: 671-676.

70. Pakistan National AIDS Control Program (2011) HIV Second Generation Surveillance In Pakistan. National Report Round IV. Canada-Pakistan HIV/AIDS Surveillance Project. National Aids Control Program, Ministry Of Health, Pakistan. Found at <http://www.nacp.gov.pk/library/reports/Surveillance%20&%20Research/HIV-AIDS%20Surveillance%20Project-HASP/HIV%20Second%20Generation%20Surveillance%20in%20Pakistan%20-%20National%20report%20Round%20IV%202011.pdf>, Last accessed February 2014.

71. Nai Zindagi, Punjab Provincial AIDS Control Program (2009) Rapid situation assessments of HIV prevalence and risk factors among people injecting drugs in four cities of the Punjab.

72. Nai Zindagi, Punjab Provincial AIDS Control Program (2008) The hidden truth: A study of HIV vulnerability, risk factors and prevalence among men injecting drugs and their wives.

73. Pakistan National AIDS Control Program (2008) HIV Second Generation Surveillance In Pakistan. National Report Round III. Canada-Pakistan HIV/AIDS Surveillance Project. National Aids Control Program, Ministry Of Health, Pakistan. Found at <http://www.nacp.gov.pk/library/reports/Surveillance%20&%20Research/HIV-AIDS%20Surveillance%20Project-HASP/HIV%20Second%20Generation%20Surveillance%20in%20Pakistan%20-%20National%20report%20Round%20III%202008.pdf>, Last accessed February 2014.

74. Platt L, Vickerman P, Collumbien M, Hasan S, Lalji N, et al. (2009) Prevalence of HIV, HCV and sexually transmitted infections among injecting drug users in Rawalpindi and Abbottabad, Pakistan: evidence for an emerging injection-related HIV epidemic. Sex Transm Infect 85 Suppl 2: ii17-22.

75. Pakistan National AIDS Control Program (2006-07) HIV Second Generation Surveillance In Pakistan. National Report Round II. Canada-Pakistan HIV/AIDS Surveillance Project. National Aids Control Program, Ministry Of Health, Pakistan. Found at <http://www.nacp.gov.pk/library/reports/Surveillance%20&%20Research/HIV-AIDS%20Surveillance%20Project-HASP/HIV%20Second%20Generation%20Surveillance%20in%20Pakistan%20-%20Round%202%20Report%202006-07.pdf>. Last accessed February 2014.

76. Rahman K, Akhtar A, Aslam M (2006) Seroprevalence of HIV, HBV & HCV in drug users in Pakistan. Abstract no. CDC0097. AIDS 2006 - XVI International AIDS Conference Toronto, Canada.

77. Nai Zindagi, Punjab Provincial AIDS Control Program (2005) The lethal overdose: Injecting drug use and HIV/AIDS.

78. Pakistan National AIDS Control Program (2005) HIV Second Generation Surveillance In Pakistan. National Report Round I. Canada-Pakistan HIV/AIDS Surveillance Project. National Aids Control Program, Ministry Of Health, Pakistan. Found at <http://www.nacp.gov.pk/library/reports/Surveillance%20&%20Research/HIV-AIDS%20Surveillance%20Project-HASP/HIV%20Second%20Generation%20Surveillance%20in%20Pakistan%20-%20Round%201%20Report%20-%202005.pdf>, Last accessed February 2014.

79. Bokhari A, Nizamani NM, Jackson DJ, Rehan NE, Rahman M, et al. (2007) HIV risk in Karachi and Lahore, Pakistan: an emerging epidemic in injecting and commercial sex networks. Int J STD AIDS 18: 486-492.

80. Achakzai M, Kassi M, Kasi PM (2007) Seroprevalences and co-infections of HIV, hepatitis C virus and hepatitis B virus in injecting drug users in Quetta, Pakistan. Trop Doct 37: 43-45.

81. Bokhari A, Emmanuel F, Abbas S (2006) Integrated biological & behavioral surveillance: results of pilot studies in Pakistan. Abstract no. MOPE0552 AIDS 2006 - XVI International AIDS Conference. Toronto, Canada.

82. Abbasi B, Somroo F (2005) HIV outbreak among injecting drug users in larkana, pakistan a serious threat for generalized epidemic. Abstract no. WeOa0305. IAS 2005 - The 3rd IAS Conference on HIV Pathogenesis and Treatment. Rio de Janeiro, Brazil.

83. Abbasi S, Faqir F, Khan S, Zaidi SK, Ahmed SQ, et al. (2009) A serological study of hepatitis C and human immunodeficiency virus in a cohort of intravenous drug users in Quetta, Balochistan. Journal of Postgraduate Medical Institute 23: 3-6.

84. Altaf A, Shah SA, Zaidi NA, Memon A, Nadeem ur R, et al. (2007) High risk behaviors of injection drug users registered with harm reduction programme in Karachi, Pakistan. Harm Reduct J 4: 7.

85. Kuo I, ul-Hasan S, Galai N, Thomas DL, Zafar T, et al. (2006) High HCV seroprevalence and HIV drug use risk behaviors among injection drug users in Pakistan. Harm Reduct J 3: 26.

86. Shah SA, Altaf A, Mujeeb SA, Memon A (2004) An outbreak of HIV infection among injection drug users in a small town in Pakistan: potential for national implications. Int J STD AIDS 15: 209.

87. Akhtar A, Aslam M, Zafar M (2004) Determinants of HIV risky behaviors among drug users in Faisalabad Pakistan. Abstract no. C11530. AIDS 2004 - XV International AIDS Conference Bangkok, Thailand.

88. Hadi D.H.M.H, Shujaat P.D.M.G.S.H, Waheed P.D.W.u.Z, Masood P.D.M.G.M.A (2005) Incidence of hepatitis C virus and HIV among injecting drug users in Northern Pakistan: a prospective cohort study. Abstract no. MoOa0104. IAS 2005 - The 3rd IAS Conference on HIV Pathogenesis and Treatment Rio de Janeiro, Brazil.

89. Altaf A, Shah SA, A. M (2003) Follow up study to assess and evaluate knowledge, attitude and high risk behaviors and prevalence of HIV, HBV, HCV and Syphilis among IDUS at Burns Road DIC, Karachi. External report submitted to UNODC.

90. Nai Zindagi, UNODCCP, UNAIDS. (1999) Baseline study of the relationship between injecting drug use, HIV and Hepatitis C among male injecting drug users in Lahore.

91. Parviz S, Fatmi Z, Altaf A, McCormick JB, Fischer-Hoch S, et al. (2006) Background demographics and risk behaviors of injecting drug users in Karachi, Pakistan. Int J Infect Dis 10: 364-371.

92. Baqi S, Nabi N, Hasan SN, Khan AJ, Pasha O, et al. (1998) HIV antibody seroprevalence and associated risk factors in sex workers, drug users, and prisoners in Sindh, Pakistan. J Acquir Immune Defic Syndr Hum Retrovirol 18: 73-79.

93. Iqbal J, Rehan N (1996) Sero-prevalence of HIV: six years' experience at Shaikh Zayed Hospital, Lahore. J Pak Med Assoc 46: 255-258.

94. Khanani MR, Ansari AS, Khan S, Somani M, Kazmi SU, et al. (2010) Concentrated epidemics of HIV, HCV, and HBV among Afghan refugees. Journal of Infection 61: 434-437.

95. UrRehman N (2002) Injecting drug use and HIV/AIDS in pakistan. Abstract no. MoPeD3667. AIDS 2002 - XIV International AIDS Conference. Barcelona, Spain.

96. Syria Mental Health Directorate, Syria National AIDS Programme (2008) Assessment of HIV Risk and Sero-prevalence among Drug Users in Greater Damascus. Syrian Ministry of Health. UNODC. UNAIDS. Damascus, Syria

97. Tunisia Ministry of Health, Tunisian Association for Information and Orientation on HIV (2013) Enquête sérocomportementale du VIH et des hépatites virales C auprès des usagers de drogues injectables en Tunisie [French]. Biobehavioral surveillance of HIV and Hepatitis C among injecting drug users in Tunisia. Tunis, Tunisia.

98. Tunisia Ministry of Health (2010) Synthèse des enquêtes de séroprévalence et sérocomportementales auprès de trois populations à vulnérables au VIH : Les usagers de drogues injectables, les hommes ayant des rapports sexuels avec des hommes et les travailleuses du sexe clandestines en Tunisie [French]. Synthesis of biobehavioral surveillance among the three populations vulnerable to HIV in Tunisia: Injecting drug users, men who have sex with men, and female sex workers. Tunis, Tunisia.
