# Supplementary material for: HIV among People Who Inject Drugs in the Middle East and North Africa: Systematic Review and Data Synthesis
Source: PLoS Med. 2014 Jun 17;11(6):e1001663. doi: 10.1371/journal.pmed.1001663 (PMC4061009; doi:10.1371/journal.pmed.1001663)
Supplement: Table S2 — Summary of precision and risk of bias of HIV prevalence measures as extracted from eligible reports. (DOCX) [file pmed.1001663.s002.docx]

**Table S2. Summary of precision and risk of bias of HIV prevalence measures as extracted from eligible reports**

|  | **n** | **%^*^** |
| --- | --- | --- |
| **Precision of estimates** |  |  |
| High precision | 159 | 83.7 |
| Low precision | 29 | 15.3 |
| Missing | 2 | 1.1 |
| **Risk of bias quality domains** |  |  |
| **HIV ascertainment** |  |  |
| Low risk of bias | 186 | 97.9 |
| High risk of bias | 4 | 2.1 |
| Unclear | -- | -- |
| **Sampling methodology** |  |  |
| Low risk of bias | 100 | 52.6 |
| High risk of bias | 88 | 46.3 |
| Unclear | 2 | 1.1 |
| **Response rate** |  |  |
| Low risk of bias | 64 | 33.7 |
| High risk of bias | 20 | 10.5 |
| Unclear | 106 | 55.8 |
| **Total** | 190**^*^** | 100.0 |
|  |  |  |
|  | **n** | **%** |
| **Low risk of bias** |  |  |
| In at least one quality domain | 186 | 97.9 |
| In at least two quality domains | 117 | 61.6 |
| In all three quality domains | 47 | 24.7 |
| **High risk of bias** |  |  |
| In at least one quality domain | 104 | 54.7 |
| In at least two quality domains | 5 | 2.6 |
| In all three quality domains | 3 | 1.6 |

**^*^** Out of a total of 190 HIV prevalence measures among predominantly male PWID
