# Supplementary material for: HIV among People Who Inject Drugs in the Middle East and North Africa: Systematic Review and Data Synthesis
Source: PLoS Med. 2014 Jun 17;11(6):e1001663. doi: 10.1371/journal.pmed.1001663 (PMC4061009; doi:10.1371/journal.pmed.1001663)
Supplement: Table S3 — Subnational estimates of the number and prevalence of people who inject drugs in the Middle East and North Africa. (DOCX) [file pmed.1001663.s003.docx]

Table S3. Subnational estimates of the number and prevalence of people who inject drugs in the Middle East and North Africa

| **Country** | **City/province** | **Year** | **N (range)** | **% (range)** | **Source** |
| --- | --- | --- | --- | --- | --- |
| **Afghanistan** | Mazar-i-Sharif, Kabul,  Jalalabad | 2006-7 | 1,465 (55-1,251) | 0.22 (0.15-0.24) | [[1](#_ENREF_1)] |
|  | Herat | 2012 | 1,211 |  | [[2](#_ENREF_2)] |
|  | Jalalabad | 2012 | 1,471 |  | [[2](#_ENREF_2)] |
|  | Kabul | 2012 | 12,541 |  | [[2](#_ENREF_2)] |
|  | Mazar-i-Sharif | 2012 | 1,496 |  | [[2](#_ENREF_2)] |
| **Egypt** | Greater Cairo | -- | 85,000 |  | [[3](#_ENREF_3)] |
| **Iran** | Hamadan | 2012 | 11,333 |  | [[4](#_ENREF_4)] |
|  | Hormozgan | 2006 |  | 0.1 | [[5](#_ENREF_5)] |
|  | Kerman city | -- | 1,640 (1,368-1,911) |  | [[6](#_ENREF_6)] |
|  | Kerman city | -- | 3,805 (57-11,254) |  | [[6](#_ENREF_6)] |
|  | Kermanshah | 2006 |  | 0.7 | [[7](#_ENREF_7)] |
|  | Khoshropdpey | 2003 |  | 0.0 | [[8](#_ENREF_8)] |
|  | Tehran | 2006 |  | 1.0 | [[5](#_ENREF_5)] |
| **Pakistan** | 19 cities | 2011 | 46,351 (39,793-52,896) | 0.37 | [[9](#_ENREF_9)] |
|  | 8 cities | 2005 | 24,390 (20,770-28,010) | 0.47 | [[10](#_ENREF_10)] |
|  | Bannu | 2006 | 250 | 0.08 | [[11](#_ENREF_11)] |
|  | Faisalabad | 2005 | (2,400-2,550) |  | [[12](#_ENREF_12)] |
|  | Faisalabad | 2006 | 8030 | 1.07 | [[11](#_ENREF_11)] |
|  | Gujranwala | 2005 | (466-607) |  | [[12](#_ENREF_12)] |
|  | Gujranwala | 2006 | 2650 | 0.62 | [[11](#_ENREF_11)] |
|  | Hyderabad | 2006 | 2600 | 0.66 | [[11](#_ENREF_11)] |
|  | Islamabad | 2006 |  | 5.9 | [[13](#_ENREF_13)] |
|  | Karachi | 2006 | 9000 | 0.25 | [[11](#_ENREF_11)] |
|  | Kech | 2007 |  | 0.4 | [[14](#_ENREF_14)] |
|  | Lahore | 2005 | (1,754-2,110) |  | [[12](#_ENREF_12)] |
|  | Lahore | 2006 | 3350 | 0.18 | [[11](#_ENREF_11)] |
|  | Larkana | 2006 | 800 | 0.65 | [[11](#_ENREF_11)] |
|  | Mandi Bahauddin | 2005 | (713-928) |  | [[12](#_ENREF_12)] |
|  | Multan | 2006 | 900 | 0.21 | [[11](#_ENREF_11)] |
|  | Peshawar | 2006 | 150 | 0.04 | [[11](#_ENREF_11)] |
|  | Quetta | 2006 | 150 | 0.07 | [[11](#_ENREF_11)] |
|  | Rawalpindi | 2005 | (348-451) |  | [[12](#_ENREF_12)] |
|  | Rawalpindi | 2006 | 123 | 0.02 | [[11](#_ENREF_11)] |
|  | Sarghoda | 2005 | (1,000-1,100) |  | [[12](#_ENREF_12)] |
|  | Sarghoda | 2006 | 2450 | 0.87 | [[11](#_ENREF_11)] |
|  | Sheikhukupura | 2005 | (367-460) |  | [[12](#_ENREF_12)] |
|  | Sialkot | 2005 | (600-800) |  | [[12](#_ENREF_12)] |
|  | Sukkur | 2006 | 1350 | 0.59 | [[11](#_ENREF_11)] |
| **Tunisia** | Bizerte | 2112 | 654 |  | [[15](#_ENREF_15)] |
|  | Tunis | 2012 | 1,573 |  | [[15](#_ENREF_15)] |

**References**

1. SAR AIDS, The World Bank (2008) Mapping and Situation Assessment of Key Populations at High Risk of HIV in Three Cities of Afghanistan. Human Development Sector, South Asia Region, World Bank. Washington DC, USA.

2. Afghanistan National AIDS Control Program (2012) Integrated Behavioral & Biological Surveillance (IBBS) in selected cities of Afghanistan: Findings of 2012 IBBS survey and comparison to 2009 IBBS survey. Johns Hopkins University School of Public Health, National AIDS Control Program, Ministry of Public Health. Kabul, Afghanistan.

3. World Health Organization - Eastern Mediterranean Region (2013) HIV Surveillance Systems: Regional Update 2012,Cairo, Egypt.

4. Khazaei S, Poorolajal J, Mahjub H, Esmailnasab N, Mirzaei M (2012) Estimation of the Frequency of Intravenous Drug Users in Hamadan City, Iran, Using the Capture-recapture Method. Epidemiol Health 34: e2012006.

5. Merat S, Rezvan H, Nouraie M, Jafari E, Abolghasemi H, et al. (2010) Seroprevalence of hepatitis C virus: the first population-based study from Iran. Int J Infect Dis 14 Suppl 3: e113-116.

6. Shokoohi M, Baneshi MR, Haghdoost AA (2012) Size estimation of groups at high risk of HIV/AIDS using network scale up in Kerman, Iran. International Journal of Preventive Medicine 3: 471-476.

7. Sayad B, Saeed FS, Keyvani H, Rezali M, Asadi T, et al. (2008) Seroepidemiology of hepatitis C in Kermanshah (West of Iran, 2006). Hepatitis Monthly 8: 141-146.

8. Meysamie A, Sedaghat M, Mahmoodi M, Ghodsi SM, Eftekhar B (2009) Opium use in a rural area of the Islamic Republic of Iran. Eastern Mediterranean Health Journal 15: 425-431.

9. Pakistan National AIDS Control Program (2011) HIV Second Generation Surveillance In Pakistan: Mapping of key populations at risk of HIV infection. National Report Round IV. Canada-Pakistan HIV/AIDS Surveillance Project. National Aids Control Program, Ministry Of Health, Pakistan. Found at <http://www.nacp.gov.pk/library/reports/Surveillance%20&%20Research/HIV-AIDS%20Surveillance%20Project-HASP/HIV%20Second%20Generation%20Surveillance%20in%20Pakistan%20-%20Mapping%20Report%20Round%204%202011.pdf>, Last accessed February 2014.

10. Pakistan National AIDS Control Program (2005) HIV Second Generation Surveillance In Pakistan. National Report Round I. Canada-Pakistan HIV/AIDS Surveillance Project. National Aids Control Program, Ministry Of Health, Pakistan. Found at <http://www.nacp.gov.pk/library/reports/Surveillance%20&%20Research/HIV-AIDS%20Surveillance%20Project-HASP/HIV%20Second%20Generation%20Surveillance%20in%20Pakistan%20-%20Round%201%20Report%20-%202005.pdf>, Last accessed February 2014.

11. Emmanuel F, Blanchard J, Zaheer HA, Reza T, Holte-McKenzie M, et al. (2010) The HIV/AIDS Surveillance Project mapping approach: an innovative approach for mapping and size estimation for groups at a higher risk of HIV in Pakistan. AIDS 24 Suppl 2: S77-84.

12. Nai Zindagi, Punjab Provincial AIDS Control Program (2005) The lethal overdose: Injecting drug use and HIV/AIDS.

13. Hashmi A, Saleem K, Soomro JA (2010) Prevalence and factors associated with hepatitis C virus seropositivity in female individuals in islamabad, pakistan. Int J Prev Med 1: 252-256.

14. Ahmed F, Irving WL, Anwar M, Myles P, Neal KR (2011) Prevalence and risk factors for hepatitis C virus infection in Kech District, Balochistan, Pakistan: most infections remain unexplained. A cross-sectional study. Epidemiol Infect: 1-8.

15. Tunisia Ministry of Health, Tunisian Association for Information and Orientation on HIV (2013) Enquête sérocomportementale du VIH et des hépatites virales C auprès des usagers de drogues injectables en Tunisie [French]. Biobehavioral surveillance of HIV and Hepatitis C among injecting drug users in Tunisia. Tunis, Tunisia.
