# Supplementary material for: HIV among People Who Inject Drugs in the Middle East and North Africa: Systematic Review and Data Synthesis
Source: PLoS Med. 2014 Jun 17;11(6):e1001663. doi: 10.1371/journal.pmed.1001663 (PMC4061009; doi:10.1371/journal.pmed.1001663)
Supplement: Table S4 — HIV point-prevalence measures among people who inject drugs as extracted from various databases including the US Census Bureau database, the WHO/EMRO testing database, the UNAIDS epidemiological fact sheets databases, and other sources of data with unidentified reports. (DOCX) [file pmed.1001663.s004.docx]

**Table S4. HIV point-prevalence measures among people who inject drugs as extracted from various databases including the US Census Bureau database, the WHO/EMRO testing database, the UNAIDS epidemiological fact sheets databases, and other sources of data with unidentified reports**

|  | **Afg** | **Bah** | **Dji** | **Egy** | **Irn** | **Irq** | **Jor** | **Kuw** | **Leb** | **Lib** | **Mor** | **Oma** | **OPT** | **Pak** | **SA** | **Sud** | **Syr** | **Tun** | **Yem** |
| --- | --- | --- | --- | --- | --- | --- | --- | --- | --- | --- | --- | --- | --- | --- | --- | --- | --- | --- | --- |
| **Year** | n (%) | n (%) | n (%) | n (%) | n (%) | n (%) | n (%) | n (%) | n (%) | n (%) | n (%) | n (%) | n (%) | n (%) | n (%) | n (%) | n (%) | n (%) | n (%) |
| **1984** |  |  |  |  |  |  |  |  |  |  |  |  |  |  |  |  |  |  |  |
| **1987** |  |  |  |  |  |  |  |  |  |  |  |  |  |  |  |  |  |  |  |
| **1988** |  |  |  |  |  |  |  |  | 103 (3.9) |  |  |  |  |  |  |  | 51 (0.0) |  |  |
| **1989** |  | 728 (7.8) |  | 1932 (0.2) |  |  |  |  |  |  |  |  |  | 97 (0.0) |  |  |  |  |  |
| **1990** |  | 1436 (2.9) |  |  | 135 (0.0) |  | 240 (0.4) |  |  |  |  |  |  |  |  |  |  |  |  |
|  |  |  |  |  |  |  | 24 (4.2) |  |  |  |  |  |  |  |  |  |  |  |  |
| **1991** |  | 609 (2.1) |  | 122 (0.0) | 579 (0.2) |  |  | 840 (0.4) |  |  |  |  |  | 18 (0.0) |  |  |  |  |  |
|  |  |  |  | 72 (0.0) |  |  |  |  |  |  |  |  |  |  |  |  |  |  |  |
|  |  |  |  | 50 (0.0) |  |  |  |  |  |  |  |  |  |  |  |  |  |  |  |
| **1992** |  | 393 (1.0) |  | 266 (0.8) | 2098 (0.1) |  | 4 (100.0) |  |  |  |  |  |  | 24 (0.0) |  |  | 963 (0.1) | 801 (1.6) |  |
|  |  |  |  | 106 (2.8) |  |  |  |  |  |  |  |  |  |  |  |  |  |  |  |
|  |  |  |  | 4 (0.0) |  |  |  |  |  |  |  |  |  |  |  |  |  |  |  |
| **1993** |  | 139 (1.4) |  | 75 (1.3) | 1295 (0.2) | (0.0) | 26 (0.0) | 208 (1.0) | 46 (2.2) |  |  |  |  | 698 (0.3) |  |  | 157 (0.0) | 822 (1.1) |  |
|  |  |  |  | 616 (0.0) |  |  |  |  |  |  |  |  |  |  |  |  |  |  |  |
|  |  |  |  | 214 (0.0) |  |  |  |  |  |  |  |  |  |  |  |  |  |  |  |
| **1994** |  | 191 (1.6) |  | 385 (0.0) | 349 (0.3) | (0.0) |  | 59 (0.0) | 32 (3.1) |  |  |  |  | 342 (0.0) |  |  | 1419 (0.1) | 1224 (0.9) |  |
|  |  |  |  | 102 (0.0) |  |  |  |  |  |  |  |  |  |  |  |  | 1379 (0.3) |  |  |
| **1995** |  | 183 (0.0) |  | 1085 (0.0) | 1232 (0.0) | (0.0) |  | 110 (0.0) | 15 (0.0) |  |  |  |  | 703 (5.4) |  |  | 834 (0.0) | 1571 (0.7) |  |
|  |  |  |  |  |  |  |  |  |  |  |  |  |  |  |  |  | 165 (0.0) |  |  |
| **1996** |  | 439 (1.1) | (0.0) | 96 (0.0) | 2747 (5.7) | (0.0) |  | 172 (0.6) |  |  |  |  |  | 19 (0.0) |  |  | 153 (0.0) | 1518 (1.0) |  |
|  |  |  |  |  |  |  |  |  |  |  |  |  |  | 113 (1.8) |  |  |  |  |  |
|  |  |  |  |  |  |  |  |  |  |  |  |  |  | 242 (0.4) |  |  |  |  |  |
|  |  |  |  |  |  |  |  |  |  |  |  |  |  | 19 (0.0) |  |  |  |  |  |
| **1997** |  | 350 (0.0) |  | 138 (0.0) | 34120 (1.8) | (0.0) |  | 94 (0.0) |  |  |  |  |  | 32 (0.0) |  | (0.1) | 314 (0.0) | 584 (0.3) |  |
|  |  |  |  | 438 (0.0) |  |  |  |  |  |  |  |  |  |  |  |  |  |  |  |
| **1998** |  | 336 (0.9) | (0.0) | 96 (0.0) | 8202 (0.5) | (0.0) |  | 181 (0.0) |  | 400 (0.5) |  |  | 28 (0.0) | 25 (0.0) |  |  | 1299 (0.0) |  | 13 (0.0) |
|  |  | 128 (2.3) |  |  | 2827 (12.6) |  |  |  |  |  |  |  |  |  |  |  |  |  |  |
| **1999** |  | 78 (0.0) | (0.0) | 88 (0.0) | 8000 (0.1) | (0.0) |  | 74 (0.0) |  |  |  | 135 (5.2) | 1 (0.0) | 37 (0.0) |  |  | 595 (0.0) |  | 3 (0.0) |
|  |  | 334 (0.0) |  | 176 (0.0) |  |  |  | (0.0) |  |  |  | (5.0) |  |  |  |  | 301 (0.0) |  |  |
| **2000** |  | 291 (0.3) | (0.0) | 369 (0.0) | 7200 (0.2) |  |  | (0.0) |  |  |  | 58 (5.5) |  | 207 (0.0) |  |  | 525 (0.0) |  | 2 (0.0) |
| **2001** |  | 158 (1.3) | (0.0) | 580 (0.0) | 3714 (1.6) |  |  | 166 (0.6) |  | (59.4) |  | 73 (5.5) |  | 1516 (0.0) |  |  | 375 (0.0) |  | 2 (0.0) |
|  |  |  |  |  |  |  |  |  |  |  |  |  |  | (1.0) |  |  |  |  |  |
| **2002** |  | 124 (7.3) | (0.0) | 488 (0.0) | 76 (19.7) |  | 54 (0.0) | 126 (0.0) |  |  |  | 34 (8.8) |  | 423 (0.0) |  |  | 120 (0.8) |  |  |
|  |  |  |  |  | (29.9) |  |  |  |  |  |  | (5.0) |  |  |  |  |  |  |  |
|  |  |  |  |  |  |  |  |  |  |  |  | (11.8) |  |  |  |  |  |  |  |
|  |  |  |  |  |  |  |  |  |  |  |  | (18.6) |  |  |  |  |  |  |  |
| **2003** |  | 203 (1.5) |  | 512 (0.0) | 1688 (14.2) |  | 160 (0.6) | 31 (0.0) |  |  |  | 93 (1.1) |  | 641 (3.3) | (0.0) |  | 237 (0.0) |  |  |
| **2004** |  | 309 (1.3) |  | 342 (0.0) | 1705 (7.4) |  | 93 (0.0) | 191 (1.6) |  | (22.0) | 19 (5.3) | 155 (1.9) |  | 87 (0.0) |  |  | 258 (0.0) |  |  |
|  |  |  |  | 353 (0.0) | (18.0) |  |  |  |  |  |  |  |  | 395 (26.3) |  |  |  |  |  |
|  |  |  |  |  |  |  |  |  |  |  |  |  |  | 3154 (8.3) |  |  |  |  |  |
| **2005** | 338 (3.6) | 265 (1.1) |  | 293 (0.0) | 761 (13.7) |  | 217 (0.0) |  |  |  | 133 (0.0) | 194 (1.0) |  | (12.1) |  |  | 456 (0.0) |  |  |
|  | (1.7) |  |  |  |  |  |  |  |  |  | 111 (0.0) |  |  | (10.8) |  |  |  |  |  |
|  | (3.4) |  |  |  |  |  |  |  |  |  | 22 (0.0) |  |  | (9.6) |  |  |  |  |  |
|  | (5.1) |  |  |  |  |  |  |  |  |  |  |  |  |  |  |  |  |  |  |
| **2006** |  | 238 (0.4) |  | (0.6) | 310 (11.6) |  | 326 (0.0) |  |  |  | (6.5) | 191 (0.5) |  |  |  |  | 444 (0.0) | 187 (0.0) |  |
|  |  |  |  | (2.6) | 426 (25.0) |  |  |  |  |  | 147 (0.0) |  |  |  |  |  |  |  |  |
|  |  |  |  | (4.5) |  |  |  |  |  |  | 146 (0.0) |  |  |  |  |  |  |  |  |
|  |  |  |  | 281 (0.0) |  |  |  |  |  |  | 1 (0.0) |  |  |  |  |  |  |  |  |
| **2007** |  | 197 (1.5) |  |  |  |  |  |  |  |  | 30 (0.0) | 224 (0.9) |  |  | 750 (0.8) |  | 388 (0.0) |  | 1 (0.0) |
|  |  |  |  |  |  |  |  |  |  |  | 22 (0.0) |  |  |  |  |  |  |  |  |
|  |  |  |  |  |  |  |  |  |  |  | 8 (0.0) |  |  |  |  |  |  |  |  |
| **2008** | 127 (11.0) |  |  |  |  |  |  |  |  |  | 61 (1.6) |  |  |  |  |  |  |  |  |
|  |  |  |  |  |  |  |  |  |  |  | 77 (1.3) |  |  |  |  |  |  |  |  |
| **2009** |  |  |  |  |  |  |  | 255 (0.0) | 109 (0.9) |  | 66 (0.0) |  |  |  | (0.3) |  |  |  |  |
|  |  |  |  |  |  |  |  |  |  |  | 16 (6.3) |  |  |  |  |  |  |  |  |
| **2010** |  | 181 (3.9) |  |  |  |  |  | 454 (0.2) |  |  |  |  |  |  | 2925 (0.4) |  |  |  |  |
|  |  |  |  |  |  |  |  |  |  |  |  |  |  |  | (0.8) |  |  |  |  |
|  |  |  |  |  |  |  |  |  |  |  |  |  |  |  | (1.6) |  |  |  |  |
| **2011** | 4681 (0.9) |  |  |  |  |  | 304 (0.0) | 373 (0.0) |  |  | 173 (2.3) | 929 (1.4) | 65 (0.0) |  | 3441 (0.6) |  | 478 (0.0) |  |  |
|  |  |  |  |  |  |  |  |  | (7.8) |  |  |  |  |  |  |  |  |  |  |

Afg: Afghanistan, Bah: Bahrain, Dji: Djibouti, Egy: Egypt, Irn: Iran, Irq: Iraq, Jor: Jordan, Kuw: Kuwait, Leb: Lebanon, Lib: Libya, Mor: Morocco, Oma: Oman, OPT: Occupied Palestinian Territories, Pak: Pakistan, SA: Saudi Arabia, Sud: Sudan, Syr: Sria, Tun: Tunisia, Yem: Yemen
