# Supplementary material for: HIV among People Who Inject Drugs in the Middle East and North Africa: Systematic Review and Data Synthesis
Source: PLoS Med. 2014 Jun 17;11(6):e1001663. doi: 10.1371/journal.pmed.1001663 (PMC4061009; doi:10.1371/journal.pmed.1001663)
Supplement: Table S5 — Measures of injecting risk behavior among people who inject drugs in the Middle East and North Africa. (DOCX) [file pmed.1001663.s005.docx]

**Table S5. Measures of injecting risk behavior among people who inject drugs in the Middle East and North Africa**

|  | **Afghanistan** | **Algeria** | **Bahrain** | **Egypt** | **Iran** | **Jordan** | **Lebanon** | **Libya** | **Morocco** | **Oman** | **OPT** | **Pakistan** | **Syria** | **Tunisia** |
| --- | --- | --- | --- | --- | --- | --- | --- | --- | --- | --- | --- | --- | --- | --- |
| **Shared needles or syringes (%)** |  |  |  |  |  |  |  |  |  |  |  |  |  |  |
| Ever | 8 [[1](#_ENREF_1)], 11 [[2](#_ENREF_2)], 17 [[3](#_ENREF_3)], 27 [[2](#_ENREF_2)], 34 [[2](#_ENREF_2)], 50 [[4](#_ENREF_4)] |  |  | 59 [[5](#_ENREF_5)] | 12 [[6](#_ENREF_6)], 12 [[7](#_ENREF_7)] 14 [[8](#_ENREF_8)], 18 [[9](#_ENREF_9)] 20 [[10](#_ENREF_10)], 25 [[11](#_ENREF_11)] 28 [[12](#_ENREF_12)], 31 [[13](#_ENREF_13)] 31 [[14](#_ENREF_14)], 32 [[15](#_ENREF_15)] 32 [[16](#_ENREF_16)], 34 [[17](#_ENREF_17)], 35 [[18](#_ENREF_18)], 36 [[19](#_ENREF_19)], 36 [[20](#_ENREF_20)], 37 [[21](#_ENREF_21)], 48 [[22](#_ENREF_22)] 49 [[9](#_ENREF_9)], 49 [[18](#_ENREF_18)] 50 [[23](#_ENREF_23)], 66 [[23](#_ENREF_23)] 76 [[24](#_ENREF_24)], 70 [[25](#_ENREF_25)], 71 [[25](#_ENREF_25)], 95 [[26](#_ENREF_26),[27](#_ENREF_27)] | 63 [[28](#_ENREF_28)], 64 [[28](#_ENREF_28)], 71 [[29](#_ENREF_29)] | 41 [[30](#_ENREF_30)], 65 [[31](#_ENREF_31)] | 85 [[32](#_ENREF_32)] | 47 [[33](#_ENREF_33)] | 90 [[34](#_ENREF_34)], 94 [[34](#_ENREF_34)], 97 [[34](#_ENREF_34)] |  | 14 [[35](#_ENREF_35)], 17 [[36](#_ENREF_36)], 41 [[35](#_ENREF_35)], 46 [[37](#_ENREF_37)], 50 [[38](#_ENREF_38)], 53 [[39](#_ENREF_39)], 56 [[38](#_ENREF_38)], 56 [[38](#_ENREF_38)], 58 [[40](#_ENREF_40)], 63 [[38](#_ENREF_38)], 63 [[38](#_ENREF_38)], 64 [[41](#_ENREF_41)], 67 [[36](#_ENREF_36)], 74 [[38](#_ENREF_38)], 68 [[41](#_ENREF_41)], 69 [[39](#_ENREF_39)], 70 [[39](#_ENREF_39)], 79 [[39](#_ENREF_39)], | 46 [[42](#_ENREF_42)] |  |
| Last 6 months | 16 [[43](#_ENREF_43)], 28 [[44](#_ENREF_44)], 29 [[44](#_ENREF_44)], 34 [[43](#_ENREF_43)], 45 [[43](#_ENREF_43)] |  |  |  | 47 [[45](#_ENREF_45)], 64 |  |  |  |  |  |  | 47 [[46](#_ENREF_46)] |  |  |
| Last 3 months | 6 [[3](#_ENREF_3)] |  |  |  |  |  |  |  |  |  |  |  |  |  |
| Last month |  |  |  | 23 [[47](#_ENREF_47)], 32 [[48](#_ENREF_48)], 41 [[47](#_ENREF_47)], 53 [[48](#_ENREF_48)], 55 [[5](#_ENREF_5)], 86 [[49](#_ENREF_49)] | 8 [[13](#_ENREF_13)], 12 [[50](#_ENREF_50)], 13 [[51](#_ENREF_51)], 14 [[50](#_ENREF_50)], 14 [[52](#_ENREF_52)], 18 [[50](#_ENREF_50)], 21 [[25](#_ENREF_25)], 40 [[25](#_ENREF_25)], 73 [[53](#_ENREF_53)], 100 [[54](#_ENREF_54)] |  |  |  | 30 [[55](#_ENREF_55)], 33 [[55](#_ENREF_55)], 36 [[55](#_ENREF_55)], 36 [[55](#_ENREF_55)] |  |  | 54 [[56](#_ENREF_56)] | 28 [[42](#_ENREF_42)] |  |
| Last week |  |  |  |  | 9 [[57](#_ENREF_57)], 23 [[57](#_ENREF_57)] |  |  |  |  |  |  |  |  |  |
| Last injection |  |  |  | 25 [[49](#_ENREF_49)] | 6 [[26](#_ENREF_26)], 11 [[22](#_ENREF_22)] | 61 [[29](#_ENREF_29)] | 17 [[31](#_ENREF_31)], 21 [[58](#_ENREF_58)] | 18 [[32](#_ENREF_32)] |  |  | 11 [[59](#_ENREF_59)], 19 [[60](#_ENREF_60)] | 18 [[61](#_ENREF_61)], 18 [[62](#_ENREF_62)], 18 [[62](#_ENREF_62)], 22 [[63](#_ENREF_63)], 23 [[61](#_ENREF_61)], 23 [[64](#_ENREF_64)], 23 [[65](#_ENREF_65)], 24 [[62](#_ENREF_62)], 24 [[62](#_ENREF_62)], 25 [[65](#_ENREF_65)], 28 [[63](#_ENREF_63)], 30 [[65](#_ENREF_65)], 31 [[64](#_ENREF_64)], 35 [[66](#_ENREF_66)], 47 [[66](#_ENREF_66)], 82 [[65](#_ENREF_65)] |  |  |
| Currently |  |  |  |  |  |  |  |  |  | 44 [[34](#_ENREF_34)], 65 [[34](#_ENREF_34)], 70 [[34](#_ENREF_34)] |  | 48 [[67](#_ENREF_67)], 57 [[68](#_ENREF_68)], 66 [[69](#_ENREF_69)], 72 [[67](#_ENREF_67)] |  |  |
| Unspecified |  | 41 [[70](#_ENREF_70)] | 79 [[71](#_ENREF_71)] | 11 [[72](#_ENREF_72)], 15 [[73](#_ENREF_73)], 40 [[74](#_ENREF_74)], 43 [[75](#_ENREF_75)], | 0 [[76](#_ENREF_76)], 29 [[77](#_ENREF_77)], 31 [[78](#_ENREF_78)], 33 [[79](#_ENREF_79)], 36 [[80](#_ENREF_80)], 48 [[81](#_ENREF_81)], 50 [[82](#_ENREF_82)], 59 [[83](#_ENREF_83)], 70 [[84](#_ENREF_84)] |  |  |  |  |  | 39 [[85](#_ENREF_85)], 47 [[85](#_ENREF_85)] | 8 [[86](#_ENREF_86)], 46 [[87](#_ENREF_87)], 52 [[88](#_ENREF_88)], 80 [[38](#_ENREF_38)], 86 [[89](#_ENREF_89)], 88 [[38](#_ENREF_38)], 90 [[90](#_ENREF_90)], 95 [[91](#_ENREF_91)] |  |  |
| **Clean used needle before use (%)** |  |  |  |  |  |  |  |  |  |  |  |  |  |  |
| Always | 14 [[5](#_ENREF_5)] |  |  |  | 14 [[45](#_ENREF_45)] | 34 [[29](#_ENREF_29)] |  | 86 [[32](#_ENREF_32)] | 70 [[55](#_ENREF_55)], 85 [[55](#_ENREF_55)] |  |  | 43 [[92](#_ENREF_92)], 64 [[36](#_ENREF_36)] | 60%[[42](#_ENREF_42)] |  |
| In the last month | 34 [[47](#_ENREF_47)], 83 [[47](#_ENREF_47)] |  |  |  |  |  |  |  |  |  |  |  |  |  |
| Last injection |  |  |  |  |  |  |  |  |  |  | 54 [[59](#_ENREF_59)] | 49 [[93](#_ENREF_93)] |  |  |
| Unspecified |  |  |  |  |  | 83 [[28](#_ENREF_28)] |  |  |  |  | 36 [[85](#_ENREF_85)] |  |  |  |
| **Method of cleaning (%)** |  |  |  |  |  |  |  |  |  |  |  |  |  |  |
| Water |  |  |  | 72 [[5](#_ENREF_5)] |  | 79 [[28](#_ENREF_28)] |  | 82 [[32](#_ENREF_32)] | 89 [[55](#_ENREF_55)], 97 [[55](#_ENREF_55)] |  | 68 [[59](#_ENREF_59)] 84 [[85](#_ENREF_85)] | 34 [[36](#_ENREF_36)], 39 [[65](#_ENREF_65)], 54 [[93](#_ENREF_93)], 78 [[46](#_ENREF_46)], 84 [[92](#_ENREF_92)], 100 [[65](#_ENREF_65)] | 40 [[42](#_ENREF_42)] | 82 [[94](#_ENREF_94)] |
| Bleach |  |  |  |  |  |  |  | 9 [[32](#_ENREF_32)] | 4 [[55](#_ENREF_55)], 7 [[55](#_ENREF_55)] |  |  | 0 [[46](#_ENREF_46)], 5 [[93](#_ENREF_93)] | 3 [[42](#_ENREF_42)] | 8 [[94](#_ENREF_94)] |
| **Last time injected with (%)** |  |  |  |  |  |  |  |  |  |  |  |  |  |  |
| Friends/acquaintances |  |  |  |  |  |  |  |  |  |  |  | 56 [[65](#_ENREF_65)], 64 [[63](#_ENREF_63)], 64 [[61](#_ENREF_61)], 67 [[66](#_ENREF_66)], 72 [[65](#_ENREF_65)], 81 [[64](#_ENREF_64)] |  |  |
| Strangers |  |  |  |  |  |  |  |  |  |  |  | 0 [[64](#_ENREF_64)], 0 [[66](#_ENREF_66)], 0 [[61](#_ENREF_61)], 1 [[63](#_ENREF_63)], 2 [[65](#_ENREF_65)], 3 [[65](#_ENREF_65)] |  |  |
| Alone |  |  |  |  |  |  |  |  |  |  | 45 [[85](#_ENREF_85)], 61 [[59](#_ENREF_59)] | 15 [[65](#_ENREF_65)], 18 [[36](#_ENREF_36)], 18 [[64](#_ENREF_64)], 27 [[66](#_ENREF_66)], 32 [[63](#_ENREF_63)], 34 [[61](#_ENREF_61)], 36 [[65](#_ENREF_65)], 42 [[93](#_ENREF_93)] |  |  |
| **Place of last injection (%)** |  |  |  |  |  |  |  |  |  |  |  |  |  |  |
| Public place |  |  |  |  |  |  |  |  | 21 [[33](#_ENREF_33)], 46 [[55](#_ENREF_55)], 51 [[55](#_ENREF_55)] |  | 47 [[85](#_ENREF_85)] | 25 [[40](#_ENREF_40)], 66 [[63](#_ENREF_63)], 78 [[66](#_ENREF_66)], 79 [[65](#_ENREF_65)], 82 [[61](#_ENREF_61)], 89 [[65](#_ENREF_65)], 91 [[64](#_ENREF_64)] |  |  |
| Home |  |  |  |  |  |  |  |  | 33 [[55](#_ENREF_55)], 41 [[33](#_ENREF_33)], 46 [[55](#_ENREF_55)] |  | 54 [[85](#_ENREF_85)] | 3 [[65](#_ENREF_65)], 5 [[64](#_ENREF_64)], 9 [[61](#_ENREF_61)], 11 [[65](#_ENREF_65)], 11 [[66](#_ENREF_66)], 14 [[63](#_ENREF_63)], 68 [[40](#_ENREF_40)] | 74 [[42](#_ENREF_42)] |  |
| **Used “street doctors” (%)** |  |  |  |  |  |  |  |  |  |  |  |  |  |  |
| Ever |  |  |  |  |  |  |  |  |  |  |  | 26 [[63](#_ENREF_63)], 42 [[38](#_ENREF_38)], 77 [[65](#_ENREF_65)], 84 [[38](#_ENREF_38)], 99 [[38](#_ENREF_38)], 100 [[38](#_ENREF_38)] |  |  |
| Last injection |  |  |  |  |  |  |  |  |  |  |  | 247 [[66](#_ENREF_66)], 32 [[61](#_ENREF_61)], 44 [[64](#_ENREF_64)] |  |  |
| Unspecified |  |  |  |  |  |  |  |  |  |  |  | 11 [[67](#_ENREF_67)], 11 [[67](#_ENREF_67)], 19 [[93](#_ENREF_93)], 46 [[39](#_ENREF_39)], 58 [[39](#_ENREF_39)], 59 [[69](#_ENREF_69)], 61 [[39](#_ENREF_39)], 71 [[39](#_ENREF_39)], 73 [[36](#_ENREF_36)], 85 [[36](#_ENREF_36)] |  |  |
| **Average # of injections** |  |  |  |  |  |  |  |  |  |  |  |  |  |  |
| /day | 5.7 [[3](#_ENREF_3)] |  |  |  | 3.3 [[95](#_ENREF_95)] |  |  |  |  |  |  | 1.1 [[36](#_ENREF_36)], 2 [[69](#_ENREF_69)], 2 [[56](#_ENREF_56)], 2.2 [[65](#_ENREF_65)], 2.2 [[63](#_ENREF_63)], 2.2 [[61](#_ENREF_61)], 2.3 [[96](#_ENREF_96)], 2.3 [[65](#_ENREF_65)], 2.3 [[66](#_ENREF_66)] |  |  |
| /week |  |  |  |  | 7.7 [[97](#_ENREF_97)], 21.1 [[98](#_ENREF_98)], 21.3 [[97](#_ENREF_97)], |  |  |  |  |  |  |  |  |  |
| /last month |  |  |  |  | 54.0 [[19](#_ENREF_19)], 92.6 [[25](#_ENREF_25)], 104.3 [[99](#_ENREF_99)], 110.3 [[25](#_ENREF_25)] |  |  |  |  |  |  | 20 [[100](#_ENREF_100)], 63 [[101](#_ENREF_101)], 67 [[101](#_ENREF_101)] | 41 [[42](#_ENREF_42)] |  |
| **Age at first injection** |  |  |  |  |  |  |  |  |  |  |  |  |  |  |
| Mean (years) | 25.8 [[3](#_ENREF_3)], 26.4 [[4](#_ENREF_4)] |  |  | 23.1 [[47](#_ENREF_47)], 23.3 [[5](#_ENREF_5)], 27.0 [[47](#_ENREF_47)] | 23.6 [[13](#_ENREF_13)], 23.7 [[102](#_ENREF_102)], 23.9 [[103](#_ENREF_103)], 24.0 [[79](#_ENREF_79)], 24.0 [[15](#_ENREF_15)], 25.0 [[18](#_ENREF_18)], 25.9 [[51](#_ENREF_51)], 26.0 [[50](#_ENREF_50)], 26.3 [[50](#_ENREF_50)], 26.5 [[50](#_ENREF_50)], 27.0 [[104](#_ENREF_104)], 27.3 [[76](#_ENREF_76)], 27.4 [[104](#_ENREF_104)], 28.1 [[18](#_ENREF_18)] |  |  |  | 32.9 [[33](#_ENREF_33)] | 20 [[34](#_ENREF_34)], 22 [[34](#_ENREF_34)], 22 [[34](#_ENREF_34)] | 28.8 [[59](#_ENREF_59)] | 25.0 [[39](#_ENREF_39)], 25.2 [[38](#_ENREF_38)], 25.6 [[64](#_ENREF_64)], 27.0 [[105](#_ENREF_105)], 27.0 [[63](#_ENREF_63)], 27.2 [[36](#_ENREF_36)], 27.9 [[66](#_ENREF_66)], 28.1 [[38](#_ENREF_38)], 28.3 [[38](#_ENREF_38)], 28.5 [[61](#_ENREF_61)], 29.1 [[38](#_ENREF_38)], 31.6 [[36](#_ENREF_36)] | 27.0 [[42](#_ENREF_42)] |  |
| Median (years) | 26.0 [[43](#_ENREF_43)] |  |  |  | 25.0 [[22](#_ENREF_22)], 25.0 [[14](#_ENREF_14)] |  |  |  | 21.0 [[55](#_ENREF_55)], 28.0 [[55](#_ENREF_55)] |  |  | 25.0 [[46](#_ENREF_46)] |  |  |
| **Duration of injecting** |  |  |  |  |  |  |  |  |  |  |  |  |  |  |
| Mean (years) | 1.3 [[106](#_ENREF_106)], 1.4 [[106](#_ENREF_106)], 1.7 [[2](#_ENREF_2)], 2.1 [[2](#_ENREF_2)], 2.3 [[106](#_ENREF_106)], 2.4 [[106](#_ENREF_106)], 2.5 [[2](#_ENREF_2)], 2.9 [[106](#_ENREF_106)], 3.3 [[43](#_ENREF_43)], 4.0 [[3](#_ENREF_3)], 4.4 [[4](#_ENREF_4)] |  |  | 6.4 [[47](#_ENREF_47)], 9.3 [[47](#_ENREF_47)] | 1.6 [[76](#_ENREF_76)], 2.0 [[18](#_ENREF_18)], 2.6 [[18](#_ENREF_18)], 3.8 [[23](#_ENREF_23)], 4.1 [[107](#_ENREF_107)], 4.5 [[79](#_ENREF_79)], 4.5 [[15](#_ENREF_15)], 4.8 [[23](#_ENREF_23)], 4.8 [[25](#_ENREF_25)], 4.9 [[25](#_ENREF_25)], 5.4 [[13](#_ENREF_13)], 5.8 [[95](#_ENREF_95)], 6.7 [[98](#_ENREF_98)], 7.4 [[26](#_ENREF_26)], 8.4 [[27](#_ENREF_27)], 12 [[99](#_ENREF_99)] |  |  |  |  |  |  | 2.1 [[105](#_ENREF_105)], 4.2 [[65](#_ENREF_65)], 4.4 [[36](#_ENREF_36)], 4.6 [[65](#_ENREF_65)], 4.6 [[61](#_ENREF_61)], 4.9 [[64](#_ENREF_64)], 5.3 [[63](#_ENREF_63)], 5.9 [[66](#_ENREF_66)], 6.1 [[35](#_ENREF_35)], 7.6 [[35](#_ENREF_35)], 7.7 [[36](#_ENREF_36)] |  |  |
| Median (years) |  |  |  |  | 6 [[22](#_ENREF_22)] |  |  |  | 8.0 [[55](#_ENREF_55)], 8.7 [[55](#_ENREF_55)] |  |  | 3 [[41](#_ENREF_41)], 7 [[41](#_ENREF_41)] |  |  |
| **Re-injecting own blood (%)** |  |  |  |  |  |  |  |  |  |  |  |  |  |  |
| Ever | 69 [[43](#_ENREF_43)], 70 [[3](#_ENREF_3)], 73 [[43](#_ENREF_43)], 81 [[43](#_ENREF_43)], 83 [[44](#_ENREF_44)] |  |  |  |  |  |  |  |  |  |  | 70 [[38](#_ENREF_38)], 91 [[41](#_ENREF_41)], 92 [[41](#_ENREF_41)], 92 [[38](#_ENREF_38)], 94 [[38](#_ENREF_38)], 96 [[38](#_ENREF_38)] |  |  |
| **Selling or donating blood (%)** |  |  |  |  |  |  |  |  |  |  |  |  |  |  |
| Ever | 5 [[4](#_ENREF_4)] |  |  | 30 [[5](#_ENREF_5)] | 56 [[108](#_ENREF_108)] |  |  |  |  |  |  | 3 [[35](#_ENREF_35)], 8 [[46](#_ENREF_46)], 12 [[105](#_ENREF_105)], 23 [[69](#_ENREF_69)], 27 [[35](#_ENREF_35)], 28 [[96](#_ENREF_96)], 31 [[36](#_ENREF_36)], 44 [[68](#_ENREF_68)] | 23 [[42](#_ENREF_42)] |  |
| Last 12 months |  |  |  |  |  |  |  |  |  |  |  | 1 [[62](#_ENREF_62)], 2 [[62](#_ENREF_62)] |  |  |
| Last 6 months |  |  |  |  |  |  |  |  |  |  |  | 1 [[38](#_ENREF_38)], 5 [[61](#_ENREF_61)], 5 [[38](#_ENREF_38)], 9 [[38](#_ENREF_38)], 11 [[38](#_ENREF_38)] |  |  |

OPT: Occupied Palestinian Territories

**References**

1. Action Aid Afghanistan (2006) A Study on Knowledge, Attitude, Behaviour and Practice in High Risk and Vulnerable Groups in Afghanistan.

2. Afghanistan National AIDS Control Program (2010) Integrated Behavioral & Biological Surveillance (IBBS) in Afghanistan: Year 1 Report. HIV Surveillance Project - Johns Hopkins University School of Public Health, National AIDS Control Program, Ministry of Public Health. Kabul, Afghanistan.

3. Todd CS, Nasir A, Stanekzai MR, Fiekert K, Rasuli MZ, et al. (2011) Prevalence and correlates of HIV, syphilis, and hepatitis B and C infection and harm reduction program use among male injecting drug users in Kabul, Afghanistan: A cross-sectional assessment. Harm Reduct J 8: 22.

4. Todd CS, Abed AM, Strathdee SA, Scott PT, Botros BA, et al. (2007) HIV, hepatitis C, and hepatitis B infections and associated risk behavior in injection drug users, Kabul, Afghanistan. Emerg Infect Dis 13: 1327-1331.

5. Elshimi T, Warner-Smith M, Aon M (2004) Blood-borne virus risks of problematic drug users in Greater Cairo. Geneva, UNAIDS & UNODC. August.

6. Kheirandish P, Seyedalinaghi SA, Hosseini M, Jahani MR, Shirzad H, et al. (2010) Prevalence and correlates of HIV infection among male injection drug users in detention in Tehran, Iran. J Acquir Immune Defic Syndr 53: 273-275.

7. Khani M, Vakili MM (2003) Prevalence and risk factors of HIV, hepatitis B virus and hepatitis C virus infections in drug addicts among Zanjan prisoners. Archives of Iranian Medicine 6: 1-4.

8. Amiri M, Khosravi A, Chaman R (2010) Drug abuse pattern and high risk behaviors among addicts in Shahroud County of Semnan Province, Northeast Iran in 2009. Journal of Research in Health Sciences 10: 104-109.

9. Rahbar AR, Rooholamini S, Khoshnood K (2004) Prevalence of HIV infection and other blood-borne infections in incarcerated and non-incarcerated injection drug users (IDUs) in Mashhad, Iran. International Journal of Drug Policy 15: 151-155.

10. Imani R, Karimi A, Rouzbahani R, Rouzbahani A (2008) Seroprevalence of HBV, HCV and HIV infection among intravenous drug users in Shahr-e-Kord, Islamic Republic of Iran. East Mediterr Health J 14: 1136-1141.

11. Sayad B, Saeed FS, Keyvani H, Rezali M, Asadi T, et al. (2008) Seroepidemiology of hepatitis C in Kermanshah (West of Iran, 2006). Hepatitis Monthly 8: 141-146.

12. Malekinejad M, Mohraz M, Razani N, Khairandish P, McFarland W, et al. HIV and related risk behaviors of injecting drug users (IDU) in Iran: findings from the first respondent-driven sampling (RDS) survey of IDU in Tehran in 2006-2007. Abstract no. THAC0202 2008; Mexico.

13. Zamani S, Radfar R, Nematollahi P, Fadaie R, Meshkati M, et al. (2010) Prevalence of HIV/HCV/HBV infections and drug-related risk behaviours amongst IDUs recruited through peer-driven sampling in Iran. Int J Drug Policy 21: 493-500.

14. Zamani S, Kihara M, Gouya MM, Vazirian M, Ono-Kihara M, et al. (2005) Prevalence of and factors associated with HIV-1 infection among drug users visiting treatment centers in Tehran, Iran. AIDS 19: 709-716.

15. Momtazi S, Fallahnejad M, Shoghli A, Musavinasab N, Tavassoli S (2010) HIV high risk behavior in a sample of Iranian injection drug users. Abstract no. TUPE0338. AIDS 2010 - XVIII International AIDS Conference. Vienna, Austria.

16. Vahdani P, Hosseini-Moghaddam SM, Family A, Moheb-Dezfouli R (2009) Prevalence of HBV, HCV, HIV and syphilis among homeless subjects older than fifteen years in Tehran. Arch Iran Med 12: 483-487.

17. Javadi A, Ataei B, Yaran M, Nokhodian Z, Kassaian N, et al. (2013) Prevalence of HIV infection and related risk factors in Isfahan Drop in Centers. Pakistan Journal of Medical Sciences 29: 346-350.

18. Farhoudi B, Montevalian A, Motamedi M, Khameneh MM, Mohraz M, et al. (2003) Human immunodeficiency virus and HIV - associated tuberculosis infection and their risk factors in injecting drug users in prison in Iran. Iran Ministry of Health, Tehran, Iran.

19. Dibaj R, Ataei B, Yaran M, Nokhodian Z, Tayeri K, et al. (2013) Prevalence of HIV infection in inmates with history of injection drug use and evaluation of risk factors, in Isfahan, Iran. Pakistan Journal of Medical Sciences 29: 399-402.

20. Rafiey H, Narenjiha H, Shirinbayan P, Noori R, Javadipour M, et al. (2009) Needle and syringe sharing among Iranian drug injectors. Harm Reduct J 6: 21.

21. Hassannejad R, Kassaian N, Ataei B, Adibi P (2012) High risky behaviors among intravenous drug users in Isfahan, Iran: A study for hepatitis c harm reduction programs. International Journal of Preventive Medicine 3.

22. Zamani S, Kihara M, Gouya MM, Vazirian M, Nassirimanesh B, et al. (2006) High prevalence of HIV infection associated with incarceration among community-based injecting drug users in Tehran, Iran. J Acquir Immune Defic Syndr 42: 342-346.

23. Mir-Nasseri MM, Mohammadkhani A, Tavakkoli H, Ansari E, Poustchi H (2011) Incarceration is a major risk factor for blood-borne infection among intravenous drug users. Hepatitis Monthly 11: 19-22.

24. Day C, Nassirimanesh B, Shakeshaft A, Dolan K (2006) Patterns of drug use among a sample of drug users and injecting drug users attending a General Practice in Iran. Harm Reduct J 3: 2.

25. Zamani S, Vazirian M, Nassirimanesh B, Razzaghi EM, Ono-Kihara M, et al. (2010) Needle and syringe sharing practices among injecting drug users in Tehran: a comparison of two neighborhoods, one with and one without a needle and syringe program. AIDS Behav 14: 885-890.

26. Kazerooni PA, Lari MA, Joolaei H, Parsa N (2010) Knowledge and attitude of male intravenous drug users on HIV/AIDS associated high risk behaviors in Shiraz Pir-Banon jail, Fars Province, Southern Iran. Iranian Red Crescent Medical Journal 12: 334-336.

27. Amin-Esmaeili M, Rahimi-Movaghar A, Razaghi EM, Baghestani AR, Jafari S (2012) Factors correlated with hepatitis C and B virus infections among injecting drug users in Tehran, IR Iran. Hepatitis Monthly 12: 23-31.

28. Shahroury M (2011) Assessment report on injecting drug users in Jordan. Future Guardians Forum Association, Amman, Jordan.

29. Jordan National AIDS Program (2010) Preliminary analysis of Jordan IBBSS among injecting drug users. Ministry of Health, Amman, Jordan.

30. Aaraj E Report on the situation analysis on vulnerable groups in Beirut, Lebanon. Lebanon Ministry of Health, Beirut, Lebanon.

31. Hermez J HIV/AIDS prevention through outreach to vulnerable populations in Beirut, Lebanon. Final Report.

32. Mirzoyan L, Berendes S, Jeffery C, Thomson J, Ben Othman H, et al. (2013) New evidence on the HIV epidemic in Libya: why countries must implement prevention programs among people who inject drugs. J Acquir Immune Defic Syndr 62: 577-583.

33. Ministère de la Santé au Maroc, Direction de l’Épidémiologie et de Lutte contre les Maladies, Programme de lutte contre la toxicomanie (2006) Evaluation rapide de la situation sur le risque d’infection à VIH en relation avec l’usage des drogues injectées et injectables et à problème au Maroc (French) [Rapid situation assessment on the risk of HIV infection associated with the use of injected, injectable, and other drugs in Morocco]. Rabat, Morocco.

34. Oman Ministry of Health (2006) HIV Risk among Heroin and Injecting Drug Users in Muscat, Oman. Quantitative Survey. Preliminary Data. Muscat, Oman.

35. Platt L, Vickerman P, Collumbien M, Hasan S, Lalji N, et al. (2009) Prevalence of HIV, HCV and sexually transmitted infections among injecting drug users in Rawalpindi and Abbottabad, Pakistan: evidence for an emerging injection-related HIV epidemic. Sex Transm Infect 85 Suppl 2: ii17-22.

36. Altaf A, Shah SA, A. M (2003) Follow up study to assess and evaluate knowledge, attitude and high risk behaviors and prevalence of HIV, HBV, HCV and Syphilis among IDUS at Burns Road DIC, Karachi. External report submitted to UNODC.

37. Kazi AM, Shah SA, Jenkins CA, Shepherd BE, Vermund SH (2010) Risk factors and prevalence of tuberculosis, human immunodeficiency virus, syphilis, hepatitis B virus, and hepatitis C virus among prisoners in Pakistan. Int J Infect Dis 14 Suppl 3: e60-66.

38. Nai Zindagi, Punjab Provincial AIDS Control Program (2005) The lethal overdose: Injecting drug use and HIV/AIDS.

39. Nai Zindagi, Punjab Provincial AIDS Control Program (2009) Rapid situation assessments of HIV prevalence and risk factors among people injecting drugs in four cities of the Punjab.

40. United Nations Office on Drugs and Crime (UNODC), Country Office Pakistan (2010) Female drug use in Pakistan: Mapping estimates, ethnographic results & behavioural assessment.

41. Kuo I, ul-Hasan S, Galai N, Thomas DL, Zafar T, et al. (2006) High HCV seroprevalence and HIV drug use risk behaviors among injection drug users in Pakistan. Harm Reduct J 3: 26.

42. Syria Mental Health Directorate, Syria National AIDS Programme (2008) Assessment of HIV Risk and Sero-prevalence among Drug Users in Greater Damascus. Syrian Ministry of Health. UNODC. UNAIDS. Damascus, Syria

43. Nasir A, Todd CS, Stanekzai MR, Bautista CT, Botros BA, et al. (2011) Prevalence of HIV, hepatitis B and hepatitis C and associated risk behaviours amongst injecting drug users in three Afghan cities. Int J Drug Policy 22: 145-152.

44. Todd CS, Abed AM, Scott PT, Botros BA, Safi N, et al. (2008) Correlates of receptive and distributive needle sharing among injection drug users in Kabul, Afghanistan. Am J Drug Alcohol Abuse 34: 91-100.

45. Radfar R, Pooya A, Meshkaty M, Fadai R, Soltanolkotaby MA (2007) Pattern of behaviors and risks among injecting drug users coming in HAFTOON drop in center, Isfahan, Iran. 18th International Conference on the Reduction of Drug Related Harm, Warsaw, Poland.

46. Parviz S, Fatmi Z, Altaf A, McCormick JB, Fischer-Hoch S, et al. (2006) Background demographics and risk behaviors of injecting drug users in Karachi, Pakistan. Int J Infect Dis 10: 364-371.

47. Family Health International and Ministry of Health Egypt (2010) HIV/AIDS Biological & Behavioral Surveillance Survey: Round Two Summary Report, Cairo, Egypt 2010. FHI in collaboration with the Ministry of Health and support from the Global Fund. Found at <http://www.fhi360.org/sites/default/files/media/documents/BBSS%202010_0.pdf>, Last accessed February 2014.

48. Soliman C, Rahman IA, Shawky S, Bahaa T, Elkamhawi S, et al. (2010) HIV prevalence and risk behaviors of male injection drug users in Cairo, Egypt. AIDS 24 Suppl 2: S33-38.

49. E. Elghamrawy, O. Abaza, S. Abou Elmagd, H. Ramy, S. Atallah, et al. Risk behaviours among male injecting drug users in Egypt. Abstract no. MOPE227 2012; Washington DC, USA.

50. Iran Ministry of Health and Medical Education , Kyoto University School of Public Hleath (Japan) (2008) Integrated bio-behavioral surveillance for HIV infection among injecting drug users in Iran. Draft of the 1st analysis on the collected data, Tehran, Iran.

51. Osooli M, Khajehkazemi R, Sajadi L, Sedaghat A, Fahimfar N, et al. (2012) HIV prevalence and risk behaviors of adult male injection drug users in Iran; a 2010 national surveillance survey. In press.

52. Vazirian M, Nassirimanesh B, Zamani S, Ono-Kihara M, Kihara M, et al. (2005) Needle and syringe sharing practices of injecting drug users participating in an outreach HIV prevention program in Tehran, Iran: a cross-sectional study. Harm Reduct J 2: 19.

53. Mojtahedzadeh V, Razani N, Malekinejad M, Vazirian M, Shoaee S, et al. (2008) Injection drug use in Rural Iran: integrating HIV prevention into iran's rural primary health care system. AIDS Behav 12: S7-12.

54. Alavi SM, Alavi L, Jaafari F (2010) Outbreak investigation of needle sharing-induced malaria, Ahvaz, Iran. Int J Infect Dis 14: e240-242.

55. Morocco Ministry of Health, National Aids Control Program, National Institute of Hygiene, UNAIDS, Global Fund to Fight AIDS Tuberculosis and Malaria (2012) HIV Integrated Behavioral and Biological Surveillance Surveys-Morocco 2011-2012: Injecting Drug Users in Tanger and Nador, Morocco. Rabat, Morocco.

56. Hadi D.H.M.H, Shujaat P.D.M.G.S.H, Waheed P.D.W.u.Z, Masood P.D.M.G.M.A (2005) Incidence of hepatitis C virus and HIV among injecting drug users in Northern Pakistan: a prospective cohort study. Abstract no. MoOa0104. IAS 2005 - The 3rd IAS Conference on HIV Pathogenesis and Treatment Rio de Janeiro, Brazil.

57. Heidari AR, Mirahmadizadeh AR, Keshtkaran A, Javanbakht M, Etemad K, et al. (2011) Changes in unprotected sexual behavior and shared syringe use among addicts referring to Methadone Maintenance Treatment [MMT] centers affiliated to Shiraz University of Medical Sciences in Shiraz, Iran: an uncontrolled interventional study [Persian]. J Sch Public Health Inst Public Health Res 9: 67-76.

58. Mahfoud Z, Afifi R, Ramia S, El Khoury D, Kassak K, et al. (2010) HIV/AIDS among female sex workers, injecting drug users and men who have sex with men in Lebanon: results of the first biobehavioral surveys. AIDS 24 Suppl 2: S45-54.

59. Palestine Ministry of Health (2011) HIV bio-behavioral survey among injecting drug users in the East Jerusalem Governorate, 2010.

60. Stulhofer A, Chetty A, Rabie RA, Jwehan I, Ramlawi A (2012) The Prevalence of HIV, HBV, HCV, and HIV-Related Risk-Taking Behaviors among Palestinian Injecting Drug Users in the East Jerusalem Governorate. J Urban Health 89: 671-676.

61. Pakistan National AIDS Control Program (2008) HIV Second Generation Surveillance In Pakistan. National Report Round III. Canada-Pakistan HIV/AIDS Surveillance Project. National Aids Control Program, Ministry Of Health, Pakistan. Found at <http://www.nacp.gov.pk/library/reports/Surveillance%20&%20Research/HIV-AIDS%20Surveillance%20Project-HASP/HIV%20Second%20Generation%20Surveillance%20in%20Pakistan%20-%20National%20report%20Round%20III%202008.pdf>, Last accessed February 2014.

62. Bokhari A, Nizamani NM, Jackson DJ, Rehan NE, Rahman M, et al. (2007) HIV risk in Karachi and Lahore, Pakistan: an emerging epidemic in injecting and commercial sex networks. Int J STD AIDS 18: 486-492.

63. Pakistan National AIDS Control Program (2006-07) HIV Second Generation Surveillance In Pakistan. National Report Round II. Canada-Pakistan HIV/AIDS Surveillance Project. National Aids Control Program, Ministry Of Health, Pakistan. Found at <http://www.nacp.gov.pk/library/reports/Surveillance%20&%20Research/HIV-AIDS%20Surveillance%20Project-HASP/HIV%20Second%20Generation%20Surveillance%20in%20Pakistan%20-%20Round%202%20Report%202006-07.pdf>. Last accessed February 2014.

64. Pakistan National AIDS Control Program (2011) HIV Second Generation Surveillance In Pakistan. National Report Round IV. Canada-Pakistan HIV/AIDS Surveillance Project. National Aids Control Program, Ministry Of Health, Pakistan. Found at <http://www.nacp.gov.pk/library/reports/Surveillance%20&%20Research/HIV-AIDS%20Surveillance%20Project-HASP/HIV%20Second%20Generation%20Surveillance%20in%20Pakistan%20-%20National%20report%20Round%20IV%202011.pdf>, Last accessed February 2014.

65. Pakistan National AIDS Control Program (2005) Integrated biological and behavioral surveillance: A Pilot study in Karachi & Rawalpindi 2004-5. Canada-Pakistan HIV/AIDS Surveillance Project. National Aids Control Program, Ministry Of Health, Pakistan. Found at <http://www.nacp.gov.pk/library/reports//Surveillance%20&%20Research//HIV-AIDS%20Surveillance%20Project-HASP/Integrated%20Biological%20&%20Behavioral%20Surveillance%20Pilot%20Study%20in%20Karachi%20&%20Rawalpindi%202005-06.pdf>, Last accessed February 2014.

66. Pakistan National AIDS Control Program (2005) HIV Second Generation Surveillance In Pakistan. National Report Round I. Canada-Pakistan HIV/AIDS Surveillance Project. National Aids Control Program, Ministry Of Health, Pakistan. Found at <http://www.nacp.gov.pk/library/reports/Surveillance%20&%20Research/HIV-AIDS%20Surveillance%20Project-HASP/HIV%20Second%20Generation%20Surveillance%20in%20Pakistan%20-%20Round%201%20Report%20-%202005.pdf>, Last accessed February 2014.

67. Zafar T, Brahmbhatt H, Imam G, ul Hassan S, Strathdee SA (2003) HIV knowledge and risk behaviors among Pakistani and Afghani drug users in Quetta, Pakistan. J Acquir Immune Defic Syndr 32: 394-398.

68. Ahmed MA, Zafar T, Brahmbhatt H, Imam G, Ul Hassan S, et al. (2003) HIV/AIDS risk behaviors and correlates of injection drug use among drug users in Pakistan. J Urban Health 80: 321-329.

69. Strathdee SA, Zafar T, Brahmbhatt H, Baksh A, ul Hassan S (2003) Rise in needle sharing among injection drug users in Pakistan during the Afghanistan war. Drug Alcohol Depend 71: 17-24.

70. Moutassem-Mimouni B, Benghabrit-Remaoun N (2006) A study on the potential link between the problematic use of drugs and HIV/AIDS in Algeria (French) [Etude du lien potentiel entre usage problématique de drogues et VIH/SIDA en Algérie]. Ministère de l'Enseignement Supérieur et de la Recherche Scientifique, Centre National de Recherche en Anthropologie Sociale et Culturelle. Alger, Algeria

71. Al-Haddad MK, Khashaba AS, Baig BZ, Khalfan S (1994) HIV antibodies among intravenous drug users in Bahrain. J Commun Dis 26: 127-132.

72. Salama, II, Kotb NK, Hemeda SA, Zaki F (1998) HIV/AIDS knowledge and attitudes among alcohol and drug abusers in Egypt. J Egypt Public Health Assoc 73: 479-500.

73. Attia, Medhat S (1996) HIV Seropositivity and KAP towards AIDS among drug addicts in Alexandria. Bull High Inst Public Health 26: 1-8.

74. Saleh E, El-Ghazzawi E, El-Sherbini I, Drew W, McFarland W, et al. (1998) Sentinel surveillance for HIV and high risk behaviors among injection drug users in Alexandria, Egypt. Abstract no. 13124. AIDS 1998 - XII International AIDS Conference. Geneva, Switzerland.

75. Elsawy HF, Al-Kabash IM (2011) Risky behaviors of HIV infection among drug dependents in Egypt. European Psychiatry 26.

76. Dolan K, Salimi S, Nassirimanesh B, Mohsenifar S, Allsop D, et al. (2011) Characteristics of Iranian women seeking drug treatment. J Womens Health (Larchmt) 20: 1687-1691.

77. Eshrati B, Asl RT, Dell CA, Afshar P, Millson PME, et al. (2008) Preventing HIV transmission among Iranian prisoners: Initial support for providing education on the benefits of harm reduction practices. Harm Reduction Journal 5.

78. Zakizad M, Salmeh F, Yaghoobi T, Yaghoubian M, Nesami MB, et al. (2009) Seroprevalence of hepatitis C infection and associated risk factors among addicted prisoners in Sari-Iran. Pak J Biol Sci 12: 1012-1018.

79. Shoghli AR, Mosavi Nasab SN, Fallahnezhad M, Momtazi S, Tavasoli SS, et al. (2011) Behavioral surveillance survey (BSS) among injection drug users (IDUs) in Zanjan-Iran. [Persian]. Journal of Zanjan University of Medical Sciences and Health Services 19: 11.

80. Dastjerdi G, Ebrahimi Dehshiri V, Kholasezade G, Ehsani F (2010) Effectiveness of Methadone in Reduction of High Risk Behaviors in Clients of MMT Center [Persian]. Journal of Shaheed Sadoughi University of Medical Sciences 18: 215-219.

81. Alizadeh AH, Alavian SM, Jafari K, Yazdi N (2005) Prevalence of hepatitis C virus infection and its related risk factors in drug abuser prisoners in Hamedan--Iran. World J Gastroenterol 11: 4085-4089.

82. Ghasemian R, Najafi N, Amirkhanloo K (2011) The study of infections due to injection drug abuse in the injecting drug users hospitalized at Imam Khomeini Hospital in Sari and Razi Hospital in Ghaemshahr in 2007-2009. Journal of Mazandaran University of Medical Sciences 21: 8-15.

83. Pourahmad M, Javady A, Karimi I, Ataei B, Kassaeian N (2007) Seroprevalence of and risk factors associated with hepatitis B, hepatitis C, and human immunodeficiency virus among prisoners in Iran. Infectious Diseases in Clinical Practice 15: 368-372.

84. Alaei K, Alaei A, Mansoori D, Tabar, Heravi, et al. (2002) The epidemiological status of IDU and HIV infection in addict cases submitted to HIV/STI/IDU Counseling and Care Center in Kermanshah province. Abstract no. LbPp2211. AIDS 2002 - XIV International AIDS Conference. Barcelona, Spain.

85. United Nations Office on Drugs and Crime (UNODC), Arab World for Research and Development, Palestinian Authority (2011) Situation assessment of drug use and HIV among drug users in the West Bank and Gaza strip.

86. Farooq S, Akhtar J, Azeemi MH, Nazar Z, Khan SA (2006) Sociodemographic and clinical characteristics of IV drug users presenting to a tertiary care treatment centre. Journal of Postgraduate Medical Institute 20: 3-7.

87. Kazi A, Shah S, Jenkins C, Altaf A, Vermund S (2010) High risk behaviors related to sexual and drug use among prisoners in Pakistan. Abstract no. TUPE0274. AIDS 2010 - XVIII International AIDS Conference. Vienna, Austria.

88. Baqi S, Nabi N, Hasan SN, Khan AJ, Pasha O, et al. (1998) HIV antibody seroprevalence and associated risk factors in sex workers, drug users, and prisoners in Sindh, Pakistan. J Acquir Immune Defic Syndr Hum Retrovirol 18: 73-79.

89. Akhtar A, Aslam M, Zafar M (2004) Determinants of HIV risky behaviors among drug users in Faisalabad Pakistan. Abstract no. C11530. AIDS 2004 - XV International AIDS Conference Bangkok, Thailand.

90. UrRehman N (2002) Injecting drug use and HIV/AIDS in pakistan. Abstract no. MoPeD3667. AIDS 2002 - XIV International AIDS Conference. Barcelona, Spain.

91. Ur Rehman L, Ullah I, Ali I, Khan IA, Iqbal A, et al. (2011) Active hepatitis C infection and HCV genotypes prevalent among the IDUs of Khyber Pakhtunkhwa. Virology Journal 8.

92. Nai Zindagi, UNODCCP, UNAIDS. (1999) Baseline study of the relationship between injecting drug use, HIV and Hepatitis C among male injecting drug users in Lahore.

93. Emmanuel F, Akhtar S, Attarad A, Kamran C (2004) HIV risk behavior and practices among heroin addicts in Lahore, Pakistan. Southeast Asian J Trop Med Public Health 35: 940-948.

94. Tunisia Ministry of Health, Tunisian Association for Information and Orientation on HIV (2013) Enquête sérocomportementale du VIH et des hépatites virales C auprès des usagers de drogues injectables en Tunisie [French]. Biobehavioral surveillance of HIV and Hepatitis C among injecting drug users in Tunisia. Tunis, Tunisia.

95. Asadi S, Marjani M (2006) Prevalence of intravenous drug use-associated infections. Iranian Journal of Clinical Infectious Diseases 1: 59-62.

96. Altaf A, Shah SA, Zaidi NA, Memon A, Nadeem ur R, et al. (2007) High risk behaviors of injection drug users registered with harm reduction programme in Karachi, Pakistan. Harm Reduct J 4: 7.

97. Alavian SM, Mirahmadizadeh A, Javanbakht M, Keshtkaran A, Heidari A, et al. (2013) Effectiveness of methadone maintenance treatment in prevention of hepatitis C virus transmission among injecting drug users. Hepatitis Monthly 13: 9.

98. Mirahmadizadeh AR, Majdzadeh R, Mohammad K, MH F (2009) Prevalence of HIV and Hepatitis C Virus Infections and Related Behavioral Determinants among Injecting Drug Users of Drop-in Centers in Iran. Iranian Red Crescent Medical Journal 11: 325-329.

99. Nokhodian Z, Meshkati M, Adibi P, Ataei B, Kassaian N, et al. (2012) Hepatitis C among intravenous drug users in Isfahan, Iran: A study of seroprevalence and risk factors. International Journal of Preventive Medicine 3: S131-138.

100. Ahmad S, Mehmood J, Awan AB, Zafar ST, Khoshnood K, et al. (2011) Female spouses of injection drug users in Pakistan: a bridge population of the HIV epidemic? East Mediterr Health J 17: 271-276.

101. Pakistan National AIDS Control Programe (2005) National Study of Reproductive Tract and Sexually Transmitted Infections. Survey of High Risk Groups in Lahore and Karachi. Ministry of Health, Pakistan.

102. Mirzazadeh A, Navadeh S, Kamali K, Fahimfar N, Azadmanesh K, et al. (2010) High risk injection amongst male prisoners, before and during captivity; a national bio-behavioral survey. Abstract no. TUPE0461. AIDS 2010 - XVIII International AIDS Conference. Vienna, Austria.

103. Zamani S, Farnia M, Torknejad A, Alaei BA, Gholizadeh M, et al. (2010) Patterns of drug use and HIV-related risk behaviors among incarcerated people in a prison in Iran. J Urban Health 87: 603-616.

104. Alipour A, Haghdoost AA, Sajadi L, Zolala F (2013) HIV prevalence and related risk behaviours among female partners of male injecting drugs users in Iran: results of a bio-behavioural survey, 2010. Sex Transm Infect 89 Suppl 3: iii41-44.

105. Achakzai M, Kassi M, Kasi PM (2007) Seroprevalences and co-infections of HIV, hepatitis C virus and hepatitis B virus in injecting drug users in Quetta, Pakistan. Trop Doct 37: 43-45.

106. Afghanistan National AIDS Control Program (2012) Integrated Behavioral & Biological Surveillance (IBBS) in selected cities of Afghanistan: Findings of 2012 IBBS survey and comparison to 2009 IBBS survey. Johns Hopkins University School of Public Health, National AIDS Control Program, Ministry of Public Health. Kabul, Afghanistan.

107. Aminzadeh Z (2007) Seroepidemiology of HIV, syphilis, hepatitis B and C in intravenous drug users at Loghman Hakim hospital]. Iran J Med Meicrobiol 1: 53-56.

108. Zali MR, Aghazadeh R, Nowroozi A, Amir-Rasouly H (2001) Anti-HCV antibody among Iranian IV drug users: is it a serious problem. Arch Iranian Med 4: 115-119.
