# Supplementary material for: HIV among People Who Inject Drugs in the Middle East and North Africa: Systematic Review and Data Synthesis
Source: PLoS Med. 2014 Jun 17;11(6):e1001663. doi: 10.1371/journal.pmed.1001663 (PMC4061009; doi:10.1371/journal.pmed.1001663)
Supplement: Table S6 — Measures of sexual risk behavior and sexually transmitted infections prevalence among people who inject drugs in the Middle East and North Africa. (DOCX) [file pmed.1001663.s006.docx]

**Table S6. Measures of sexual risk behavior and sexually transmitted infections prevalence among people who inject drugs in the Middle East and North Africa**

|  | **Afghanistan** | **Bahrain** | **Egypt** | **Iran** | **Jordan** | **Lebanon** | **Libya** | **Morocco** | **Oman** | **OPT** | **Pakistan** | **Syria** | **Tunisia** |
| --- | --- | --- | --- | --- | --- | --- | --- | --- | --- | --- | --- | --- | --- |
| **Sexually active (%)** |  |  |  |  |  |  |  |  |  |  |  |  |  |
| Currently |  |  | 30 [[1](#_ENREF_1)], 73 [[2](#_ENREF_2)] | 95 [[3](#_ENREF_3)] |  |  |  |  |  |  |  |  |  |
| Ever | 83 [[4](#_ENREF_4)], 86 [[4](#_ENREF_4)], 92 [[5](#_ENREF_5)], 93 [[4](#_ENREF_4)], 94 [[4](#_ENREF_4)] | 91 [[6](#_ENREF_6)] | 95 [[7](#_ENREF_7)], 96 [[7](#_ENREF_7)], 96 [[8](#_ENREF_8)] | 75 [[9](#_ENREF_9)], 77 [[10](#_ENREF_10)], 81 [[11](#_ENREF_11)], 84 [[12](#_ENREF_12)], 85 [[13](#_ENREF_13)], 86 [[14](#_ENREF_14)], 87 [[14](#_ENREF_14)], 88 [[14](#_ENREF_14)] |  |  | 77 [[15](#_ENREF_15)] |  | 91 [[16](#_ENREF_16)], 94 [[16](#_ENREF_16)], 100 [[16](#_ENREF_16)] |  | 77 [[17](#_ENREF_17)], 79 [[17](#_ENREF_17)], 84 [[18](#_ENREF_18)], 89 [[19](#_ENREF_19)], 89 [[20](#_ENREF_20)], 93 [[21](#_ENREF_21)], 94 [[19](#_ENREF_19)], 95 [[22](#_ENREF_22)] |  | 96 [[23](#_ENREF_23)], 97 [[24](#_ENREF_24)] |
| Last year |  |  | 71 [[8](#_ENREF_8)], 76 [[7](#_ENREF_7)], 80 [[7](#_ENREF_7)] | 39 [[25](#_ENREF_25)], 54 [[25](#_ENREF_25)] | 64 [[26](#_ENREF_26)] | 73 [[27](#_ENREF_27)] |  |  |  |  | 52 [[28](#_ENREF_28)] | 78 [[29](#_ENREF_29)] |  |
| Last 12 months |  |  |  |  |  |  |  | 55 [[30](#_ENREF_30)], 61 [[30](#_ENREF_30)] |  |  |  |  | 87 [[24](#_ENREF_24)] |
| Last 6 months | 58 [[31](#_ENREF_31)], 76 [[31](#_ENREF_31)], 78 [[31](#_ENREF_31)], 80 [[31](#_ENREF_31)], 85 [[31](#_ENREF_31)] |  |  |  |  |  |  |  |  |  | 22 [[32](#_ENREF_32)], 91 [[33](#_ENREF_33)] |  |  |
| Last month |  |  | 54 [[2](#_ENREF_2)] |  |  |  | 23 [[15](#_ENREF_15)] |  |  | 63 [[34](#_ENREF_34)] |  | 68 [[29](#_ENREF_29)] |  |
| **Married (%)** |  |  |  |  |  |  |  |  |  |  |  |  |  |
| Currently | 30 [[35](#_ENREF_35)], 34 [[35](#_ENREF_35)], 37 [[31](#_ENREF_31)], 39 [[31](#_ENREF_31)], 42 [[31](#_ENREF_31)], 43 [[35](#_ENREF_35)], 52 [[36](#_ENREF_36)], 53 [[31](#_ENREF_31)], 61 [[31](#_ENREF_31)], 68 [[37](#_ENREF_37)] | 27 [[6](#_ENREF_6)] | 29 [[7](#_ENREF_7)], 38 [[38](#_ENREF_38)], 39 [[8](#_ENREF_8)], 49 [[7](#_ENREF_7)] | 9 [[39](#_ENREF_39)], 26 [[40](#_ENREF_40)], 28 [[14](#_ENREF_14)], 29 [[41](#_ENREF_41)], 33 [[42](#_ENREF_42)], 33 [[43](#_ENREF_43)], 33 [[25](#_ENREF_25)], 33 [[14](#_ENREF_14)], 33 [[25](#_ENREF_25)], 35 [[44](#_ENREF_44)], 36 [[14](#_ENREF_14)], 39 [[45](#_ENREF_45)], 45 [[46](#_ENREF_46)], 45 [[47](#_ENREF_47)], 45 [[48](#_ENREF_48)], 48 [[49](#_ENREF_49)], 48 [[50](#_ENREF_50)], 52 [[51](#_ENREF_51)], 54 [[52](#_ENREF_52)], 56 [[53](#_ENREF_53)], 64 [[47](#_ENREF_47)], 67 [[54](#_ENREF_54)], 79 [[54](#_ENREF_54)], 89 [[55](#_ENREF_55)] | 49 [[26](#_ENREF_26)] | 21 [[56](#_ENREF_56)] | 12 [[15](#_ENREF_15)] | 19 [[57](#_ENREF_57)], 20 [[30](#_ENREF_30)], 21 [[30](#_ENREF_30)] | 3 [[16](#_ENREF_16)], 26 [[16](#_ENREF_16)], 28 [[16](#_ENREF_16)] | 67 [[58](#_ENREF_58)] | 24 [[59](#_ENREF_59)], 26 [[60](#_ENREF_60)], 26 [[17](#_ENREF_17)], 28 [[61](#_ENREF_61)], 30 [[32](#_ENREF_32)], 30 [[62](#_ENREF_62)], 31 [[28](#_ENREF_28)], 33 [[63](#_ENREF_63)], 34 [[21](#_ENREF_21)], 35 [[64](#_ENREF_64)], 41 [[22](#_ENREF_22)], 42 [[65](#_ENREF_65)], 45[[20](#_ENREF_20)], 45 [[63](#_ENREF_63)], 45 [[63](#_ENREF_63)], 45 [[18](#_ENREF_18)], 48 [[63](#_ENREF_63)], 53 [[66](#_ENREF_66)], 53 [[66](#_ENREF_66)], 55 [[33](#_ENREF_33)], 68 [[19](#_ENREF_19)], 73 [[67](#_ENREF_67)], 77 [[19](#_ENREF_19)] | 44 [[29](#_ENREF_29)] | 16 [[24](#_ENREF_24)] |
| Ever | 38 [[68](#_ENREF_68)], 46 [[5](#_ENREF_5)], 52 [[68](#_ENREF_68)], 64 [[69](#_ENREF_69)] |  | 34 [[7](#_ENREF_7)], 56 [[8](#_ENREF_8)], 56 [[7](#_ENREF_7)] | 19 [[70](#_ENREF_70)], 35 [[12](#_ENREF_12)], 43 [[71](#_ENREF_71)], 44 [[13](#_ENREF_13)], 52 [[72](#_ENREF_72)], 54 [[14](#_ENREF_14)], 54 [[39](#_ENREF_39)], 55 [[14](#_ENREF_14)], 55 [[73](#_ENREF_73)], 56 [[14](#_ENREF_14)], 57 [[11](#_ENREF_11)], 65 [[73](#_ENREF_73)] |  | 33 [[56](#_ENREF_56)] |  |  |  |  | 26 [[74](#_ENREF_74)], 43 [[74](#_ENREF_74)] |  | 20 [[24](#_ENREF_24)] |
| **Had sex with regular female partners (%)** |  |  |  |  |  |  |  |  |  |  |  |  |  |
| Last year |  |  | 83 [[7](#_ENREF_7)], 86 [[7](#_ENREF_7)], 89 [[8](#_ENREF_8)] | 43 [[14](#_ENREF_14)], 57 [[14](#_ENREF_14)], 58 [[14](#_ENREF_14)] |  | 66 [[56](#_ENREF_56)] |  |  |  |  |  |  |  |
| Last 6 months |  |  |  |  |  |  |  |  |  |  | 26 [[21](#_ENREF_21)], 38 [[22](#_ENREF_22)], 42 [[20](#_ENREF_20)], 46 [[18](#_ENREF_18)] |  |  |
| Last month |  |  |  |  |  | 62 [[75](#_ENREF_75)] | 73 [[15](#_ENREF_15)] |  |  |  |  |  | 77 [[24](#_ENREF_24)] |
| **Had sex with non-regular female partners (%)** |  |  |  |  |  |  |  |  |  |  |  |  |  |
| Last year |  |  | 29 [[8](#_ENREF_8)], 34 [[7](#_ENREF_7)], 42 [[7](#_ENREF_7)] | 27 [[14](#_ENREF_14)], 31 [[14](#_ENREF_14)], 34 [[14](#_ENREF_14)] |  | 40 [[56](#_ENREF_56)] |  | 18 [[30](#_ENREF_30)], 36 [[30](#_ENREF_30)] |  |  |  |  | 42 [[24](#_ENREF_24)] |
| Last month |  |  |  |  |  | 34 [[75](#_ENREF_75)] | 28 [[15](#_ENREF_15)] |  |  |  |  |  |  |
| Unspecified |  |  |  | 73 [[54](#_ENREF_54)] |  |  |  |  |  |  |  |  |  |
| **Had sex with men (%)** |  |  |  |  |  |  |  |  |  |  |  |  |  |
| Ever | 2 [[31](#_ENREF_31)], 10 [[5](#_ENREF_5)], 10 [[31](#_ENREF_31)], 10 [[4](#_ENREF_4)], 14 [[35](#_ENREF_35)], 15 [[35](#_ENREF_35)], 19 [[31](#_ENREF_31)], 19 [[31](#_ENREF_31)], 25 [[4](#_ENREF_4)], 26 [[35](#_ENREF_35)], 26 [[4](#_ENREF_4)], 29 [[31](#_ENREF_31)], 36 [[4](#_ENREF_4)] |  | 9 [[2](#_ENREF_2)], 18 [[38](#_ENREF_38)] | 5 [[45](#_ENREF_45)], 8 [[39](#_ENREF_39)], 8 [[11](#_ENREF_11)], 11 [[12](#_ENREF_12)], 13 [[76](#_ENREF_76)], 14 [[14](#_ENREF_14)], 17 [[14](#_ENREF_14)], 17 [[14](#_ENREF_14)], 18, [[49](#_ENREF_49)], 24 [[43](#_ENREF_43)], 27 [[51](#_ENREF_51)], 30 [[52](#_ENREF_52)], 36 [[9](#_ENREF_9)], 39 [[76](#_ENREF_76)] | 12 [[26](#_ENREF_26)] | 25 [[27](#_ENREF_27)] |  |  | 6 [[16](#_ENREF_16)], 12 [[16](#_ENREF_16)], 15 [[16](#_ENREF_16)], 28 [[16](#_ENREF_16)], 42 [[16](#_ENREF_16)], 35 [[16](#_ENREF_16)] | 25 [[58](#_ENREF_58)] | 37 [[19](#_ENREF_19)], 50 [[19](#_ENREF_19)] |  |  |
| Last 5 years |  |  |  |  |  |  |  |  |  |  | 7 [[32](#_ENREF_32)] |  |  |
| Last 12 months |  |  | 8 [[7](#_ENREF_7)], 9 [[8](#_ENREF_8)], 14 [[7](#_ENREF_7)] | 2 [[25](#_ENREF_25)], 2 [[25](#_ENREF_25)], 7 [[14](#_ENREF_14)], 8 [[14](#_ENREF_14)], 10 [[14](#_ENREF_14)] | 7 [[26](#_ENREF_26)] |  |  |  |  |  | 2 [[77](#_ENREF_77)], 14 [[77](#_ENREF_77)] | 4 [[29](#_ENREF_29)] |  |
| Last 6 months |  |  |  |  |  |  |  |  |  |  | 18 [[17](#_ENREF_17)], 27 [[17](#_ENREF_17)] |  |  |
| Last month |  |  |  | 19 [[44](#_ENREF_44)] |  | 10 [[27](#_ENREF_27)] |  |  |  |  |  |  |  |
| Unknown/unspecified |  | 9 [[6](#_ENREF_6)] |  | 12 [[3](#_ENREF_3)], 14 [[78](#_ENREF_78)] |  |  |  | 14 [[57](#_ENREF_57)] |  |  | 24 [[28](#_ENREF_28)] |  |  |
| **Had sex with a sex worker (%)** |  |  |  |  |  |  |  |  |  |  |  |  |  |
| Ever | 23 [[4](#_ENREF_4)], 37 [[5](#_ENREF_5)], 38 [[4](#_ENREF_4)], 47 [[35](#_ENREF_35)] 56 [[35](#_ENREF_35)], 64 [[35](#_ENREF_35)], 67 [[4](#_ENREF_4)], 72 [[4](#_ENREF_4)] |  | 13 [[8](#_ENREF_8)] | 23 [[45](#_ENREF_45)], 25 [[12](#_ENREF_12)], 32 [[13](#_ENREF_13)], 41 [[39](#_ENREF_39)] |  | 50 [[56](#_ENREF_56)] |  |  | 31 [[16](#_ENREF_16)], 40 [[16](#_ENREF_16)], 54 [[16](#_ENREF_16)] , 60 [[16](#_ENREF_16)], 65 [[16](#_ENREF_16)], 97 [[16](#_ENREF_16)] | 18 [[34](#_ENREF_34)] | 41 [[79](#_ENREF_79)], 43 [[62](#_ENREF_62)], 68 [[19](#_ENREF_19)], 71 [[19](#_ENREF_19)], 81 [[17](#_ENREF_17)], 18 [[17](#_ENREF_17)] | 47 [[29](#_ENREF_29)] |  |
| Last 12 months |  |  |  | 9 [[25](#_ENREF_25)], 15 [[25](#_ENREF_25)] | 30 [[26](#_ENREF_26)] |  |  |  |  |  | 21 [[77](#_ENREF_77)], 18 [[74](#_ENREF_74)], 23 [[74](#_ENREF_74)], 24 [[77](#_ENREF_77)], 30 [[74](#_ENREF_74)], 34 [[74](#_ENREF_74)] |  |  |
| Last 6 months | 5 [[68](#_ENREF_68)], 6 [[68](#_ENREF_68)], 19 [[68](#_ENREF_68)] |  |  |  |  |  |  |  |  |  | 7 [[21](#_ENREF_21)], 8 [[61](#_ENREF_61)], 13[[18](#_ENREF_18)], 13 [[61](#_ENREF_61)], 13 [[61](#_ENREF_61)], 14 [[21](#_ENREF_21)], 14 [[22](#_ENREF_22)], 15 [[20](#_ENREF_20)], 18 [[22](#_ENREF_22)], 27 [[18](#_ENREF_18)] 27 [[61](#_ENREF_61)], 28 [[61](#_ENREF_61)] |  |  |
| Last month |  |  |  |  |  | 33 [[75](#_ENREF_75)] | 20 [[15](#_ENREF_15)] |  |  |  | 11 [[67](#_ENREF_67)], 47 [[67](#_ENREF_67)] | 40 [[29](#_ENREF_29)] |  |
| Unknown/unspecified |  |  |  |  |  |  |  |  |  |  | 3 [[28](#_ENREF_28)], 96 [[28](#_ENREF_28)] |  |  |
| **Sold sex (%)** |  |  |  |  |  |  |  |  |  |  |  |  |  |
| Ever |  |  |  | 8 [[39](#_ENREF_39)], 36 [[14](#_ENREF_14)], 43 [[14](#_ENREF_14)], 45 [[14](#_ENREF_14)] |  | 12 [[56](#_ENREF_56)], 25 [[27](#_ENREF_27)] |  |  |  |  |  |  |  |
| Last 12 months |  |  | 26 [[38](#_ENREF_38)] | 18 [[14](#_ENREF_14)], 22 [[14](#_ENREF_14)] 26 [[14](#_ENREF_14)], 29 [[78](#_ENREF_78)] |  |  |  | 18 [[30](#_ENREF_30)], 24 [[30](#_ENREF_30)] |  | 8 [[34](#_ENREF_34)] | 5 [[74](#_ENREF_74)], 11 [[74](#_ENREF_74)] |  |  |
| Last 6 months |  |  |  |  |  |  |  |  |  |  | 15 [[21](#_ENREF_21)], 17 [[22](#_ENREF_22)], 20 [[18](#_ENREF_18)] |  |  |
| Last month |  |  |  | 19 [[80](#_ENREF_80)] |  |  |  |  |  |  |  |  |  |
| Unknown/unspecified |  |  |  |  |  |  |  |  |  |  | 9 [[66](#_ENREF_66)], 14 [[61](#_ENREF_61)], 19 [[28](#_ENREF_28)], 20 [[61](#_ENREF_61)] |  |  |
| **Sold or bought sex (%)** |  |  |  |  |  |  |  |  |  |  |  |  |  |
| Ever |  |  | 51 [[2](#_ENREF_2)] |  |  |  |  |  |  |  |  |  |  |
| Last 12 months |  |  | 11 [[7](#_ENREF_7)], 13 [[7](#_ENREF_7)] |  |  |  |  |  |  | 25 [[58](#_ENREF_58)] |  |  | 26 [[24](#_ENREF_24)] |
| Unknown/unspecified |  |  |  |  |  |  |  | 68 [[57](#_ENREF_57)] |  |  |  |  |  |
| **Have multiple partners (%)** |  |  |  |  |  |  |  |  |  |  |  |  |  |
| Lifetime |  |  |  | 54 [[11](#_ENREF_11)], 57 [[14](#_ENREF_14)], 59 [[14](#_ENREF_14)], 62 [[14](#_ENREF_14)] |  |  |  |  |  |  |  |  |  |
| Last 12 months |  |  | 39 [[7](#_ENREF_7)], 45 [[7](#_ENREF_7)] |  | 46 [[26](#_ENREF_26)] |  |  | 51 [[57](#_ENREF_57)] |  | 29 [[58](#_ENREF_58)] | 51 [[28](#_ENREF_28)] |  | 60 [[24](#_ENREF_24)] |
| Last 6 months |  |  |  |  |  |  |  |  |  |  | 18 [[33](#_ENREF_33)] |  |  |
| Last month |  |  | 49 [[2](#_ENREF_2)] |  |  |  | 23 [[15](#_ENREF_15)] |  |  |  |  |  |  |
| Unknown/unspecified |  |  |  | 41 [[47](#_ENREF_47)] |  |  |  |  |  |  |  |  |  |
| **Overall condom use (%)** |  |  |  |  |  |  |  |  |  |  |  |  |  |
| Ever | 10 [[4](#_ENREF_4)],16 [[4](#_ENREF_4)], 19 [[4](#_ENREF_4)],30 [[4](#_ENREF_4)], 33 [[4](#_ENREF_4)] |  | 41 [[2](#_ENREF_2)] | 24 [[51](#_ENREF_51)], 24 [[25](#_ENREF_25)], 29 [[25](#_ENREF_25)], 53 [[11](#_ENREF_11)], 53 [[13](#_ENREF_13)], 57 [[9](#_ENREF_9)] |  | 88 [[27](#_ENREF_27)] |  | 55 [[57](#_ENREF_57)] | 53 [[16](#_ENREF_16)], 63[[16](#_ENREF_16)], 69[[16](#_ENREF_16)] |  | 14 [[19](#_ENREF_19)], 16 [[79](#_ENREF_79)], 18 [[28](#_ENREF_28)], 21 [[62](#_ENREF_62)], 36 [[67](#_ENREF_67)], 37 [[17](#_ENREF_17)], 38 [[19](#_ENREF_19)] | 60 [[29](#_ENREF_29)] |  |
| Last 12 months |  |  |  |  |  |  |  | 24 [[30](#_ENREF_30)], 36 [[30](#_ENREF_30)] |  |  |  |  |  |
| Last 6 months |  |  |  |  |  |  |  |  |  |  | 33 [[32](#_ENREF_32)] |  |  |
| Last act | 4 [[31](#_ENREF_31)], 15 [[31](#_ENREF_31)], 17 [[35](#_ENREF_35)],18 [[31](#_ENREF_31)], 20 [[31](#_ENREF_31)], 26 [[35](#_ENREF_35)], 27 [[31](#_ENREF_31)], 32 [[35](#_ENREF_35)] |  |  | 38 [[80](#_ENREF_80)] | 7 [[81](#_ENREF_81)] |  | 66 [[15](#_ENREF_15)] |  |  | 34 [[58](#_ENREF_58)] |  |  | 24 [[24](#_ENREF_24)], 34 [[23](#_ENREF_23)] |
| Unknown/unspecified |  |  | 9 [[82](#_ENREF_82)] | 68 [[55](#_ENREF_55)] |  |  |  |  |  |  |  |  |  |
| **Consistent condom use (%)** |  |  |  |  |  |  |  |  |  |  |  |  |  |
| Last 12 months |  |  | 14 [[2](#_ENREF_2)] |  |  |  |  |  | 12 [[16](#_ENREF_16)], 19 [[16](#_ENREF_16)], 25 [[16](#_ENREF_16)] |  |  | 19 [[29](#_ENREF_29)] |  |
| Last 6 months |  |  |  |  |  |  |  |  |  |  | 7 [[32](#_ENREF_32)] |  |  |
| Last month |  |  |  |  |  |  |  |  |  | 30 [[58](#_ENREF_58)] |  |  |  |
| Unknown/unspecified |  |  |  |  |  |  |  |  |  |  | 9 [[32](#_ENREF_32)] |  |  |
| **Condom use with commercial sex workers (%)** |  |  |  |  |  |  |  |  |  |  |  |  |  |
| Ever | 18 [[4](#_ENREF_4)], 19 [[4](#_ENREF_4)], 34 [[4](#_ENREF_4)], 39 [[4](#_ENREF_4)] |  |  |  | 65 [[26](#_ENREF_26)] |  |  |  |  |  |  |  |  |
| Last 12 months |  |  | 34 [[8](#_ENREF_8)] | 24 [[25](#_ENREF_25)], 24 [[25](#_ENREF_25)] |  |  |  |  |  |  |  |  |  |
| Last 6 months |  |  |  |  |  |  |  |  |  |  | 7 [[61](#_ENREF_61)], 49 [[61](#_ENREF_61)] |  |  |
| Last month |  |  |  |  |  |  |  |  | 67 [[16](#_ENREF_16)], 75 [[16](#_ENREF_16)], 75 [[16](#_ENREF_16)] |  |  | 49 [[29](#_ENREF_29)] |  |
| Last act |  |  |  |  |  |  | 80 [[15](#_ENREF_15)] |  |  | 48 [[34](#_ENREF_34)] | 17 [[20](#_ENREF_20)], 17 [[74](#_ENREF_74)], 21 [[18](#_ENREF_18)], 28 [[21](#_ENREF_21)], 31 [[22](#_ENREF_22)], 32 [[74](#_ENREF_74)] |  | 29 [[24](#_ENREF_24)], 37 [[23](#_ENREF_23)] |
| **Condom use during anal sex with male (%)** |  |  |  |  |  |  |  |  |  |  |  |  |  |
| Ever | 3 [[4](#_ENREF_4)], 4 [[83](#_ENREF_83)], 13 [[83](#_ENREF_83)], 13 [[83](#_ENREF_83)] |  |  |  |  |  |  |  |  |  |  |  |  |
| Last 12 months |  |  |  | 0 [[25](#_ENREF_25)], 33 [[25](#_ENREF_25)] |  |  |  |  | 0 [[16](#_ENREF_16)], 40 [[16](#_ENREF_16)], 42 [[16](#_ENREF_16)] |  |  | 0 [[29](#_ENREF_29)] |  |
| Last act |  |  |  | 6 [[14](#_ENREF_14)], 8 [[14](#_ENREF_14)], 15 [[14](#_ENREF_14)] |  |  |  |  |  |  |  |  |  |
| Prevalence of: |  |  |  |  |  |  |  |  |  |  |  |  |  |
| **Syphilis (%)** | 0.0 [[4](#_ENREF_4)], 1.2 [[5](#_ENREF_5)], 1.2 [[4](#_ENREF_4)], 1.9 [[35](#_ENREF_35)], 2.2 [[4](#_ENREF_4)], 3.3 [[31](#_ENREF_31)], 3.5 [[35](#_ENREF_35)], 3.8 [[31](#_ENREF_31)], 4.0 [[31](#_ENREF_31)], 4.0 [[31](#_ENREF_31)], 6.2 [[31](#_ENREF_31)], 6.9 [[31](#_ENREF_31)], 13.9 [[4](#_ENREF_4)], 16.7 [[35](#_ENREF_35)] |  | 3.0 [[84](#_ENREF_84)] | 0.0 [[85](#_ENREF_85)], 8.0 [[86](#_ENREF_86)] |  |  |  |  |  |  | 1.2 [[87](#_ENREF_87)], 3.9 [[77](#_ENREF_77)], 7.6 [[77](#_ENREF_77)], 13.1 [[67](#_ENREF_67)], 14.0 [[88](#_ENREF_88)], 16.9 [[17](#_ENREF_17)], 18.2 [[88](#_ENREF_88)] |  |  |
| **HSV-2 (%)** | 4.4 [[35](#_ENREF_35)], 7.7 [[35](#_ENREF_35)], 20.6 [[35](#_ENREF_35)] |  |  |  |  |  |  |  |  |  | 6.0 [[77](#_ENREF_77)], 11.0 [[77](#_ENREF_77)], 19.0 [[87](#_ENREF_87)] |  |  |
| **Gonorrhea (%)** |  |  |  |  |  |  |  |  |  |  | 0.0 [[77](#_ENREF_77)], 1.0 [[88](#_ENREF_88)], 1.3 [[77](#_ENREF_77)], 1.8 [[88](#_ENREF_88)], 12.8 [[87](#_ENREF_87)] |  |  |
| **Chlamydia (%)** |  |  |  |  |  |  |  |  |  |  | 0.0 [[77](#_ENREF_77)], 0.2 [[88](#_ENREF_88)], 0.5 [[88](#_ENREF_88)], 0.7 [[77](#_ENREF_77)] |  |  |
| **Self-reported history of sexually transmitted disease (%)** |  |  | 11.0 [[82](#_ENREF_82)] | 7.6 [[45](#_ENREF_45)], 22.8 [[47](#_ENREF_47)] |  |  |  |  |  |  | 6.8 [[20](#_ENREF_20)], 10.0 [[17](#_ENREF_17)], 10.4 [[18](#_ENREF_18)], 11.4 [[22](#_ENREF_22)], 12.1 [[79](#_ENREF_79)], 18.8 [[17](#_ENREF_17)], 23.0 [[89](#_ENREF_89)], 54.1 [[19](#_ENREF_19)], 65.9 [[19](#_ENREF_19)] |  |  |

OPT: Occupied Palestinian Territories

NB: The denominator in some studies corresponds to sexual active PWID while in other studies the denominator corresponds to the total sample of PWID in the study.

**References**

1. Hasan M, Farag A, Ismail M (1994) AIDS and intravenous drug users in Egypt. Abstract no. PC0143. AIDS 1994 - X International AIDS Conference Yokohama, Japan.

2. Elshimi T, Warner-Smith M, Aon M (2004) Blood-borne virus risks of problematic drug users in Greater Cairo. Geneva, UNAIDS & UNODC. August.

3. Rowhani-Rahbar A, Tabatabaee-Yazdi A, Panahi M (2004) Prevalence of Common Blood-Borne Infections among Imprisoned Injection Drug Users in Mashhad, North-East of Iran. Archives of Iranian Medicine 7: 190-194.

4. Todd CS, Nasir A, Raza Stanekzai M, Abed AM, Strathdee SA, et al. (2010) Prevalence and correlates of syphilis and condom use among male injection drug users in four Afghan cities. Sex Transm Dis 37: 719-725.

5. Todd CS, Nasir A, Stanekzai MR, Fiekert K, Rasuli MZ, et al. (2011) Prevalence and correlates of HIV, syphilis, and hepatitis B and C infection and harm reduction program use among male injecting drug users in Kabul, Afghanistan: A cross-sectional assessment. Harm Reduct J 8: 22.

6. Al-Haddad MK, Khashaba AS, Baig BZ, Khalfan S (1994) HIV antibodies among intravenous drug users in Bahrain. J Commun Dis 26: 127-132.

7. Family Health International and Ministry of Health Egypt (2010) HIV/AIDS Biological & Behavioral Surveillance Survey: Round Two Summary Report, Cairo, Egypt 2010. FHI in collaboration with the Ministry of Health and support from the Global Fund. Found at <http://www.fhi360.org/sites/default/files/media/documents/BBSS%202010_0.pdf>, Last accessed February 2014.

8. Soliman C, Rahman IA, Shawky S, Bahaa T, Elkamhawi S, et al. (2010) HIV prevalence and risk behaviors of male injection drug users in Cairo, Egypt. AIDS 24 Suppl 2: S33-38.

9. Momtazi S, Fallahnejad M, Shoghli A, Musavinasab N, Tavassoli S (2010) HIV high risk behavior in a sample of Iranian injection drug users. Abstract no. TUPE0338. AIDS 2010 - XVIII International AIDS Conference. Vienna, Austria.

10. Kheirandish P, SeyedAlinaghi S, Jahani M, Shirzad H, Seyed Ahmadian M, et al. (2009) Prevalence and correlates of hepatitis C infection among male injection drug users in detention, Tehran, Iran. J Urban Health 86: 902-908.

11. Zamani S, Kihara M, Gouya MM, Vazirian M, Nassirimanesh B, et al. (2006) High prevalence of HIV infection associated with incarceration among community-based injecting drug users in Tehran, Iran. J Acquir Immune Defic Syndr 42: 342-346.

12. Zamani S, Radfar R, Nematollahi P, Fadaie R, Meshkati M, et al. (2010) Prevalence of HIV/HCV/HBV infections and drug-related risk behaviours amongst IDUs recruited through peer-driven sampling in Iran. Int J Drug Policy 21: 493-500.

13. Zamani S, Kihara M, Gouya MM, Vazirian M, Ono-Kihara M, et al. (2005) Prevalence of and factors associated with HIV-1 infection among drug users visiting treatment centers in Tehran, Iran. AIDS 19: 709-716.

14. Iran Ministry of Health and Medical Education , Kyoto University School of Public Hleath (Japan) (2008) Integrated bio-behavioral surveillance for HIV infection among injecting drug users in Iran. Draft of the 1st analysis on the collected data, Tehran, Iran.

15. Mirzoyan L, Berendes S, Jeffery C, Thomson J, Ben Othman H, et al. (2013) New evidence on the HIV epidemic in Libya: why countries must implement prevention programs among people who inject drugs. J Acquir Immune Defic Syndr 62: 577-583.

16. Oman Ministry of Health (2006) HIV Risk among Heroin and Injecting Drug Users in Muscat, Oman. Quantitative Survey. Preliminary Data. Muscat, Oman.

17. Altaf A, Shah SA, A. M (2003) Follow up study to assess and evaluate knowledge, attitude and high risk behaviors and prevalence of HIV, HBV, HCV and Syphilis among IDUS at Burns Road DIC, Karachi. External report submitted to UNODC.

18. Pakistan National AIDS Control Program (2006-07) HIV Second Generation Surveillance In Pakistan. National Report Round II. Canada-Pakistan HIV/AIDS Surveillance Project. National Aids Control Program, Ministry Of Health, Pakistan. Found at <http://www.nacp.gov.pk/library/reports/Surveillance%20&%20Research/HIV-AIDS%20Surveillance%20Project-HASP/HIV%20Second%20Generation%20Surveillance%20in%20Pakistan%20-%20Round%202%20Report%202006-07.pdf>. Last accessed February 2014.

19. Kuo I, ul-Hasan S, Galai N, Thomas DL, Zafar T, et al. (2006) High HCV seroprevalence and HIV drug use risk behaviors among injection drug users in Pakistan. Harm Reduct J 3: 26.

20. Pakistan National AIDS Control Program (2005) HIV Second Generation Surveillance In Pakistan. National Report Round I. Canada-Pakistan HIV/AIDS Surveillance Project. National Aids Control Program, Ministry Of Health, Pakistan. Found at <http://www.nacp.gov.pk/library/reports/Surveillance%20&%20Research/HIV-AIDS%20Surveillance%20Project-HASP/HIV%20Second%20Generation%20Surveillance%20in%20Pakistan%20-%20Round%201%20Report%20-%202005.pdf>, Last accessed February 2014.

21. Pakistan National AIDS Control Program (2011) HIV Second Generation Surveillance In Pakistan. National Report Round IV. Canada-Pakistan HIV/AIDS Surveillance Project. National Aids Control Program, Ministry Of Health, Pakistan. Found at <http://www.nacp.gov.pk/library/reports/Surveillance%20&%20Research/HIV-AIDS%20Surveillance%20Project-HASP/HIV%20Second%20Generation%20Surveillance%20in%20Pakistan%20-%20National%20report%20Round%20IV%202011.pdf>, Last accessed February 2014.

22. Pakistan National AIDS Control Program (2008) HIV Second Generation Surveillance In Pakistan. National Report Round III. Canada-Pakistan HIV/AIDS Surveillance Project. National Aids Control Program, Ministry Of Health, Pakistan. Found at <http://www.nacp.gov.pk/library/reports/Surveillance%20&%20Research/HIV-AIDS%20Surveillance%20Project-HASP/HIV%20Second%20Generation%20Surveillance%20in%20Pakistan%20-%20National%20report%20Round%20III%202008.pdf>, Last accessed February 2014.

23. Tunisia Ministry of Health (2010) Synthèse des enquêtes de séroprévalence et sérocomportementales auprès de trois populations à vulnérables au VIH : Les usagers de drogues injectables, les hommes ayant des rapports sexuels avec des hommes et les travailleuses du sexe clandestines en Tunisie [French]. Synthesis of biobehavioral surveillance among the three populations vulnerable to HIV in Tunisia: Injecting drug users, men who have sex with men, and female sex workers. Tunis, Tunisia.

24. Tunisia Ministry of Health, Tunisian Association for Information and Orientation on HIV (2013) Enquête sérocomportementale du VIH et des hépatites virales C auprès des usagers de drogues injectables en Tunisie [French]. Biobehavioral surveillance of HIV and Hepatitis C among injecting drug users in Tunisia. Tunis, Tunisia.

25. Farhoudi B, Montevalian A, Motamedi M, Khameneh MM, Mohraz M, et al. (2003) Human immunodeficiency virus and HIV - associated tuberculosis infection and their risk factors in injecting drug users in prison in Iran. Iran Ministry of Health, Tehran, Iran.

26. Jordan National AIDS Program (2010) Preliminary analysis of Jordan IBBSS among injecting drug users. Ministry of Health, Amman, Jordan.

27. Aaraj E Report on the situation analysis on vulnerable groups in Beirut, Lebanon. Lebanon Ministry of Health, Beirut, Lebanon.

28. Nai Zindagi, UNODCCP, UNAIDS. (1999) Baseline study of the relationship between injecting drug use, HIV and Hepatitis C among male injecting drug users in Lahore.

29. Syria Mental Health Directorate, Syria National AIDS Programme (2008) Assessment of HIV Risk and Sero-prevalence among Drug Users in Greater Damascus. Syrian Ministry of Health. UNODC. UNAIDS. Damascus, Syria

30. Morocco Ministry of Health, National Aids Control Program, National Institute of Hygiene, UNAIDS, Global Fund to Fight AIDS Tuberculosis and Malaria (2012) HIV Integrated Behavioral and Biological Surveillance Surveys-Morocco 2011-2012: Injecting Drug Users in Tanger and Nador, Morocco. Rabat, Morocco.

31. Afghanistan National AIDS Control Program (2012) Integrated Behavioral & Biological Surveillance (IBBS) in selected cities of Afghanistan: Findings of 2012 IBBS survey and comparison to 2009 IBBS survey. Johns Hopkins University School of Public Health, National AIDS Control Program, Ministry of Public Health. Kabul, Afghanistan.

32. Parviz S, Fatmi Z, Altaf A, McCormick JB, Fischer-Hoch S, et al. (2006) Background demographics and risk behaviors of injecting drug users in Karachi, Pakistan. Int J Infect Dis 10: 364-371.

33. Nai Zindagi, Punjab Provincial AIDS Control Program (2009) Rapid situation assessments of HIV prevalence and risk factors among people injecting drugs in four cities of the Punjab.

34. Palestine Ministry of Health (2011) HIV bio-behavioral survey among injecting drug users in the East Jerusalem Governorate, 2010.

35. Afghanistan National AIDS Control Program (2010) Integrated Behavioral & Biological Surveillance (IBBS) in Afghanistan: Year 1 Report. HIV Surveillance Project - Johns Hopkins University School of Public Health, National AIDS Control Program, Ministry of Public Health. Kabul, Afghanistan.

36. Todd CS, Abed AM, Strathdee SA, Scott PT, Botros BA, et al. (2007) HIV, hepatitis C, and hepatitis B infections and associated risk behavior in injection drug users, Kabul, Afghanistan. Emerg Infect Dis 13: 1327-1331.

37. Action Aid Afghanistan (2006) A Study on Knowledge, Attitude, Behaviour and Practice in High Risk and Vulnerable Groups in Afghanistan.

38. E. Elghamrawy, O. Abaza, S. Abou Elmagd, H. Ramy, S. Atallah, et al. Risk behaviours among male injecting drug users in Egypt. Abstract no. MOPE227 2012; Washington DC, USA.

39. Malekinejad M, Mohraz M, Razani N, Khairandish P, McFarland W, et al. HIV and related risk behaviors of injecting drug users (IDU) in Iran: findings from the first respondent-driven sampling (RDS) survey of IDU in Tehran in 2006-2007. Abstract no. THAC0202 2008; Mexico.

40. Alavian SM, Mirahmadizadeh A, Javanbakht M, Keshtkaran A, Heidari A, et al. (2013) Effectiveness of methadone maintenance treatment in prevention of hepatitis C virus transmission among injecting drug users. Hepatitis Monthly 13: 9.

41. Amin-Esmaeili M, Rahimi-Movaghar A, Razaghi EM, Baghestani AR, Jafari S (2012) Factors correlated with hepatitis C and B virus infections among injecting drug users in Tehran, IR Iran. Hepatitis Monthly 12: 23-31.

42. Eskandarieh S, Nikfarjam A, Tarjoman T, Nasehi A, Jafari F, et al. (2013) Descriptive Aspects of Injection Drug Users in Iran's National Harm Reduction Program by Methadone Maintenance Treatment. Iran J Public Health 42: 588-593.

43. Honarvar B, Odoomi N, Moghadami M, Afsar Kazerooni P, Hassanabadi A, et al. (2013) Blood-borne hepatitis in opiate users in iran: a poor outlook and urgent need to change nationwide screening policy. PLoS One 8: e82230.

44. Mirahmadizadeh AR, Majdzadeh R, Mohammad K, MH F (2009) Prevalence of HIV and Hepatitis C Virus Infections and Related Behavioral Determinants among Injecting Drug Users of Drop-in Centers in Iran. Iranian Red Crescent Medical Journal 11: 325-329.

45. Kheirandish P, Seyedalinaghi SA, Hosseini M, Jahani MR, Shirzad H, et al. (2010) Prevalence and correlates of HIV infection among male injection drug users in detention in Tehran, Iran. J Acquir Immune Defic Syndr 53: 273-275.

46. Etemad K, Heydari A, Eftekhar Ardabili H, Kabir M.J., Sedaghat M (2010) Knowledge and Attitude Levels in High Risk Groups about HIV/AIDS and Relation with Socioeconomic Level Indicators in Golestan Province [Persian]. Journal of Gorgan University Medical Science 12: 63-69.

47. Rahbar AR, Rooholamini S, Khoshnood K (2004) Prevalence of HIV infection and other blood-borne infections in incarcerated and non-incarcerated injection drug users (IDUs) in Mashhad, Iran. International Journal of Drug Policy 15: 151-155.

48. Mirahmadizadeh A, Kadivar M, Hemmati A, Javadi A (2004) Infection with HIV and hepatitis C and B viruses among injecting drug users in Shiraz, Southern Iran. Abstract no. WePeC5981. AIDS 2004 - XV International AIDS Conference. Bangkok, Thailand.

49. Nokhodian Z, Meshkati M, Adibi P, Ataei B, Kassaian N, et al. (2012) Hepatitis C among intravenous drug users in Isfahan, Iran: A study of seroprevalence and risk factors. International Journal of Preventive Medicine 3: S131-138.

50. Mehrjerdi ZA, Abarashi Z, Noroozi A, Arshad L, Zarghami M (2013) Correlates of shared methamphetamine injection among methamphetamine-injecting treatment seekers: the first report from Iran. Int J STD AIDS.

51. Hassannejad R, Kassaian N, Ataei B, Adibi P (2012) High risky behaviors among intravenous drug users in Isfahan, Iran: A study for hepatitis c harm reduction programs. International Journal of Preventive Medicine 3.

52. Dibaj R, Ataei B, Yaran M, Nokhodian Z, Tayeri K, et al. (2013) Prevalence of HIV infection in inmates with history of injection drug use and evaluation of risk factors, in Isfahan, Iran. Pakistan Journal of Medical Sciences 29: 399-402.

53. Alinaghi SAS, Zadeh AOT, Zaresefat H, Hajizadeh M, Mohamadi SN, et al. (2013) Prevalence of HIV infection and the correlates among beggars in Tehran, Iran. Asian Pacific Journal of Tropical Disease 3: 76-78.

54. Alipour A, Haghdoost AA, Sajadi L, Zolala F (2013) HIV prevalence and related risk behaviours among female partners of male injecting drugs users in Iran: results of a bio-behavioural survey, 2010. Sex Transm Infect 89 Suppl 3: iii41-44.

55. Dastjerdi G, Ebrahimi Dehshiri V, Kholasezade G, Ehsani F (2010) Effectiveness of Methadone in Reduction of High Risk Behaviors in Clients of MMT Center [Persian]. Journal of Shaheed Sadoughi University of Medical Sciences 18: 215-219.

56. Mahfoud Z, Afifi R, Ramia S, El Khoury D, Kassak K, et al. (2010) HIV/AIDS among female sex workers, injecting drug users and men who have sex with men in Lebanon: results of the first biobehavioral surveys. AIDS 24 Suppl 2: S45-54.

57. Ministère de la Santé au Maroc, Direction de l’Épidémiologie et de Lutte contre les Maladies, Programme de lutte contre la toxicomanie (2006) Evaluation rapide de la situation sur le risque d’infection à VIH en relation avec l’usage des drogues injectées et injectables et à problème au Maroc (French) [Rapid situation assessment on the risk of HIV infection associated with the use of injected, injectable, and other drugs in Morocco]. Rabat, Morocco.

58. Stulhofer A, Chetty A, Rabie RA, Jwehan I, Ramlawi A (2012) The Prevalence of HIV, HBV, HCV, and HIV-Related Risk-Taking Behaviors among Palestinian Injecting Drug Users in the East Jerusalem Governorate. J Urban Health 89: 671-676.

59. Ahmad S, Mehmood J, Awan AB, Zafar ST, Khoshnood K, et al. (2011) Female spouses of injection drug users in Pakistan: a bridge population of the HIV epidemic? East Mediterr Health J 17: 271-276.

60. Achakzai M, Kassi M, Kasi PM (2007) Seroprevalences and co-infections of HIV, hepatitis C virus and hepatitis B virus in injecting drug users in Quetta, Pakistan. Trop Doct 37: 43-45.

61. Pakistan National AIDS Control Program (2005) Integrated biological and behavioral surveillance: A Pilot study in Karachi & Rawalpindi 2004-5. Canada-Pakistan HIV/AIDS Surveillance Project. National Aids Control Program, Ministry Of Health, Pakistan. Found at <http://www.nacp.gov.pk/library/reports//Surveillance%20&%20Research//HIV-AIDS%20Surveillance%20Project-HASP/Integrated%20Biological%20&%20Behavioral%20Surveillance%20Pilot%20Study%20in%20Karachi%20&%20Rawalpindi%202005-06.pdf>, Last accessed February 2014.

62. Strathdee SA, Zafar T, Brahmbhatt H, Baksh A, ul Hassan S (2003) Rise in needle sharing among injection drug users in Pakistan during the Afghanistan war. Drug Alcohol Depend 71: 17-24.

63. Nai Zindagi, Punjab Provincial AIDS Control Program (2005) The lethal overdose: Injecting drug use and HIV/AIDS.

64. Abbasi B, Somroo F (2005) HIV outbreak among injecting drug users in larkana, pakistan a serious threat for generalized epidemic. Abstract no. WeOa0305. IAS 2005 - The 3rd IAS Conference on HIV Pathogenesis and Treatment. Rio de Janeiro, Brazil.

65. Ahmed MA, Zafar T, Brahmbhatt H, Imam G, Ul Hassan S, et al. (2003) HIV/AIDS risk behaviors and correlates of injection drug use among drug users in Pakistan. J Urban Health 80: 321-329.

66. Emmanuel F, Attarad A (2006) Correlates of injection use of synthetic drugs among drug users in Pakistan: a case controlled study. J Pak Med Assoc 56: 119-124.

67. Altaf A, Shah SA, Zaidi NA, Memon A, Nadeem ur R, et al. (2007) High risk behaviors of injection drug users registered with harm reduction programme in Karachi, Pakistan. Harm Reduct J 4: 7.

68. Nasir A, Todd CS, Stanekzai MR, Bautista CT, Botros BA, et al. (2011) Prevalence of HIV, hepatitis B and hepatitis C and associated risk behaviours amongst injecting drug users in three Afghan cities. Int J Drug Policy 22: 145-152.

69. Baqi S, Shah SA, Baig MA, Mujeeb SA, Memon A (1999) Seroprevalence of HIV, HBV, and syphilis and associated risk behaviours in male transvestites (Hijras) in Karachi, Pakistan. Int J STD AIDS 10: 300-304.

70. Sharifi-Mood B, Metanat M (2006) Infection among hospitalized injection drug users. Journal of Medical Sciences 6: 686-689.

71. Zamani S, Farnia M, Torknejad A, Alaei BA, Gholizadeh M, et al. (2010) Patterns of drug use and HIV-related risk behaviors among incarcerated people in a prison in Iran. J Urban Health 87: 603-616.

72. Vazirian M, Nassirimanesh B, Zamani S, Ono-Kihara M, Kihara M, et al. (2005) Needle and syringe sharing practices of injecting drug users participating in an outreach HIV prevention program in Tehran, Iran: a cross-sectional study. Harm Reduct J 2: 19.

73. Zamani S, Vazirian M, Nassirimanesh B, Razzaghi EM, Ono-Kihara M, et al. (2010) Needle and syringe sharing practices among injecting drug users in Tehran: a comparison of two neighborhoods, one with and one without a needle and syringe program. AIDS Behav 14: 885-890.

74. Bokhari A, Nizamani NM, Jackson DJ, Rehan NE, Rahman M, et al. (2007) HIV risk in Karachi and Lahore, Pakistan: an emerging epidemic in injecting and commercial sex networks. Int J STD AIDS 18: 486-492.

75. Hermez J, Aaraj E, Dewachi O, Chemaly N HIV/AIDS prevention among vulnerable groups in Beirut, Lebanon. Powerpoint presentation. Lebanon National AIDS Control Program, Beirut, Lebanon.

76. Mir-Nasseri MM, Mohammadkhani A, Tavakkoli H, Ansari E, Poustchi H (2011) Incarceration is a major risk factor for blood-borne infection among intravenous drug users. Hepatitis Monthly 11: 19-22.

77. Platt L, Vickerman P, Collumbien M, Hasan S, Lalji N, et al. (2009) Prevalence of HIV, HCV and sexually transmitted infections among injecting drug users in Rawalpindi and Abbottabad, Pakistan: evidence for an emerging injection-related HIV epidemic. Sex Transm Infect 85 Suppl 2: ii17-22.

78. Osooli M, Khajehkazemi R, Sajadi L, Sedaghat A, Fahimfar N, et al. (2012) HIV prevalence and risk behaviors of adult male injection drug users in Iran; a 2010 national surveillance survey. In press.

79. Haque N, Zafar T, Brahmbhatt H, Imam G, ul Hassan S, et al. (2004) High-risk sexual behaviours among drug users in Pakistan: implications for prevention of STDs and HIV/AIDS. Int J STD AIDS 15: 601-607.

80. Kazerooni PA, Lari MA, Joolaei H, Parsa N (2010) Knowledge and attitude of male intravenous drug users on HIV/AIDS associated high risk behaviors in Shiraz Pir-Banon jail, Fars Province, Southern Iran. Iranian Red Crescent Medical Journal 12: 334-336.

81. Shahroury M (2011) Assessment report on injecting drug users in Jordan. Future Guardians Forum Association, Amman, Jordan.

82. Attia, Medhat S (1996) HIV Seropositivity and KAP towards AIDS among drug addicts in Alexandria. Bull High Inst Public Health 26: 1-8.

83. Khan AA, Rehan N, Qayyum K, Khan A (2008) Correlates and prevalence of HIV and sexually transmitted infections among Hijras (male transgenders) in Pakistan. Int J STD AIDS 19: 817-820.

84. Saleh E, El-Ghazzawi E, El-Sherbini I, Drew W, McFarland W, et al. (1998) Sentinel surveillance for HIV and high risk behaviors among injection drug users in Alexandria, Egypt. Abstract no. 13124. AIDS 1998 - XII International AIDS Conference. Geneva, Switzerland.

85. Davoodian P, Dadvand H, Mahoori K, Amoozandeh A, Salavati A (2009) Prevalence of selected sexually and blood-borne infections in Injecting drug abuser inmates of bandar abbas and roodan correction facilities, Iran, 2002. Braz J Infect Dis 13: 356-358.

86. Nowroozi A, Zali M, Gooya M, Kowsarian P, Raoufi M (1998) The simultanity of HIV, and HBV, HCV and syphilis among the addicted prisoners. Abstract no. 60835. AIDS 1998 - XII International AIDS Conference. Geneva, Switzerland.

87. Agha A, Parviz S, Younus M, Fatmi Z (2003) Socio-economic and demographic factors associated with injecting drug use among drug users in Karachi, Pakistan. J Pak Med Assoc 53: 511-516.

88. Rehan N, Bokhari A, Nizamani NM, Jackson D, Naqvi HR, et al. (2009) National study of reproductive tract infections among high risk groups of Lahore and Karachi. J Coll Physicians Surg Pak 19: 228-231.

89. UrRehman N (2002) Injecting drug use and HIV/AIDS in pakistan. Abstract no. MoPeD3667. AIDS 2002 - XIV International AIDS Conference. Barcelona, Spain.
