# Supplementary material for: HIV among People Who Inject Drugs in the Middle East and North Africa: Systematic Review and Data Synthesis
Source: PLoS Med. 2014 Jun 17;11(6):e1001663. doi: 10.1371/journal.pmed.1001663 (PMC4061009; doi:10.1371/journal.pmed.1001663)
Supplement: Table S7 — HIV/AIDS knowledge, perception of risk, and HIV testing among people who inject drugs in the Middle East and North Africa. (DOCX) [file pmed.1001663.s007.docx]

**Table S7. HIV/AIDS knowledge, perception of risk, and HIV testing among people who inject drugs in the Middle East and North Africa**

|  | **Afghanistan** | **Egypt** | **Iran** | **Jordan** | **Lebanon** | **Morocco** | **Oman** | **OPT** | **Pakistan** | **Syria** | **Tunisia** |
| --- | --- | --- | --- | --- | --- | --- | --- | --- | --- | --- | --- |
| **Ever heard of HIV/AIDS (%)** | 43 [[1](#_ENREF_1)], 83 [[2](#_ENREF_2)], 97 [[2](#_ENREF_2)], 98 [[2](#_ENREF_2)], 100 [[3](#_ENREF_3)], 100 [[3](#_ENREF_3)], 100 [[3](#_ENREF_3)], 100 [[3](#_ENREF_3)], 100 [[3](#_ENREF_3)] | 9 [[4](#_ENREF_4)], 96 [[5](#_ENREF_5)], 99 [[6](#_ENREF_6)], 100 [[6](#_ENREF_6)] | 73 [[7](#_ENREF_7)], 82 [[8](#_ENREF_8)], 93 [[9](#_ENREF_9)], 94 [[9](#_ENREF_9)], 96 [[9](#_ENREF_9)], 100 [[10](#_ENREF_10)] |  |  |  |  |  | 29 [[11](#_ENREF_11)], 52 [[12](#_ENREF_12)], 54 [[13](#_ENREF_13)], 59 [[14](#_ENREF_14)], 65 [[15](#_ENREF_15)], 66 [[16](#_ENREF_16)], 67 [[17](#_ENREF_17)], 72 [[18](#_ENREF_18)], 74 [[19](#_ENREF_19)], 82 [[20](#_ENREF_20)], 86 [[20](#_ENREF_20)], 87 [[21](#_ENREF_21)], 88 [[16](#_ENREF_16)], 89 [[22](#_ENREF_22)], 93 [[23](#_ENREF_23)], 96 [[24](#_ENREF_24)], 97 [[25](#_ENREF_25)] | 99 [[26](#_ENREF_26)] | 100 [[27](#_ENREF_27)], 100 [[28](#_ENREF_28)] |
| **Knowledge of injecting drug use as a mode of transmission (%)** | 51 [[1](#_ENREF_1)], 81 [[2](#_ENREF_2)], 94 [[2](#_ENREF_2)], 97[[2](#_ENREF_2)] | 5 [[4](#_ENREF_4)], 22 [[29](#_ENREF_29)], 33 [[5](#_ENREF_5)], 98 [[6](#_ENREF_6)], 98 [[6](#_ENREF_6)] | 63 [[7](#_ENREF_7)], 85 [[9](#_ENREF_9)], 91 [[9](#_ENREF_9)], 96 [[30](#_ENREF_30)], 96 [[9](#_ENREF_9)], 100 [[31](#_ENREF_31)] | 43 [[32](#_ENREF_32)], 96 [[33](#_ENREF_33)] | 97 [[34](#_ENREF_34)] | 54 [[35](#_ENREF_35)], 90 [[36](#_ENREF_36)], 97 [[36](#_ENREF_36)] |  | 96 [[37](#_ENREF_37)] | 15 [[38](#_ENREF_38)], 46 [[16](#_ENREF_16)], 53 [[16](#_ENREF_16)], 65 [[24](#_ENREF_24)], 68 [[19](#_ENREF_19)], 71 [[18](#_ENREF_18)], 72 [[23](#_ENREF_23)], 77 [[17](#_ENREF_17)], 80 [[15](#_ENREF_15)], 81 [[39](#_ENREF_39)], 82 [[14](#_ENREF_14)], 87 [[21](#_ENREF_21)], 88 [[22](#_ENREF_22)] | 47 [[26](#_ENREF_26)] | 80 [[27](#_ENREF_27)], 93 [[28](#_ENREF_28)] |
| **Knowledge of sex as a mode of transmission (%)** | 78 [[40](#_ENREF_40)] | 88 [[5](#_ENREF_5)] |  | 65 [[32](#_ENREF_32)] |  | 80 [[35](#_ENREF_35)] |  |  | 19 [[38](#_ENREF_38)], 41 [[39](#_ENREF_39)], 41 [[16](#_ENREF_16)], 42 [[16](#_ENREF_16)], 50 [[24](#_ENREF_24)], 72 [[19](#_ENREF_19)], 72 [[23](#_ENREF_23)], 82 [[22](#_ENREF_22)], 84 [[21](#_ENREF_21)], 91 [[17](#_ENREF_17)] | 94 [[26](#_ENREF_26)] |  |
| **Self-perception of risk (%)** |  | 20 [[5](#_ENREF_5)], 29 [[41](#_ENREF_41)] | 43 [[9](#_ENREF_9)], 47 [[9](#_ENREF_9)], 54 [[9](#_ENREF_9)] |  | 45 [[42](#_ENREF_42)], 50 [[34](#_ENREF_34)] | 69 [[35](#_ENREF_35)] |  | 73 [[37](#_ENREF_37)] | 13 [[16](#_ENREF_16)], 30 [[24](#_ENREF_24)], 31 [[19](#_ENREF_19)], 33 [[16](#_ENREF_16)], 34 [[17](#_ENREF_17)], 58 [[18](#_ENREF_18)], 63 [[25](#_ENREF_25)], 64 [[21](#_ENREF_21)] | 27 [[26](#_ENREF_26)] | 63 [[27](#_ENREF_27)] |
| **Ever tested for HIV (%)** | 3 [[3](#_ENREF_3)], 13 [[3](#_ENREF_3)], 16 [[3](#_ENREF_3)], 24 [[2](#_ENREF_2)], 24 [[2](#_ENREF_2)], 33[[2](#_ENREF_2)], 58 [[3](#_ENREF_3)], 60 [[3](#_ENREF_3)] | 1 [[5](#_ENREF_5)], 6 [[43](#_ENREF_43)], 10 [[6](#_ENREF_6)], 10 [[6](#_ENREF_6)] | 28 [[44](#_ENREF_44)], 29 [[8](#_ENREF_8)], 39 [[9](#_ENREF_9)], 44 [[31](#_ENREF_31)], 45 [[10](#_ENREF_10)], 45 [[9](#_ENREF_9)], 47 [[45](#_ENREF_45)], 47 [[46](#_ENREF_46)], 48 [[9](#_ENREF_9)], 60 [[30](#_ENREF_30)], 66 [[47](#_ENREF_47)] | 15 [[32](#_ENREF_32)] 27 [[33](#_ENREF_33)] | 61 [[34](#_ENREF_34)], 80 [[42](#_ENREF_42)] | 13 [[35](#_ENREF_35)], 33 [[36](#_ENREF_36)], 48 [[36](#_ENREF_36)] | 73 [[48](#_ENREF_48)], 94 [[48](#_ENREF_48)], 97 [[48](#_ENREF_48)] | 66 [[37](#_ENREF_37)] | 2 [[12](#_ENREF_12)], 6 [[19](#_ENREF_19)], 21 [[22](#_ENREF_22)], 25 [[21](#_ENREF_21)], 55 [[25](#_ENREF_25)] | 36 [[26](#_ENREF_26)] | 29 [[27](#_ENREF_27)], 30 [[28](#_ENREF_28)] |

OPT: Occupied Palestinian Territories

**References**

1. Action Aid Afghanistan (2006) A Study on Knowledge, Attitude, Behaviour and Practice in High Risk and Vulnerable Groups in Afghanistan.

2. Afghanistan National AIDS Control Program (2010) Integrated Behavioral & Biological Surveillance (IBBS) in Afghanistan: Year 1 Report. HIV Surveillance Project - Johns Hopkins University School of Public Health, National AIDS Control Program, Ministry of Public Health. Kabul, Afghanistan.

3. Afghanistan National AIDS Control Program (2012) Integrated Behavioral & Biological Surveillance (IBBS) in selected cities of Afghanistan: Findings of 2012 IBBS survey and comparison to 2009 IBBS survey. Johns Hopkins University School of Public Health, National AIDS Control Program, Ministry of Public Health. Kabul, Afghanistan.

4. Saleh E, El-Ghazzawi E, El-Sherbini I, Drew W, McFarland W, et al. (1998) Sentinel surveillance for HIV and high risk behaviors among injection drug users in Alexandria, Egypt. Abstract no. 13124. AIDS 1998 - XII International AIDS Conference. Geneva, Switzerland.

5. Elshimi T, Warner-Smith M, Aon M (2004) Blood-borne virus risks of problematic drug users in Greater Cairo. Geneva, UNAIDS & UNODC. August.

6. Family Health International and Ministry of Health Egypt (2010) HIV/AIDS Biological & Behavioral Surveillance Survey: Round Two Summary Report, Cairo, Egypt 2010. FHI in collaboration with the Ministry of Health and support from the Global Fund. Found at <http://www.fhi360.org/sites/default/files/media/documents/BBSS%202010_0.pdf>, Last accessed February 2014.

7. Kazerooni PA, Lari MA, Joolaei H, Parsa N (2010) Knowledge and attitude of male intravenous drug users on HIV/AIDS associated high risk behaviors in Shiraz Pir-Banon jail, Fars Province, Southern Iran. Iranian Red Crescent Medical Journal 12: 334-336.

8. Malekinejad M, Mohraz M, Razani N, Khairandish P, McFarland W, et al. HIV and related risk behaviors of injecting drug users (IDU) in Iran: findings from the first respondent-driven sampling (RDS) survey of IDU in Tehran in 2006-2007. Abstract no. THAC0202 2008; Mexico.

9. Iran Ministry of Health and Medical Education , Kyoto University School of Public Hleath (Japan) (2008) Integrated bio-behavioral surveillance for HIV infection among injecting drug users in Iran. Draft of the 1st analysis on the collected data, Tehran, Iran.

10. Momtazi S, Fallahnejad M, Shoghli A, Musavinasab N, Tavassoli S (2010) HIV high risk behavior in a sample of Iranian injection drug users. Abstract no. TUPE0338. AIDS 2010 - XVIII International AIDS Conference. Vienna, Austria.

11. Nai Zindagi, Punjab Provincial AIDS Control Program (2009) Rapid situation assessments of HIV prevalence and risk factors among people injecting drugs in four cities of the Punjab.

12. Ahmed MA, Zafar T, Brahmbhatt H, Imam G, Ul Hassan S, et al. (2003) HIV/AIDS risk behaviors and correlates of injection drug use among drug users in Pakistan. J Urban Health 80: 321-329.

13. Parviz S, Fatmi Z, Altaf A, McCormick JB, Fischer-Hoch S, et al. (2006) Background demographics and risk behaviors of injecting drug users in Karachi, Pakistan. Int J Infect Dis 10: 364-371.

14. Strathdee SA, Zafar T, Brahmbhatt H, Baksh A, ul Hassan S (2003) Rise in needle sharing among injection drug users in Pakistan during the Afghanistan war. Drug Alcohol Depend 71: 17-24.

15. Nai Zindagi, UNODCCP, UNAIDS. (1999) Baseline study of the relationship between injecting drug use, HIV and Hepatitis C among male injecting drug users in Lahore.

16. Pakistan National AIDS Control Program (2005) Integrated biological and behavioral surveillance: A Pilot study in Karachi & Rawalpindi 2004-5. Canada-Pakistan HIV/AIDS Surveillance Project. National Aids Control Program, Ministry Of Health, Pakistan. Found at <http://www.nacp.gov.pk/library/reports//Surveillance%20&%20Research//HIV-AIDS%20Surveillance%20Project-HASP/Integrated%20Biological%20&%20Behavioral%20Surveillance%20Pilot%20Study%20in%20Karachi%20&%20Rawalpindi%202005-06.pdf>, Last accessed February 2014.

17. Pakistan National AIDS Control Program (2005) HIV Second Generation Surveillance In Pakistan. National Report Round I. Canada-Pakistan HIV/AIDS Surveillance Project. National Aids Control Program, Ministry Of Health, Pakistan. Found at <http://www.nacp.gov.pk/library/reports/Surveillance%20&%20Research/HIV-AIDS%20Surveillance%20Project-HASP/HIV%20Second%20Generation%20Surveillance%20in%20Pakistan%20-%20Round%201%20Report%20-%202005.pdf>, Last accessed February 2014.

18. Bokhari A, Nizamani NM, Jackson DJ, Rehan NE, Rahman M, et al. (2007) HIV risk in Karachi and Lahore, Pakistan: an emerging epidemic in injecting and commercial sex networks. Int J STD AIDS 18: 486-492.

19. Pakistan National AIDS Control Program (2006-07) HIV Second Generation Surveillance In Pakistan. National Report Round II. Canada-Pakistan HIV/AIDS Surveillance Project. National Aids Control Program, Ministry Of Health, Pakistan. Found at <http://www.nacp.gov.pk/library/reports/Surveillance%20&%20Research/HIV-AIDS%20Surveillance%20Project-HASP/HIV%20Second%20Generation%20Surveillance%20in%20Pakistan%20-%20Round%202%20Report%202006-07.pdf>. Last accessed February 2014.

20. Kuo I, ul-Hasan S, Galai N, Thomas DL, Zafar T, et al. (2006) High HCV seroprevalence and HIV drug use risk behaviors among injection drug users in Pakistan. Harm Reduct J 3: 26.

21. Pakistan National AIDS Control Program (2011) HIV Second Generation Surveillance In Pakistan. National Report Round IV. Canada-Pakistan HIV/AIDS Surveillance Project. National Aids Control Program, Ministry Of Health, Pakistan. Found at <http://www.nacp.gov.pk/library/reports/Surveillance%20&%20Research/HIV-AIDS%20Surveillance%20Project-HASP/HIV%20Second%20Generation%20Surveillance%20in%20Pakistan%20-%20National%20report%20Round%20IV%202011.pdf>, Last accessed February 2014.

22. Pakistan National AIDS Control Program (2008) HIV Second Generation Surveillance In Pakistan. National Report Round III. Canada-Pakistan HIV/AIDS Surveillance Project. National Aids Control Program, Ministry Of Health, Pakistan. Found at <http://www.nacp.gov.pk/library/reports/Surveillance%20&%20Research/HIV-AIDS%20Surveillance%20Project-HASP/HIV%20Second%20Generation%20Surveillance%20in%20Pakistan%20-%20National%20report%20Round%20III%202008.pdf>, Last accessed February 2014.

23. Altaf A, Shah SA, Zaidi NA, Memon A, Nadeem ur R, et al. (2007) High risk behaviors of injection drug users registered with harm reduction programme in Karachi, Pakistan. Harm Reduct J 4: 7.

24. Emmanuel F, Attarad A (2006) Correlates of injection use of synthetic drugs among drug users in Pakistan: a case controlled study. J Pak Med Assoc 56: 119-124.

25. Afridi NK, Khan S, Fatima S (2010) Factors affecting accessibility and acceptability of voluntary counselling and testing among high risk group (HRG) for human immunodeficiency virus (HIV) in NWFP Pakistan. J Pak Med Assoc 60: 265-269.

26. Syria Mental Health Directorate, Syria National AIDS Programme (2008) Assessment of HIV Risk and Sero-prevalence among Drug Users in Greater Damascus. Syrian Ministry of Health. UNODC. UNAIDS. Damascus, Syria

27. Tunisia Ministry of Health (2010) Synthèse des enquêtes de séroprévalence et sérocomportementales auprès de trois populations à vulnérables au VIH : Les usagers de drogues injectables, les hommes ayant des rapports sexuels avec des hommes et les travailleuses du sexe clandestines en Tunisie [French]. Synthesis of biobehavioral surveillance among the three populations vulnerable to HIV in Tunisia: Injecting drug users, men who have sex with men, and female sex workers. Tunis, Tunisia.

28. Tunisia Ministry of Health, Tunisian Association for Information and Orientation on HIV (2013) Enquête sérocomportementale du VIH et des hépatites virales C auprès des usagers de drogues injectables en Tunisie [French]. Biobehavioral surveillance of HIV and Hepatitis C among injecting drug users in Tunisia. Tunis, Tunisia.

29. Attia, Medhat S (1996) HIV Seropositivity and KAP towards AIDS among drug addicts in Alexandria. Bull High Inst Public Health 26: 1-8.

30. Vazirian M, Nassirimanesh B, Zamani S, Ono-Kihara M, Kihara M, et al. (2005) Needle and syringe sharing practices of injecting drug users participating in an outreach HIV prevention program in Tehran, Iran: a cross-sectional study. Harm Reduct J 2: 19.

31. Shoghli AR, Mosavi Nasab SN, Fallahnezhad M, Momtazi S, Tavasoli SS, et al. (2011) Behavioral surveillance survey (BSS) among injection drug users (IDUs) in Zanjan-Iran. [Persian]. Journal of Zanjan University of Medical Sciences and Health Services 19: 11.

32. Shahroury M (2011) Assessment report on injecting drug users in Jordan. Future Guardians Forum Association, Amman, Jordan.

33. Jordan National AIDS Program (2010) Preliminary analysis of Jordan IBBSS among injecting drug users. Ministry of Health, Amman, Jordan.

34. Mahfoud Z, Afifi R, Ramia S, El Khoury D, Kassak K, et al. (2010) HIV/AIDS among female sex workers, injecting drug users and men who have sex with men in Lebanon: results of the first biobehavioral surveys. AIDS 24 Suppl 2: S45-54.

35. Ministère de la Santé au Maroc, Direction de l’Épidémiologie et de Lutte contre les Maladies, Programme de lutte contre la toxicomanie (2006) Evaluation rapide de la situation sur le risque d’infection à VIH en relation avec l’usage des drogues injectées et injectables et à problème au Maroc (French) [Rapid situation assessment on the risk of HIV infection associated with the use of injected, injectable, and other drugs in Morocco]. Rabat, Morocco.

36. Morocco Ministry of Health, National Aids Control Program, National Institute of Hygiene, UNAIDS, Global Fund to Fight AIDS Tuberculosis and Malaria (2012) HIV Integrated Behavioral and Biological Surveillance Surveys-Morocco 2011-2012: Injecting Drug Users in Tanger and Nador, Morocco. Rabat, Morocco.

37. Stulhofer A, Chetty A, Rabie RA, Jwehan I, Ramlawi A (2012) The Prevalence of HIV, HBV, HCV, and HIV-Related Risk-Taking Behaviors among Palestinian Injecting Drug Users in the East Jerusalem Governorate. J Urban Health 89: 671-676.

38. Haque N, Zafar T, Brahmbhatt H, Imam G, ul Hassan S, et al. (2004) High-risk sexual behaviours among drug users in Pakistan: implications for prevention of STDs and HIV/AIDS. Int J STD AIDS 15: 601-607.

39. Altaf A, Shah SA, A. M (2003) Follow up study to assess and evaluate knowledge, attitude and high risk behaviors and prevalence of HIV, HBV, HCV and Syphilis among IDUS at Burns Road DIC, Karachi. External report submitted to UNODC.

40. Todd CS, Abed AM, Strathdee SA, Scott PT, Botros BA, et al. (2007) Association between expatriation and HIV awareness and knowledge among injecting drug users in Kabul, Afghanistan: A cross-sectional comparison of former refugees to those remaining during conflict. Confl Health 1: 5.

41. Elsawy HF, Al-Kabash IM (2011) Risky behaviors of HIV infection among drug dependents in Egypt. European Psychiatry 26.

42. Aaraj E Report on the situation analysis on vulnerable groups in Beirut, Lebanon. Lebanon Ministry of Health, Beirut, Lebanon.

43. Soliman C, Rahman IA, Shawky S, Bahaa T, Elkamhawi S, et al. (2010) HIV prevalence and risk behaviors of male injection drug users in Cairo, Egypt. AIDS 24 Suppl 2: S33-38.

44. Zamani S, Kihara M, Gouya MM, Vazirian M, Ono-Kihara M, et al. (2005) Prevalence of and factors associated with HIV-1 infection among drug users visiting treatment centers in Tehran, Iran. AIDS 19: 709-716.

45. Zamani S, Farnia M, Torknejad A, Alaei BA, Gholizadeh M, et al. (2010) Patterns of drug use and HIV-related risk behaviors among incarcerated people in a prison in Iran. J Urban Health 87: 603-616.

46. Zamani S, Radfar R, Nematollahi P, Fadaie R, Meshkati M, et al. (2010) Prevalence of HIV/HCV/HBV infections and drug-related risk behaviours amongst IDUs recruited through peer-driven sampling in Iran. Int J Drug Policy 21: 493-500.

47. Mirahmadizadeh AR, Majdzadeh R, Mohammad K, MH F (2009) Prevalence of HIV and Hepatitis C Virus Infections and Related Behavioral Determinants among Injecting Drug Users of Drop-in Centers in Iran. Iranian Red Crescent Medical Journal 11: 325-329.

48. Oman Ministry of Health (2006) HIV Risk among Heroin and Injecting Drug Users in Muscat, Oman. Quantitative Survey. Preliminary Data. Muscat, Oman.
