# Supplementary material for: HIV among People Who Inject Drugs in the Middle East and North Africa: Systematic Review and Data Synthesis
Source: PLoS Med. 2014 Jun 17;11(6):e1001663. doi: 10.1371/journal.pmed.1001663 (PMC4061009; doi:10.1371/journal.pmed.1001663)
Supplement: Text S2 — Search criteria. (DOCX) [file pmed.1001663.s009.docx]

**Text S2**

**Search criteria**

The following criteria were used to search the different data sources.

**Pubmed**

("Drug Users"[Mesh] OR "Substance Abuse, Intravenous"[Mesh] OR "Needle Sharing"[Mesh] OR "Heroin Dependence"[Mesh] OR Drug use*[Text] OR Drug-use*[Text] OR Substance abuse*[Text] OR Drug abuse*[Text] OR Drug-abuse*[Text] OR Injecting-drug use*[Text] OR Intravenous-drug use*[Text] OR Injection-drug use*[Text] OR IDU*[Text] OR IVDU*[Text] OR Drug addict*[Text] OR Drug dependen*[Text]) **AND** ("Middle East"[Mesh] OR "Islam"[Mesh] OR "Arabs"[Mesh] OR "Arab World"[Mesh] OR "Africa, Northern"[Mesh] OR "Sudan"[Mesh] OR "Somalia"[Mesh] OR "Djibouti"[Mesh] OR "Pakistan"[Mesh] OR "Middle East"[Text] OR "Middle-East"[Text] OR "North Africa"[Text] OR "North-Africa"[Text] OR "EMRO"[Text] OR "Eastern Mediterranean"[Text] OR “Arab”[Text] OR “Arabs”[Text] OR “Arab World”[Text] OR "Islam"[Text] OR "Afghanistan"[Text] OR "Algeria"[Text] OR "Bahrain"[Text] OR "Djibouti"[Text] OR "Egypt"[Text] OR "Jordan"[Text] OR "Kuwait"[Text] OR "Lebanon"[Text] OR "Libya"[Text] OR "Iran"[Text] OR "Iraq"[Text] OR "Morocco"[Text] OR "Oman"[Text] OR "Pakistan"[Text] OR "Qatar"[Text] OR "Saudi Arabia"[Text] OR "Somalia"[Text] OR "Sudan"[Text] OR "Syria"[Text] OR "Tunisia"[Text] OR "United Arab Emirates"[Text] OR "Dubai"[Text] OR "Abu Dhabi"[Text] OR "Abu-Dhabi"[Text] OR “Sharjah”[Text] OR "West Bank"[Text] OR "Ghaza"[Text] OR "Palestine"[Text] OR"Yemen"[Text])

**Embase**

(exp drug abuse/ or exp substance abuse/ or exp drug dependence/ or exp heroin dependence/ or (drug use* or drug abuse* or drug addict* or drug dependen* or IDU* or IVDU*).mp.) AND (exp Middle East/ or exp North Africa/ or exp Arab/ or exp Afghanistan/ or exp Djibouti/ or exp Pakistan/ or exp Somalia/ or exp Sudan/ or Middle East.mp. or North Africa.mp. or EMRO.mp. or Eastern Mediterranean.mp. or Arab.mp. or Arabs.mp. or Arab World.mp. or Islam.mp. or Afghanistan.mp. or Algeria.mp. or Bahrain.mp. or Djibouti.mp. or Egypt.mp. or Jordan.mp. or Kuwait.mp. or Lebanon.mp. or Libya.mp. or Iran.mp. or Iraq.mp. or Morocco.mp. or Oman.mp. or Pakistan.mp. or Qatar.mp. or Saudi Arabia.mp. or Somalia.mp. or Sudan.mp. or Syria.mp. or Tunisia.mp. or United Arab Emirates.mp. or Dubai.mp. or Abu Dhabi.mp. or Sharjah.mp. or West Bank.mp. or Ghaza.mp. or Palestine.mp. or Yemen.mp.)

**Regional databases**

WHO African Index Medicus: Free text search using each MENA country name

WHO Index Medicus for the Eastern Mediterranean Region: A keyword search of “injecting” and a keyword search of “HIV”

**Conference abstracts**

Free text search using each MENA country name
