# Supplementary material for: HIV among People Who Inject Drugs in the Middle East and North Africa: Systematic Review and Data Synthesis
Source: PLoS Med. 2014 Jun 17;11(6):e1001663. doi: 10.1371/journal.pmed.1001663 (PMC4061009; doi:10.1371/journal.pmed.1001663)
Supplement: Text S3 — Narrative justification for quality of the evidence and status of the epidemic at the country level. (DOCX) [file pmed.1001663.s010.docx]

**Text S3**

**Narrative justification for quality of the evidence and status of the epidemic at the country level**

This text relates to Table 5 in main manuscript. Countries are sorted by level of HIV prevalence, trend in HIV prevalence, geographical distribution, quality and scope of evidence, then alphabetical order.

**Iran**

The number of studies conducted is the largest in MENA and the number of HIV prevalence measures is also substantial, being the second largest after Pakistan. The geographical coverage of conducted studies is national, with a large number of cities/provinces represented. Two rounds of surveillance have also been conducted in 2006 and 2010 which included up to 10 cities each, had a large sample size, and used the probability-based time-location sampling technique. In total, about half of HIV prevalence measures in Iran were from studies using probability-based sampling methodologies and the vast majority had high precision.

The first HIV outbreaks among PWID in Iran were reported around 1996, and it is only in the early 2000s that HIV prevalence started increasing considerably to reach a peak by the mid-2000s (Figure 3A). HIV transmission among PWID after 2005 is still ongoing and seemingly at high levels, but a trend of increasing HIV prevalence does not seem to be apparent. Injecting drug use remains the major mode of transmission in notified HIV cases at 60% in 2011. The totality of the evidence suggests that the HIV epidemic among PWID in Iran is now established at concentrated levels of about 15%.

**Pakistan**

The number of studies and of HIV prevalence measures is substantial, the latter being the largest in MENA. The geographical coverage of conducted studies is national, with a large number of cities/districts represented. Over one-third of studies conducted included multiple locations. The quality of HIV prevalence measures is good: two-thirds were from studies using probability-based sampling techniques, over half reached their target sample size, and the overwhelming majority had high precision. Pakistan had also four rounds of repeated integrated bio-behavioral surveillance surveys (IBBSS), the highest number in MENA. These surveys used multi-stage cluster sampling, included up to 16 cities per round, and were preceded and informed by ethnographic mapping.

The totality of the evidence indicates that after almost two decades of very limited HIV prevalence among PWID, a trend of increasing prevalence started to be observed after 2003 (Figure 3B). One incidence study conducted in 2002 among PWID in three cities reported an HIV incidence rate of 1.7 per 100 person-years. The trend of increasing HIV prevalence seems to be ongoing, reaching over 40% in several studies, and with no evidence of stabilization or peak in the most recent studies. The trend of an emerging epidemic is also manifest in the repeated rounds of IBBSS using standard and state of the art methodology: HIV prevalence among PWID has steadily increased from 10.8% in 2005, to 15.8% in 2006, to 20.8% in 2008, and reached 25.2% in 2011. There is conclusive evidence of a concentrated emerging HIV epidemic among PWID at the national level in Pakistan.

**Afghanistan**

A number of well-designed studies have been conducted in Afghanistan, some with respondent-driven sampling (RDS) and others with thoughtful convenience sampling using a variant of time-location sampling. Two round of IBBSS have been implemented in 2009 and 2012. Data are available from four main cities in Afghanistan and overall all studies were adequately powered.

Although data from the earlier years of the epidemic are not available, the first studies conducted in 2005-8 reported low HIV prevalence among PWID at 0-3%. However, a substantial increase in HIV prevalence reaching up to 18% in one city, Herat, was reported in the IBBSS conducted in 2009 and confirmed in the second round at 13.3%. HIV incidence among PWID in Kabul in 2008 was also reported at 2.2 per 100 person-years, despite 72% reported use of harm reduction services among participants.

The totality of the evidence suggests that the epidemic in Afghanistan is recent, emerging in the last few years, and has reached concentrated levels in at least some parts of Afghanistan. Herat is close to the Afghani-Iranian border and molecular investigations have found the same HIV variants among PWID in Iran and in Kabul, Afghanistan, far away from the Iranian border. This suggests that the virus may have been introduced by the return of Afghani refugees from Iran. Iran had already a large HIV epidemic among PWID since the early 2000s. More data and from other cities are needed to confirm the observed trends at the national level.

**Egypt**

The number of studies conducted is small, but overall of good quality. Prominently, two rounds of repeated IBBSS were conducted. These were adequately powered and used the state of art sampling methodology of RDS. In addition, there is a large number of HIV prevalence measures that were extracted from databases.

The totality of the evidence indicates very limited HIV prevalence among PWID for about two decades, including in the first round of IBSS in 2006, but a noticeable increase reaching about 7% in the most recent round of surveillance in 2010. Available HIV prevalence measures however cover mainly the two largest cities in Egypt and therefore the evidence cannot be generalized to the national level. Nonetheless, an emerging epidemic among PWID is also apparent in HIV case notification reports whereby 19.6% of notified HIV cases in 2010 were due to injecting drug use, compared to 1.6% of the total notified cases since the beginning of the epidemic until 2008. More data and from various parts of the country are needed to confirm the observed emerging epidemic among PWID at the national level.

**Morocco**

The number of studies conducted in Morocco is small, but of relatively good quality. With the exception of one point-prevalence measure in the 1990s with unclear methodology and quality, the recent studies conducted in 2008 and 2011 are well-designed, well-powered, and used RDS as a sampling strategy. There is also a number of HIV prevalence measures from databases, including voluntary counseling and treatment (VCT) data and data from sentinel surveillance.

Overall, the data indicate very low HIV prevalence among PWID until recently, starting 2008, when both VCT data and the well-designed RDS studies started to indicate substantial prevalence, particularly in one city, Nador, where HIV prevalence has been reported at 25-38%. It is worth noting that HIV prevalence in the other cities included in the recent RDS studies is still at zero prevalence, suggesting that the epidemic in Morocco is only emerging and still highly localized. The recent nature of the epidemic is also confirmed by HIV case notifications, whereby the contribution of injecting drug use to notified cases in 2011 was only 1.2%. More data and from various parts of the country are needed to confirm the observed trends at the national level.

**Libya**

Libya has just completed its first study investigating the epidemiology of HIV among PWID. This study is the first round of a planned IBBSS, used RDS, and had a large sample size. There is in addition two data points that were extracted from the various databases. Though the methodology of these data points is unclear, they indicated substantial HIV prevalence at 22.0% and 59.4%, suggesting major outbreaks of HIV among PWID in Libya possibly around the beginning of the last decade or even earlier. These data are now confirmed by the noted first round of IBBSS which reported an alarming HIV prevalence of 87.1% among PWID in Tripoli, the highest prevalence reported in MENA. The evidence therefore is indicative of a concentrated HIV epidemic among PWID in at least part of Libya. Though the epidemic in Tripoli with all likelihood is an established epidemic, the level of evidence overall is not enough to characterize whether the national epidemic is emerging, with few outbreaks in the past; or has been established for some time now with endemic HIV transmission among PWID. The good quality data is restricted to one city, Tripoli, and therefore more data are needed from various parts of the country to indicate whether there is a concentrated epidemic among PWID at the national level.

**Bahrain**

There is a number of HIV prevalence measures, the vast majority of which are from databases and therefore of unclear methodology and quality. There is only one study which was conducted and for which a report was available. This study had a large sample size and was conducted in a voluntary drug treatment center. It reported a prevalence of 21.1% in Manama in the early 90s. The data from the databases also indicated some HIV spread among PWID in Bahrain with prevalence rates reaching up to 8%. This is further reflected in HIV case notification reports, whereby in 2010, 37.5% of notified HIV cases were due to injecting drug use, suggesting ongoing transmission. The totality of the evidence suggests that there are at least some pockets of HIV among PWID in Bahrain, but the type and quality of available evidence is not enough to indicate whether there is a concentrated epidemic, even if localized.

**Oman**

There is a number of HIV prevalence measures, the vast majority of which are from databases and therefore of unclear methodology and quality. There is only one study which was conducted and for which a report was available, but with high risk of bias in all domains, including self-reported HIV prevalence. However, the reported HIV prevalence in this study, and the data extracted from the databases, indicate substantial levels of HIV prevalence reaching up to 27%. Still, the contribution of injecting drug use to the total notified HIV cases remains small at 4.3% until the end of 2011. The totality of the evidence therefore suggests that there have been at least some pockets of HIV among PWID in Oman, but the type and quality of available evidence is not sufficient to indicate whether there is a concentrated epidemic, even if localized.

**Jordan**

There is a number of HIV prevalence measures from databases starting from 1990, in addition to one round of IBBSS in 2009 that used RDS and covered four cities. All prevalence measures indicated zero prevalence among PWID. This is further reflected in case notifications whereby in 2011, 0% of the notified HIV cases were due to injecting drug use. The totality of the evidence therefore suggests that the HIV epidemic among PWID in Jordan is a low-level epidemic.

**Lebanon**

The number of HIV prevalence measures in Lebanon is small, but includes a round of IBBSS that used RDS and was conducted in 2007-8. The study found zero HIV prevalence among PWID. Although this study failed to reach its target sample size, possibly due to disconnectivity in the PWID networks in this country, all the other data available, whether from studies or databases, report very low HIV prevalence among PWID. This is also reflected in the HIV case notifications, whereby in 2011, 2% of notified cases were due to injecting drug use. The totality of the evidence therefore points towards a low-level HIV epidemic among PWID in Lebanon.

**Occupied Palestinian Territories (OPT)**

The number of HIV prevalence measures among PWID in the OPT is very small, but includes a first round of IBBSS that used RDS. The study found no infections among PWID in East Jerusalem, suggesting that the epidemic is at low-level. Also, only 2.8% of notified AIDS cases until the end of 2011 were due to injecting drug use. More data and from various parts of the country are needed to confirm this low-level epidemic state at the national level.

**Tunisia**

Two rounds of IBBSS have been conducted among PWID in Tunisia in 2009 and 2011. The studies had a large sample size and used RDS. They reported an HIV prevalence of 3.1% and 2.4%, respectively. The several HIV prevalence measures extracted from databases indicate limited prevalence since 1992. Still, by the end of 2009, 24.4% of all notified cases were due to injecting drug use, suggesting that HIV transmission is ongoing among PWID in Tunisia. The totality of the evidence therefore suggests that although the HIV epidemic among PWID in Tunisia appears to be a low-level epidemic, there could be somewhat significant ongoing HIV transmission among PWID. This low-intensity epidemic however does not appear to have reached high enough levels to be qualified as a concentrated epidemic.

**Syria**

A number of HIV prevalence measures among PWID in Syria are available from databases from 1988 and until 2007. All of them indicate zero prevalence. In addition, there is one study which was conducted in Damascus using snow-ball sampling. The study reported a prevalence of 0.5%. The limited HIV prevalence among PWID is confirmed in case notifications whereby in 2011, 0% of the notified HIV cases were due to injecting drug use. The totality of the evidence therefore suggests that the HIV epidemic among PWID in Syria is a low-level epidemic. More data and from various parts of the country are needed to confirm this epidemic state at the national level.
